# Rifamycin congeners kanglemycins are active against rifampicin-resistant bacteria via a distinct mechanism

**DOI:** 10.1038/s41467-018-06587-2

**Published:** 2018-10-08

**Authors:** James Peek, Mirjana Lilic, Daniel Montiel, Aleksandr Milshteyn, Ian Woodworth, John B. Biggins, Melinda A. Ternei, Paula Y. Calle, Michael Danziger, Thulasi Warrier, Kohta Saito, Nathaniel Braffman, Allison Fay, Michael S. Glickman, Seth A. Darst, Elizabeth A. Campbell, Sean F. Brady

**Affiliations:** 1 Laboratory of Genetically Encoded Small Molecules, The Rockefeller University, 1230 York Avenue, New York, NY 10065 USA; 2 Laboratory of Molecular Biophysics, The Rockefeller University, 1230 York Avenue, New York, NY 10065 USA; 3 Department of Microbiology and Immunology, Weill Cornell Medicine, New York, NY 10065 USA; 4 Department of Medicine, Weill Cornell Medicine, New York, NY 10065 USA; 5 Immunology Program, Sloan-Kettering Institute, New York, NY 10065 USA

## Abstract

Rifamycin antibiotics (Rifs) target bacterial RNA polymerases (RNAPs) and are widely used to treat infections including tuberculosis. The utility of these compounds is threatened by the increasing incidence of resistance (Rif^R^). As resistance mechanisms found in clinical settings may also occur in natural environments, here we postulated that bacteria could have evolved to produce rifamycin congeners active against clinically relevant resistance phenotypes. We survey soil metagenomes and identify a tailoring enzyme-rich family of gene clusters encoding biosynthesis of rifamycin congeners (kanglemycins, Kangs) with potent in vivo and in vitro activity against the most common clinically relevant Rif^R^ mutations. Our structural and mechanistic analyses reveal the basis for Kang inhibition of Rif^R^ RNAP. Unlike Rifs, Kangs function through a mechanism that includes interfering with 5′-initiating substrate binding. Our results suggest that examining soil microbiomes for new analogues of clinically used antibiotics may uncover metabolites capable of circumventing clinically important resistance mechanisms.

## Abstract (web summary)

Resistance to rifamycin antibiotics, which target bacterial RNA polymerases, is a growing problem. Here, the authors identify gene clusters from soil metagenomes encoding production of rifamycin analogues that are active against rifampicin-resistant bacteria through a distinct mechanism.

## Introduction

Semisynthetic derivatives of the bacterial natural product rifamycin (e.g., rifampicin or Rif) are components in the first-line treatment of tuberculosis and other gram-positive bacterial infections^1^. As with many antibiotics, the clinical utility of these therapeutics has declined due to the increased incidence of antibiotic resistant bacterial pathogens^2^. Resistance to the rifamycin family of antibiotics commonly occurs in clinical isolates as a result of point mutations in the antibiotic’s target, the DNA-dependent RNA polymerase (RNAP)^3^. These mutations, as well as other clinically relevant antibiotic resistance mechanisms, are also likely to be present in natural environments where they would have evolved in response to antibiotics produced by other bacteria^4–6^. The search for biologically active bacterial natural products has frequently led to the discovery of families of structurally related antibiotics (congeners) that arise from evolutionarily related biosynthetic gene clusters. While these close analogues typically have the same molecular target, they often exhibit different biological activities, including differences in potency, spectrum of activity and activity against resistant bacteria^7–9^. In this study, we postulated that competition between environmental microbes might have selected for the evolution of rifamycin congeners capable of circumventing common antibiotic resistance mechanisms, including those enriched in clinical settings, providing a source of new therapeutics to treat rifamycin resistant bacteria.

In an effort to understand natural rifamycin biosynthetic diversity we turned to the sequencing of soil metagenomes. Soils are believed to be a rich and underexplored reservoir of bacterial biosynthetic diversity, with each gram of soil containing thousands of previously unstudied bacterial species^10,11^. The development of robust sequencing approaches for identifying biosynthetic gene clusters in complex microbiomes has made it possible to systematically explore soil ecosystems for gene cluster families of interest^12,13^. We hypothesized that the most biosynthetically complex rifamycin-like gene clusters found in soil environments could represent nature’s most evolved responses to commonly encountered rifamycin resistance mechanisms. Our survey of soil metagenomes revealed a rich diversity in rifamycin biosynthesis. One family of gene clusters, which contained the largest collection of predicted tailoring genes, was of particular interest to us, as we expected it might encode for the most highly functionalized rifamycin congeners. We identified numerous examples of this gene cluster family in soil metagenomes as well as one example in the sequenced genome of a cultured bacterium. Here, we report on the characterization of kanglemycin-like rifamycin congeners, kanglemycin (Kang) A, V1, and V2, that are encoded by a member of this tailoring enzyme-rich gene cluster family.

All three Kangs were more potent than Rif when assayed against bacteria carrying RNAP mutations corresponding to those commonly identified in Rif resistant (Rif^R^) clinical isolates of *Mycobacterium tuberculosis* (*Mtb*), the causative agent of tuberculosis. Interestingly, Kang V1 and V2 exhibit their highest levels of activity against bacteria carrying different common Rif^R^ mutations. To study the inhibition of RNAP by Kang-like congeners, we solved X-ray co-crystal structures of Kang A in complex with both wild-type *Mycobacterium smegmatis* (*Msm*) RNAP and a Rif^R^ RNAP variant carrying the most commonly observed Rif^R^ mutation found in *Mtb* clinical isolates (*Msm* RNAP βS447L). Our structural analysis revealed that Kang A binds the same site on RNAP as Rif. Additional interactions between the chemical groups unique to the Kangs (compared to Rif) and RNAP help to explain the ability of these compounds to inhibit Rif^R^ RNAP. The structural results combined with structure-guided biochemical studies indicate that the Kangs inhibit RNAP activity at a step of transcription upstream of the step inhibited by Rif. Taken together, these data suggest that the Kangs V1 and V2 represent potential lead structures for the development of therapeutics with activity against Rif^R^ bacteria.

## Results

### Metagenomic survey of rifamycin biosynthetic diversity

**Fig. 1:**
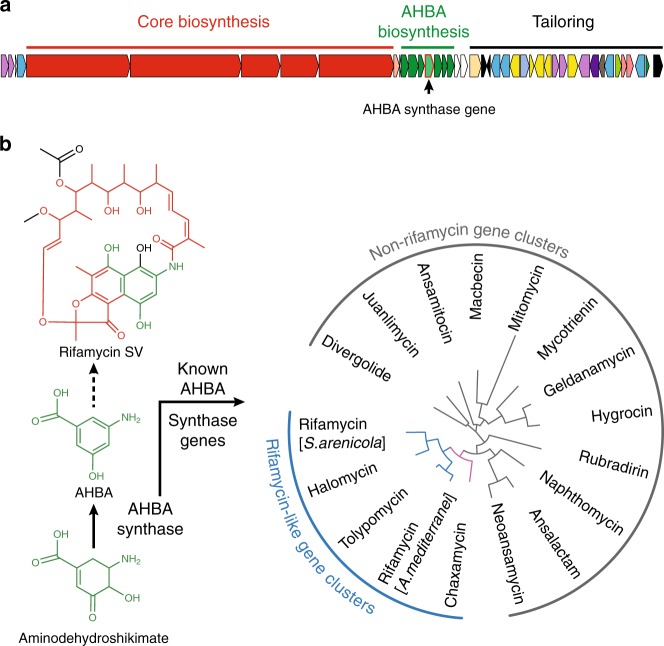
The rifamycin biosynthetic gene cluster and the role of AHBA synthase. **a** The rifamycin gene cluster from *Amycolatopsis mediterranei*. **b** The reaction catalyzed by AHBA synthase and the structure of rifamycin SV (the product of the rifamycin gene cluster). The rifamycin SV structure is colored according to the genes responsible for producing its PK core (red), AHBA-derived substructure (green), and tailoring functionalities (black). The phylogenetic divergence of AHBA synthase genes from previously characterized gene clusters correlates with the different structural classes of ansamycins

The complexity of soil microbiomes limits the utility of shotgun sequencing as a tool for identifying biosynthetic gene clusters in soil metagenomes. Instead, PCR-based methods that use degenerate primers to target conserved natural product biosynthetic genes have been developed to study the biosynthetic gene cluster diversity present in an environmental sample, much in the same way that bacterial phylogenetic diversity is routinely evaluated through the analysis of PCR-amplified 16S genes^12,13^. To assess the diversity of rifamycin-like gene clusters present in soil microbiomes, we used degenerate primers targeting the 3-amino-5-hydroxy benzoic acid (AHBA) synthase gene, which encodes the final step in AHBA biosynthesis (Fig. 1). AHBA is the universal precursor for the ansamycin family of natural products, including the rifamycins^14^. The phylogenetic divergence of AHBA synthase genes correlates closely with the structural divergence of the metabolites encoded by the biosynthetic gene clusters from which an AHBA synthase gene arises, making it an information-rich target for identifying rifamycin-like gene clusters in metagenomes using PCR-based methods (Fig. 1)^15–19^.

**Fig. 2:**
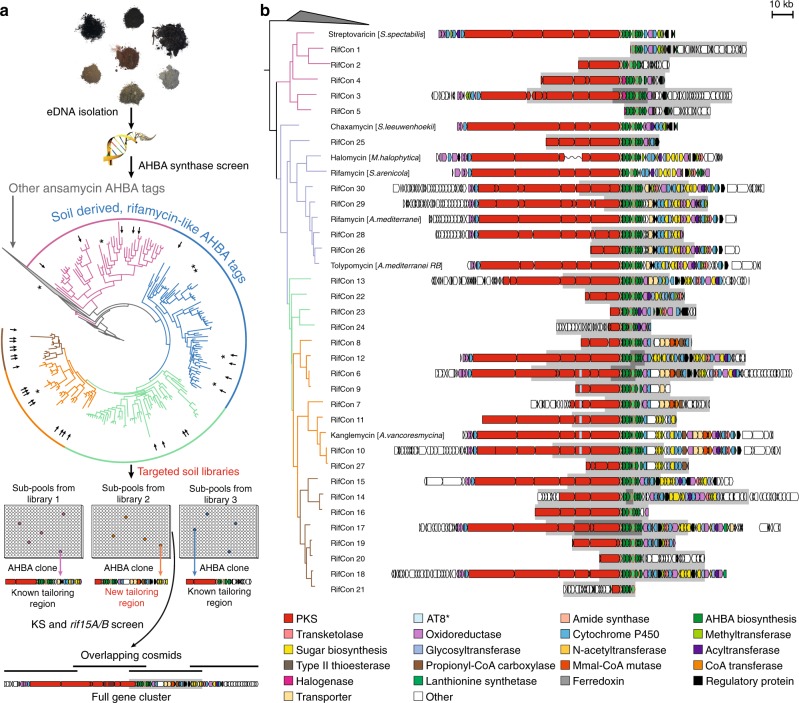
Sequence-based screen for rifamycin congener gene clusters. **a** Screening overview. DNA isolated from ~1500 soils was screened for the presence of AHBA synthase genes by PCR using degenerate primers. Sequence tags generated in this screen were used to construct a phylogenetic tree, onto which AHBA synthase reference sequences from known rifamycin congener gene clusters were mapped (marked with asterisks). Large, distinct clades in the phylogenetic tree are shown in different colors. Metagenomic DNA cosmid libraries were generated from soils that contained AHBA sequence tags that spanned all AHBA clades predicted to be associated with rifamycin congener gene clusters. To facilitate the recovery of individual clones containing gene clusters of interest, each metagenomic library was expanded to contain >20,000,000 unique eDNA cosmids and formatted as smaller subpools of between 20,000 and 60,000 unique cosmid clones per sub-pool. Primary clones (those containing an AHBA synthase gene) were recovered from AHBA positive subpools using a PCR dilution method and degenerate AHBA synthase primers. The same approach, but with degenerate primers targeting PKS ketosynthase (KS) domains and the *rif15A/15B* tailoring genes, was used to recover regions of the pathways that flank those found on the primary clone. AHBA sequence tags corresponding with primary clones that were targeted for recovery are indicated with arrows on the phylogenetic tree. **b** Summary of rifamycin congener gene clusters recovered from the soil metagenomes. Portions of the gene clusters found on primary clones are shown on a gray background

To identify metagenomes containing rifamycin-like biosynthetic gene clusters, environmental DNA (eDNA) isolated from a collection of approximately 1500 geographically and ecologically diverse soils was used as the template in PCR reactions with degenerate primers designed to amplify AHBA synthase genes (Fig. 2a; Supplementary Table 1). Amplicon sequences generated from each soil were then compared to a reference collection of AHBA synthase genes from characterized ansamycin biosynthetic gene clusters. A soil was considered a potential source of a rifamycin-like gene cluster if it contained an AHBA synthase sequence that was more closely related to a gene from a known rifamycin-like gene cluster than from any other ansamycin family gene cluster (Fig. 2a). Based on this analysis, rifamycin-like biosynthetic gene clusters were present in approximately half of the soils we examined. AHBA synthase amplicons within the rifamycin-like sequence-space form a number of well-defined clades (Fig. 2a), which we predicted might be associated with groups of biosynthetic gene clusters encoding structurally distinct congeners. To access the potentially new rifamycin-like gene clusters from soil metagenomes, we constructed saturating cosmid-based metagenomic libraries from seven soils. This subset of soils yielded a multitude of distinct AHBA synthase sequences, which were predicted to span all of the major rifamycin-like clades that we identified.

### Rifamycin-like gene clusters from metagenomic libraries

In sequenced biosynthetic gene clusters that encode rifamycin family members, a variable region containing tailoring genes, responsible for generating most of the structural diversity seen in rifamycin congeners, resides directly downstream to the AHBA biosynthesis operon (Fig. 1). To guide the isolation of eDNA cosmids containing tailoring genes, the seven newly constructed and two pre-existing soil eDNA libraries were screened with the same AHBA synthase degenerate primers that we used to screen crude eDNA extracts (Fig. 2a). We initially recovered 35 unique cosmids (i.e., primary clones) from sublibrary pools that yielded rifamycin-like AHBA sequences (Fig. 2b). Sequencing of these cosmids revealed that variations in the collections of predicted tailoring genes largely changed in concert with the phylogenetic divergence of the AHBA synthase genes.

Representative gene clusters associated with each major AHBA synthase clade were recovered in their entirety on sets of overlapping cosmid clones (Fig. 2a). Each collection of overlapping cosmids was sequenced, assembled into a single continuous stretch of DNA and annotated in silico to reveal an eDNA-derived rifamycin-like gene cluster (Fig. 2b). eDNA-derived gene clusters were predicted to encode a number of enzymes that have not previously been associated with rifamycin congener biosynthesis (e.g., *N*-acyltransferases, CoA-transferases, propionyl-CoA carboxylases, methylmalonyl-CoA mutases, and lanthionine synthetase-like enzymes) (Fig. 2b and Supplementary Figure 1A). A number of other tailoring genes found in these clusters are phylogenetically distinct from those found in known rifamycin-like gene clusters, suggesting they may differentially functionalize the rifamycin backbone. These genes are predicted to encode glycosyltransferases, methyltransferases, cytochrome P450s, oxidoreductases, and sugar biosynthesis enzymes (Supplementary Figures 2 and 3).

**Fig. 3:**
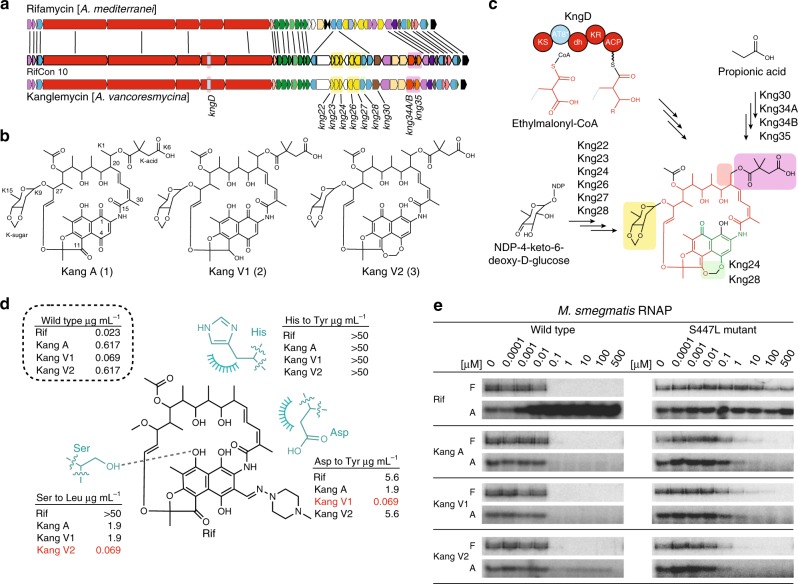
Analysis of the *kng* gene cluster and the activity of Kangs A, V1, and V2. **a** Comparison of the rifamycin (*rif*) and Kang (*kng*) gene clusters. Lines connecting the two clusters indicate genes that are predicted to be functionally equivalent. For simplicity, only genes lacking a counterpart in the *rif* cluster are labeled in the *kng* cluster. Colored boxes surrounding these genes correspond to the substructures they are predicted to encode (shown in panel C). **b** Structures of Kangs A, V1 and V2. **c** Summary of the proposed biosynthesis of Kang V2. The structure of Kang V2 is colored as follows: red, PK core; blue, Emal modification; green, AHBA-derived substructure; black, tailoring modifications. Colored bubbles highlighting the key structural features of Kang V2 correspond with the genes in (A) that are predicted to encode for these features. The PKS module 8 dehydratase (dh) domain, which is predicted to be inactive, is shown in lower case letters to differentiate it from the remaining, active domains. **d** In vivo activity profiles of the Kangs against Rif^R^ *Sau*. The structure of Rif is shown along with the three most commonly mutated RNAP residues in Rif^R^ *Mtb* clinical isolates^3, 29^. The dashed line and arcs indicate an H-bond and nonpolar contacts, respectively. **e**. In vitro transcription assay with radiolabeled nucleotides showing the activity of Rif and the Kangs at the concentrations indicated against *Msm* wild-type and Rif^R^ βS447L RNAP. F, full-length transcript; A, abortive transcript

In most cases, the polyketide synthase (PKS) portion of each gene cluster is predicted to be functionally identical. However, a number of gene clusters with the most complex sets of tailoring genes were predicted to encode a change in the substrate specificity of the acyltransferase (AT) domain in the eighth PKS module (AT8*, Fig. 2b). These AT8* domains are predicted to use ethylmalonyl-CoA (Emal) as a substrate instead of methylmalonyl-CoA (Mmal)^20,21^, which would introduce a two-carbon branch into the rifamycin polyketide (PK) core (Supplementary Figure 1B). The combination of a potential change in the PK core structure and a complex collection of tailoring genes led us to prioritize this family of gene clusters for investigation. We hypothesized that these gene clusters would encode the most complex rifamycin congeners to have evolved to date and that this increased complexity might have evolved in response to common rifamycin resistance mechanisms. Based on AHBA synthase phylogeny, 13% of the rifamycin-like AHBA synthase sequences we amplified from soil environments are predicted to arise from this family of gene clusters (Fig. 2a, orange colored clades). While our screening suggests this is a common class of gene clusters in the environment, a search of all publicly available sequenced bacterial genomes only revealed one gene cluster that contains a similarly complex tailoring gene region and an AT8* domain. This previously uncharacterized gene cluster from *Amycolatopsis vancoresmycina* (*Ava*) is identical in gene content and organization to the RifCon 10 gene cluster that we recovered from a soil eDNA library (Fig. 3a).

### Highly functionalized congeners from an AT8* gene cluster

As an initial exploration of the tailoring gene-rich family of gene clusters that contain an AT8* domain, we looked for rifamycin congeners in ethyl acetate extracts from cultures of *Ava*. While *Ava* has never been reported to produce rifamycin-like metabolites, we identified three major HPLC peaks with rifamycin-like UV spectra in culture broth extracts (Supplementary Figure 4). The structure of each metabolite was elucidated using a combination of high-resolution mass spectrometry (HRMS), 1D and 2D NMR and UV data. ^13^C-NMR, HRESIMS [calcd *m/z* for C_50_H_64_NO_19_ (M + H^+^) 982.4073, found *m/z* 982.4025], and UV data for compound **1** were consistent with the structure of Kang A, a rifamycin congener originally characterized from *Amycolatopsis mediterranei* var. *kanglensis* and encoded by an uncharacterized gene cluster (Supplementary Figures 5–10, Supplementary Table 2)^22,23^. The most dramatic differences between Kang A and other rifamycins are that it contains a *β*-*O-*3,4^−^*O,O*’*-*methylene digitoxose deoxysugar (hereafter, K-sugar) substituent at C-27, an oxidized ethyl substituent in place of a methyl substituent at C-20, and a gem-dimethylsuccinic acid (K-acid) appended to the oxidized ethyl branch in the PK core (Fig. 3b).

The predicted molecular formula for the second metabolite, Kang V1 (**2**) [HRESIMS calcd *m/z* for C_50_H_65_NO_19_Na (M + Na^+^) 1006.4048, found *m/z* 1006.4006], suggested it was a reduced analogue of Kang A. A comparison of 1 and 2D NMR data from (**1**) and (**2**) allowed us to assign this difference to the reduction of the C-11 ketone to an alcohol [^13^C *δ* 77.1, ^1^H *δ* 5.49 (1 H, s)] (Supplementary Figures 1,1–1,6, Supplementary Table 2). To the best of our knowledge, this C-11 reduction has only been seen in one previously described rifamycin natural product congener, chaxamycin D^24^. In the case of the third metabolite, Kang V2 (**3**), HRMS data suggested it differed from Kang A (**1**) by the addition of a CH_2_ moiety [HRESIMS calcd *m/z* for C_51_H_66_NO_19_ (M + H^+^) 996.4229, found *m/z* 996.4197]. The UV spectra of Kang V2 (**3**) supported the presence of a naphthohydroquinone moiety (*λ*_max_ 302 nm) instead of the naphthoquinone (*λ*_max_ 276) seen in (**1**) and (**2**) (Supplementary Figure 4). The naphthohydroquinone substructure was also supported by an HMBC correlation from H-3 to a carbon at *δ* 150.5 ppm (C-4; Supplementary Figures 1,7–22, Supplementary Table 2). In Kang A and V1, this carbon is significantly more deshielded (*δ* 184.9 and 188.8 ppm, respectively). The presence of the carbonyl at C-8 in Kang V2 was supported by an HMBC correlation from the C-14 methyl to C-8 (*δ* 191.7). The formation of a fourth ring on the naphthohydroquinone substructure through the addition of a highly deshielded methylene [^13^C *δ* 98.4, ^1^H *δ* 6.19 (1 H, d), *δ* 5.48 (1 H, d)] was defined by HMBC correlations from the new methylene protons to C-4 and C-11 of the naphthohydroquinone (Supplementary Figure 1,7). To the best of our knowledge, the fourth ring formed by the addition of the methylenedioxy bridge in Kang V2 (**3**) is not found in any reported rifamycin congeners.

Many of the new structural features found on the Kangs can be rationalized based on differences in gene content between the Kang (*kng*) gene cluster and other rifamycin family gene clusters (Fig. 3a, c, Supplementary Figures 23 and 24, Supplementary Table 3). In addition to the AT8*-containing *kngD* domain, the *kng* cluster contains a collection of deoxysugar biosynthesis genes (*kng22*, *kng23*, and *kng27*) and a glycosyltransferase gene (*kng26*) that we predict are involved in generating the K-sugar modification^25,26^. The *kng* gene cluster also contains a set of genes (*kng30*, *kng34A/B*, and *kng35)* that we predict are involved in producing the K-acid; however, the genes responsible for installing the gem-dimethyl functionality on the succinic acid are not bioinformatically obvious. An *O*-methyltransferase, encoded by *kng24*, and an additional cytochrome P450, encoded by *kng28*, may participate in generating the methylenedioxy bridge found on the K-sugar as well as the Kang V2 ring system^27,28^.

### Kangs are active against Rif^R^ RNAPs **via** a distinct mechanism

Kangs A, V1 and V2 are active as antibiotics against Gram-positive bacteria, including *Staphylococcus aureus* (*Sau*), *Staphylococcus epidermidis*, *Listeria monocytogenes,* and *Mtb* (Supplementary Table 4). Kangs V1 and V2 both show improved activity against *Mtb* (H37Rv; IC_90_ 3.12 and 1.56 µM, respectively) compared to Kang A (12.5 µM). We were particularly interested in whether the complex structural features seen in the Kangs might impart improved activity against mutations in RNAP that confer Rif^R^. Substitutions at just three RNAP amino acid positions, *Mtb* RNAP β subunit D441, H451, and S456 (corresponding to *Msm*/*E. coli* [*Eco*] RNAP β subunit D432/D516, H442/H526, and S447/S531) account for the vast majority of mutations observed in Rif^R^ *Mtb* clinical isolates^3,29^. The antibacterial activity of the Kangs against Rif^R^ RNAP mutants was assessed in vivo using a collection of *Sau* strains carrying RNAP point mutations and in vitro using purified wild-type and Rif^R^ (S447L) *Msm* RNAPs^30,31^. The use of these models allowed us to explore the activity of the Kangs against mutations that correspond to the most commonly mutated sites in Rif^R^ *Mtb*, without necessitating the use of restrictive BSL3 assay conditions.

The Kangs are active against Rif^R^ *Sau* strains carrying RNAP mutations at sites corresponding to those commonly mutated in Rif^R^ *Mtb* clinical isolates (Fig. 3d). Kang V1 showed an ~80-fold lower MIC (0.069 µg mL^−1^) than Rif (5.6 µg mL^−1^) against a *Sau* RNAP βD471Y mutant strain. Kang V2 exhibited similarly potent activity (MIC 0.069 µg mL^−1^) against a *Sau* strain carrying an RNAP βS486L mutation, which corresponds to the most commonly observed Rif^R^ mutation in *Mtb* clinical isolates (*Mtb* RNAP βS456L), appearing in ~40–80% of sequenced isolates from geographically diverse regions of the world^32–40^. As with *Mtb*, the *Sau* RNAP βS486L mutation effectively abrogates antibacterial activity of Rif (MIC > 50 µg mL^−1^). Remarkably, Kang V2 showed more potent activity against the *Sau* RNAP βS486L mutant than against the wild-type strain, suggesting it might have evolved in a niche where this variant is the dominant form of RNAP. Based on the results of our MIC assay, we predicted that treatment of wild-type cells with Kangs V1 and V2 could effectively suppress the development of two common Rif^R^ phenotypes. Indeed, in *Sau* we were not able to identify any Kang V2 resistant mutants that carried the βS486L mutation (Supplementary Figure 25) nor could we identify any βD471Y mutants that arose when cultures were treated with Kang V1. Each of these mutations occurred at a frequency of approximately 10% among Rif^R^ *Sau* colonies. Consistent with the results of our MIC assay, an H481Y mutation, which confers a high level of resistance to all of the compounds, was the predominant mutation that arose following exposure of *Sau* to either Rif or the Kangs. While mutations at H481 were the most common variants we sequenced in Rif^R^ *Sau* strains (~70%), the βS456L mutation (*Sau* βS486L) predominates in Rif^R^ *Mtb* clinical isolates^29^.

To determine whether the activity of the Kangs against the *Sau* RNAP βS486L mutant could be generalized to mycobacterial RNAP carrying the equivalent mutation, we tested the in vitro activity of the Kangs against purified *Msm* RNAP using a run-off transcription assay (Fig. 3e). The *Msm* RNAP exhibits 91% sequence identity with *Mtb* RNAP at the amino acid level and shows complete conservation of residues in the Rif binding pocket^31^. We found that the Kangs were all potent in vitro inhibitors of wild-type *Msm* RNAP, with comparable activity to Rif. While Rif was inactive against an *Msm* RNAP βS447L mutant (corresponding to *Mtb*/*Sau* RNAP β S456L/S486L), all three Kangs displayed potent activity against this mutant. In agreement with the results of our *Sau* MIC assays, Kang V2 showed the highest potency against the Rif^R^ *Msm* RNAP (Fig. 3e).

### Kangs exhibit distinct mechanistic properties

Detailed analysis of the transcription assays suggested that the mechanism by which the Kangs inhibit RNAP differs from that of Rif. The effects of Rif on RNAP transcription activity at each stage of the transcription cycle have been probed extensively. Rif has little to no effect on promoter binding or open complex formation^41,42^, but causes an increase in the apparent *K*_*m*_ for the initiating substrate NTPs binding in the enzyme *i* and *i* *+* 1 sites^41,43^, thus affecting dinucleotide synthesis at lower NTP concentrations. Importantly, Rif does not affect RNAP catalysis itself (phosphodiester bond formation)^41,44^. The predominant effect of Rif is steric occlusion of the translocating nascent transcript after the formation of the first phosphodiester bond, resulting in the inhibition of the production of full-length transcript (F, Fig. 3e) but over-production of abortive dinucleotide transcripts (A, Fig. 3e)^41,44,45^. In contrast to the effect of Rif, the Kangs inhibited production of the full-length transcripts but also the abortive transcripts, suggesting that the Kangs inhibit a step of transcription preceding that of Rif—either substrate (DNA or initiating nucleotide) binding or phosphodiester bond catalysis itself.

### Kang A and Rif share core interactions with RNAP

While Kang V1 and V2 showed the highest levels of activity against bacteria carrying specific clinically important Rif^R^ mutations, all three Kangs exhibit improved activity compared to Rif against RNAP variants carrying common mutations found in Rif^R^ *Mtb* clinical isolates. We speculated that the activity of the Kangs against Rif^R^ mutants and their potentially novel mechanism of inhibition could be related to the presence of the unique K-sugar and K-acid, which all three Kangs share. To explore this hypothesis, we examined a crystal structure of a mycobacterial RNAP complexed with Kang A, the parent compound in the Kang family, and compared it to a structure complexed with Rif. A more detailed examination of the interaction between each Kang congener and the specific RNAP mutant against which it is most potent will be the focus of future studies.

**Fig. 4:**
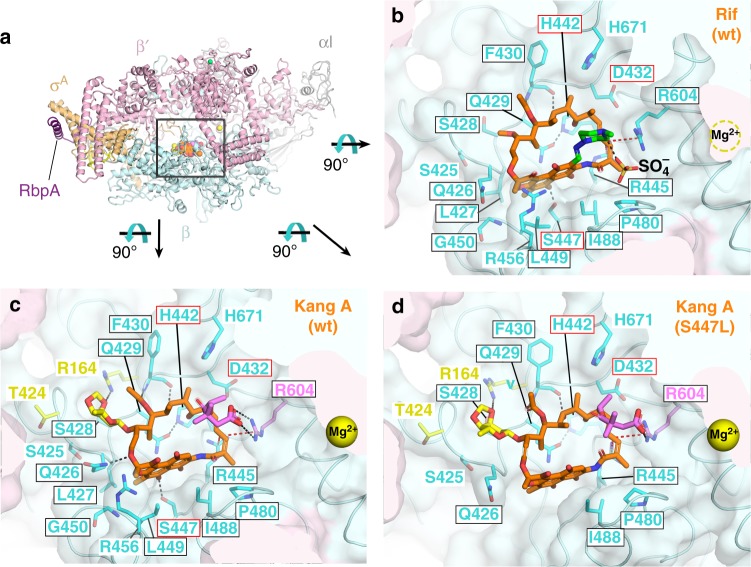
Structural basis for Kang A inhibition of Rif^R^ RNAP. **a** Overall view of the *Msm* TIC bound to Kang A. The Rif scaffold of Kang A is colored orange, K-sugar yellow, K-acid violet. wt, wild-type. The boxed region is magnified in (B) (showing Rif/wt-RNAP), (C) (Kang A/wt-RNAP), and (D) (Kang A/S447L-RNAP), but the view is rotated 90° as shown. **b** View into the Rif binding pocket (wt-RNAP) from inside the *Msm* RNAP active site cleft. Carbon atoms of the Rif scaffold are colored orange, carbon atoms of the 1-methyl-piperazine moiety are colored green. The RNAP is shown as a backbone worm with a transparent molecular surface (β, light cyan; β′, light pink). Density for the RNAP active site Mg^2+^ was very weak so it was not modeled, but its position is denoted by a dashed yellow circle. RNAP β subunit side chains that make direct contacts with Rif are shown, labeled and colored cyan. Polar interactions are indicated by dashed lines (gray, H-bonds; red, cation-π interactions). Strong electron density near the positively charged 1-methyl-piperazine group (Supplementary Figure S26) was interpreted as a SO_4_^−^ ion. Boxed residue labels denote residues that have been identified as conferring Rif^R^ when substituted^45^, with red boxes denoting three residues (*Msm* RNAP β D432, H442, and S447, corresponding to *Mtb* RNAP β D441, H451, and S456) that comprise the majority of Rif^R^ substitutions identified from clinical isolates from tuberculosis patients. **c**. View into the Kang A binding pocket (wt-RNAP). Kang A is colored as in (A). The RNAP is shown as in (A) except RNAP side chains that interact with K-sugar but not Rif (R164, T424) are colored yellow, and R604, which makes a salt bridge with K-acid is colored violet. Residues that confer Rif^R^ when substituted are denoted by colored boxes as in (B). **d** View into the Kang A binding pocket (S447L-RNAP). Kang A and RNAP are shown as in (C). The RNAP β subunit segment from 447–450 is distorted, and the loop from 451 to 465 is disordered, resulting in the loss of Kang A/RNAP contacts with β residues L427, L447, L449, G450, and R456

Kang A and Rif were soaked into crystals of an *Msm* RNAP transcription initiation complex (TIC)^31^. Both structures were phased by molecular replacement using the *Msm* TIC as a model and refined to 3.05 Å resolution (Fig. 4a–c, Supplementary Table 5). The structures of both antibiotics, including the K-sugar and K-acid moieties unique to the Kangs, as well as the RNAP β subunit interaction determinants for the antibiotics, were well-resolved (Supplementary Figure 26A and B). The tip of the σ-finger (a structural element of the σ subunit) also approaches each antibiotic and appears to make molecular contacts. However, because the σ-finger electron density is very weak (reflected in high atomic B-factors) and amino acid substitutions in σ that confer Rif^R^ have never been reported, the role of these interactions with Rif and Kang A remain to be established. We note that previous studies deleting the σ-finger suggested a role for this motif in binding to the Rif variant rifabutin but not to another variant, rifapentine^46^, indicating that the significance of σ-finger/antibiotic interactions is dependent on the specific Rif variant.

The Rif/*Msm* RNAP interactions were similar to those described in previous structures (Fig. 4b)^45–48^. The Rif/*Msm* RNAP structure reveals a set of cation-π interactions that have not been noted previously. The conjugated double-bond system comprising C16–C19 of the PK backbone of Rif is approached from the RNAP side by the guanidino group of R445 in a geometry indicative of a cation-π interaction^49^. The opposite face of the conjugated double-bond system is approached by the guanidino group of R604. We call this arrangement a cation-π sandwich (Supplementary Figure 26C). As expected, the Rif scaffold of Kang A binds in nearly the identical pocket and pose as Rif, and the interactions between the RNAP β subunit residues and the Kang A/Rif scaffold are nearly identical to Rif (Fig. 4c), including the cation-π sandwich (Supplementary Figure 26C and D).

### Structural basis of Kang inhibition of Rif^R^ RNAP

**Fig. 5:**
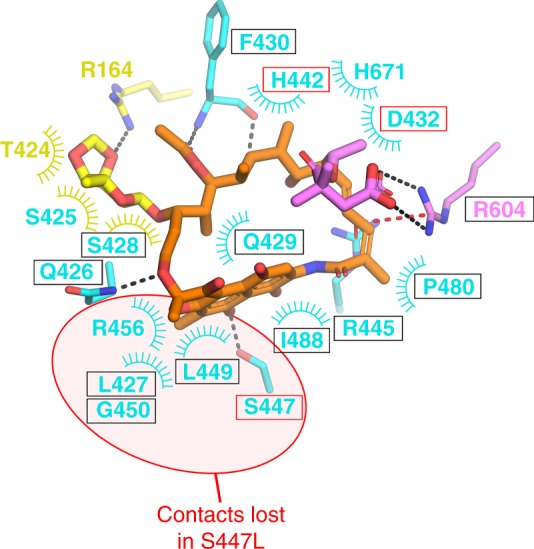
Kang A contacts with wild-type and Rif^R^ RNAP. Schematic summary of the Kang A/RNAP β subunit contacts. Residues that make only nonpolar contacts are shown as labels with arcs denoting the contacts. The side chains (or main chain atoms for F430) of residues that make polar contacts are shown in stick format (H-bonds, gray dashed lines; cation-π interactions, red dashed lines). The color-coding of residues/residue labels is as follows: residues that contact the Rif scaffold in the Rif/RNAP structure, cyan; residues that also make nonpolar contacts with K-sugar, yellow arc; residues that contact K-sugar but do not contact the Rif scaffold, yellow. R604 (colored violet) makes a cation-π interaction with the Rif scaffold but also makes a salt bridge with K-acid. Residues that confer Rif^R^ when substituted are denoted by colored boxes as in (A). Residues that lose contacts with Kang A in the Rif^R^ S447L RNAP mutant are denoted by red background shading

In addition to nearly identical interactions between the RNAP β subunit and the PK backbone of either Rif or Kang A, the chemical moieties unique to Kang A establish new interactions (Fig. 4b, c and Fig. 5). The K-sugar interacts with two β subunit residues that do not contact Rif, R164 and T424. These residues correspond to *Mtb*/*Eco* RNAP β R173/R143 and T433/S508, respectively. To our knowledge, neither of these residues has ever been identified as conferring Rif^R^ when substituted^45^.

The K-acid also establishes an interaction with RNAP that does not occur with Rif, a salt bridge (4.4 Å) with the guanidino group of β R604 (Fig. 4b, c). We believe this interaction stabilizes Kang A binding in two ways, first by forming a favorable salt bridge between the negatively charged K-acid and the positively charged R604, but in addition the salt bridge rigidifies the side chain of R604, which may stabilize the cation-π interaction with the Kang A PK backbone (Supplementary Figure 26D).

We propose that the additional interactions with RNAP contributed by the unique Kang moieties (K-sugar and K-acid) stabilize the binding of the Kangs sufficiently to overcome the loss of interactions caused by the S447L substitution, leading to an IC_90_ for the Kangs against this Rif^R^ RNAP that is at least two orders of magnitude lower than Rif (Fig. 3e). To test this hypothesis, we determined the structure of the Rif^R^ S447L RNAP in complex with Kang A and compared it to the structures of the wild-type enzyme bound to Rif and to Kang A. The structure was obtained similarly as described for the wild-type enzyme and was refined to 3.45 Å (Fig. 4d, Supplementary Table 5).

In the wild-type RNAP, S447(OG) forms a H-bond with Rif/Kang A(O2) (Fig. 4b, c) and this favorable interaction is lost with the S447L substitution (Fig. 4d and Fig. 5). In addition, substitution of the Ser by the bulkier, branched Leu residue has complex effects on the Rif binding pocket;^48^ the path of the polypeptide backbone is altered at L447 to accommodate the bulky substitution (Supplementary Figure 27), and as a consequence the β-subunit loop from residues 451-465 becomes disordered and nearby parts of the antibiotic binding pocket rearrange, resulting in the loss of nonpolar contacts between Kang A and L427, G450, L449, and R456. These structural changes do not affect other RNAP/antibiotic contacts including, importantly, contacts with K-sugar and K-acid (Fig. 4d and Fig. 5).

Binding of Rif to the wild-type RNAP results in a buried surface area of 2,880 Å^2^, while the binding of Kang A buries 3330 Å^2^. The additional chemical moieties of Kang A (K-sugar and K-acid) contribute about 450 Å^2^ of extra interaction area over Rif, and about 75% of that is contributed by the K-sugar. The binding of Kang A to the S447L RNAP results in a reduced buried surface area of 2940 Å^2^, a loss of 390 Å^2^ compared with Kang A/wild-type RNAP. Thus, the loss of 390 Å^2^ of buried surface area with Kang A due to the S447L substitution is more than compensated by the 450 Å^2^ of buried surface area gained from the K-sugar and K-acid interactions, supporting our hypothesis.

### Structural basis for the Kang mechanism of action

Rif inhibits RNAP function by blocking RNA translocation and extension after formation of the first or second phosphodiester bond^41,44,45^, resulting in inhibition of full-length transcript production along with an increase of abortive products (Fig. 3e). By contrast, the Kangs inhibit the production of both full-length and abortive products (Fig. 3e), indicating that the Kangs inhibit transcription at a step earlier than Rif.

We probed promoter DNA binding and loading of the template strand DNA into the RNAP active site, steps of transcription initiation preceding Rif inhibition, using DNase I footprinting (Supplementary Figure 28A) and RNAP active site directed Fe^2+^-mediated hydroxyl-radical cleavage (Supplementary Figure 28B). The results show that neither Rif nor Kang A significantly affect these steps, as observed previously for Rif^41^.

**Fig. 6:**
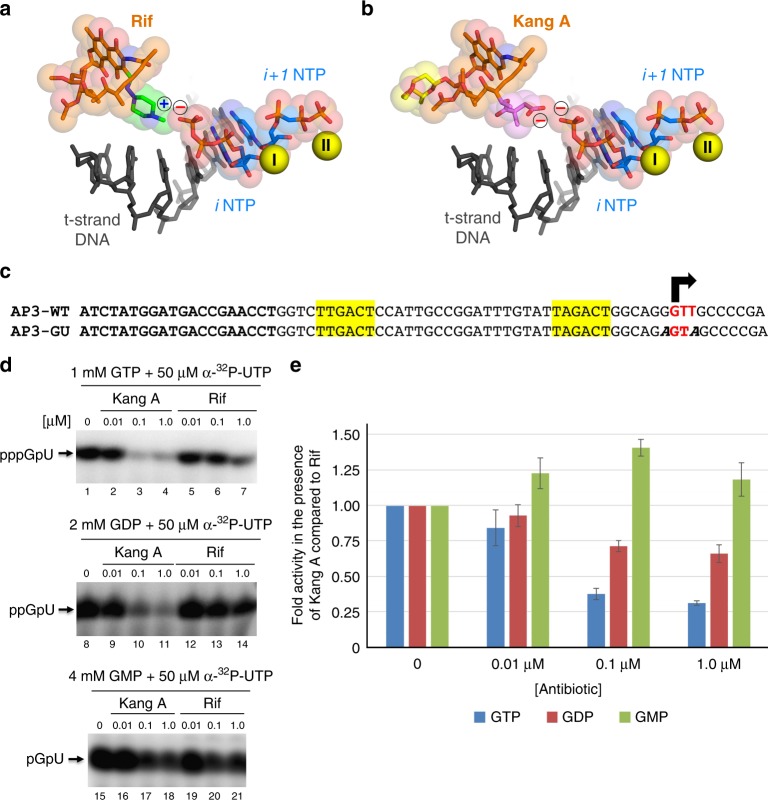
Structural basis for Kang A inhibition of *i*NTP binding. **a** View of the RNAP active site from the *T. thermophilus* de novo initiation complex (4Q4Z)^50^ with bound Rif superimposed. Shown is the t-strand DNA from +1 to −5 (dark gray), the initiating NTP substrates (*i* site NTP, ATP; *i* *+* 1 NTP, CMPCPP; blue carbon atoms) and two Mg^2+^-ions (yellow spheres; Mg^2+^I is the Mg^2+^-ion chelated in the RNAP active site, Mg^2+^II is bound to the *i* *+* 1 NTP). Rif is color-coded as in Fig. 4b. Rif and the NTPs are also shown with transparent van der Waals surfaces. The blue “+” denotes the positive charge of the Rif piperazine moiety, while the red “−” denotes the negative charge of the *i*NTP γ-phosphate. **b** Same as (A) but showing Kang A (colored as in Fig. 4c). The negative charge of K-acid is brought in close proximity to the negative charge of the *i*NTP γ-phosphate. **c** Sequence of AP3-GU promoter template used in in vitro abortive initiation assays monitoring the effect of Kang A or Rif on RNA dinucleotide synthesis with GTP, GDP, or GMP as the 5′-initiating nucleotide. The initial transcribed sequence of the *Mtb* AP3 promoter (top) was engineered to allow only RNA dinucleotide synthesis (5′-GU-3’) in the presence of GTP, GDP, or GMP as the 5′-initiating nucleotide and UTP. The mutated bases are denoted in bold italic (AP3-GU, bottom). **d** Kang A or Rif inhibition of in vitro abortive initiation of RNA dinucleotide synthesis using the AP3-GU promoter template; (top) 1 mM GTP + 50 μM α-^32^P-UTP; (middle) 2 mM GDP + 50 μM α-^32^P-UTP; (bottom) 4 mM GMP + 50 μM α-^32^P-UTP. **e** Plotted is the RNA dinucleotide synthesis with Kang A relative to the same condition with Rif, normalized by the results with no antibiotic. Kang A has a strong inhibitory effect with GTP as the 5′-initiating nucleotide (blue bars), a weaker effect with GDP (red bars), and no inhibitory effect with GMP (green bars). The error bars denote standard error of four measurements

We next investigated substrate binding and phosphodiester bond formation. We modeled the positions of the first two nucleotide substrates occupying the *i* and *i* *+* 1 sites (the 5′- and 3′-initiating nucleotides, respectively) in an initiating complex by superimposing the structure of a *T. thermophilus* RNAP de novo initiation complex (4Q4Z)^50^ onto the *Msm* RNAP/Rif and Kang A structures (Fig. 6a, b). Rif did not clash sterically with the DNA or the NTP substrates, consistent with findings that Rif has only very small effects on the *K*_*m*_ for initiating substrate^41^. The Rif piperazine moiety approaches the γ-phosphate of the modeled *i* site nucleotide (*i*NTP), and because the Rif piperazine N4 is positively charged and is poised within 3.6 Å from the closest oxygen in the modeled (*i*NTP) γ-phosphate, this interaction would not disfavor iNTP binding.

In the modeled de novo initiation complex with Kang A, the pose of Kang A positioned the negatively charged carboxylic group of the K-acid very close (2.5 Å between the closest oxygen of each group) to the negatively charged *i*NTP γ-phosphate (Fig. 6b), suggesting that Kang A may increase the *K*_*m*_ of the *i*NTP by Coulombic repulsion. To test this hypothesis, we took advantage of RNAPs ability to efficiently initiate de novo with an NDP (β-phosphate 6.5 Å from K-acid) or an NMP (α-phosphate 8.0 Å from K-acid) as the 5′-initiating substrate (*K*_*m*_^*iNTP*^ ~*K*_*m*_^*iNDP*^ ~1 mM; *K*_*m*_^*iNMP*^ ~5 mM)^41,51^. To monitor only RNA dinucleotide synthesis, we used a mutant duplex *Mtb* AP3 promoter template (AP3-GU; Fig. 6c) in which the initial transcribed sequence was engineered to ensure only RNA dinucleotide synthesis, either pppGpU, ppGpU,or pGpU, in the presence of α-P^32^-UTP (0.3 μM) and either 1 mM GTP, 2 mM GDP, or 4 mM GMP (Fig. 6d). As expected, Rif has an inhibitory effect on dinucleotide synthesis (Supplementary Figure 28C)^41,43^. However, relative to Rif, Kang A has a strong inhibitory effect on RNA dinucleotide synthesis when GTP serves as the 5′-initiating nucleotide, a weaker inhibitory effect with GDP, and no inhibitory effect with GMP (Fig. 6e). These results strongly support the hypothesis that Kang A interferes with binding of the *i*NTP substrate via Coulombic repulsion between the K-acid and the *i*NTP γ-phosphate (Fig. 6).

Note that this mechanism for Kang A inhibition of initial phosphodiester bond formation does not preclude inhibition of RNA chain elongation by steric occlusion, the mechanism of action for Rif^41,44,45^. Maximal inhibition of pppGpU synthesis (at 1 μM antibiotic) by Kang A is about 75% (Fig. 6d), while inhibition of full-length transcripts in the run-off assay at the same Kang A concentration is essentially 100% (Fig. 3e), indicating that Kang A inhibits RNA chain synthesis via two mechanisms, inhibition of initial phosphodiester bond formation by interfering with binding of the *i*NTP substrate, and blocking RNA chain elongation subsequent to formation of the first phosphodiester bond, the latter mechanism being in common with Rif.

## Discussion

**Fig. 7:**
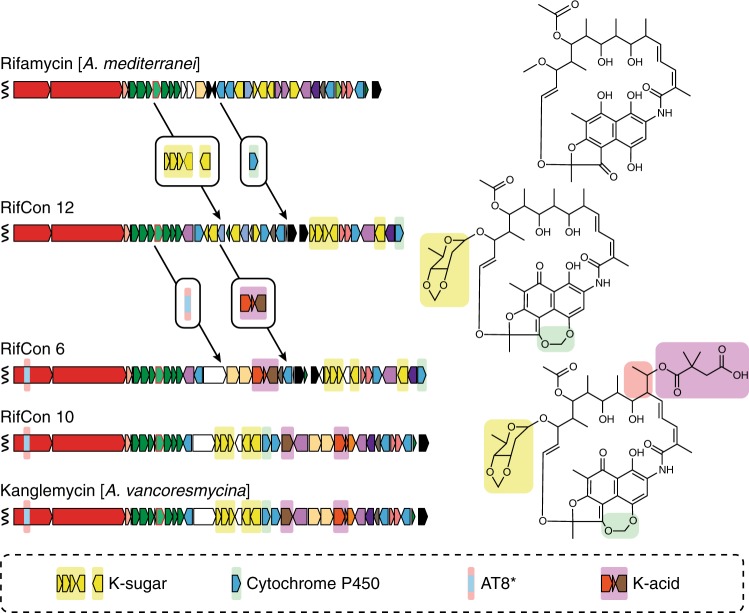
Model for the evolution of a structurally complex Kang family molecule. A stepwise increase in the structural complexity of the antibiotic is envisioned to result from a series of horizontal gene transfer events. Genes acquired at each step are shown in boxes and are highlighted according to the structural feature they are predicted to encode

The potent activity of the Kangs against common mutations that confer Rif^R^ in clinical settings suggests that they may have arisen in response to prevalent resistance phenotypes in the producing bacterium’s natural environment. An examination of gene clusters we recovered from soil metagenomes provides potential insight into how these molecules could have evolved from an ancestral rifamycin-like gene cluster through a series of horizontal gene transfer events. The simplest *kng* related gene cluster we identified (RifCon 12) contains additional biosynthetic genes, not seen in other rifamycin congener gene clusters, that we believe are required for the biosynthesis and transfer of the K-sugar (Fig. 7, genes highlighted in yellow) as well as the formation of the methylenedioxy bridge on the naphthohydroquinone seen in Kang V2 (Fig. 7, gene highlighted in green). This gene cluster does not, however, contain any genes predicted to encode for the incorporation of either the Emal or K-acid moieties seen in the Kangs (Supplementary Table 6). It is possible that this simpler gene cluster arose from a *kng*-like gene cluster through a series of gene deletion events, but we believe it is more likely that it represents an ancestor of a *kng*-like gene cluster. The subsequent acquisition of genes that encode for the Emal and K-acid modifications (Fig. 7, genes highlighted in red and purple, respectively) would enable the biosynthesis of a fully functionalized Kang. In total, we identified three complete *kng*-like gene clusters—two from soil metagenomes (RifCon 6 and RifCon 10) and one from a cultured bacterium. Each is predicted to contain a full complement of *kng* biosynthetic genes differing only by sequence, gene organization and accessory gene content (pumps, transcription factors, precursor biosynthetic genes, etc.) (Fig. 7, Supplementary Tables 7 and 8).

In our model, the different stages for the evolution of the Kangs from a simpler rifamycin-like molecule can be rationalized with respect to our structural and mechanistic studies. The initial acquisition of the K-sugar likely proved advantageous in providing additional contacts with RNAP that stabilize the binding of the Kangs in the presence of destabilizing Rif^R^ mutations. The later acquisition of the dimethylsuccinic acid functionality would have added a second mechanism of action via the inhibition of initiating substrate binding, in addition to the steric occlusion mechanism shared with Rif. Rif binding/inhibition is competitive with RNA transcripts longer than about 3 nucleotides^41,45^. The ability of RNAP to continuously synthesize 2–3 nucleotide abortive products in the presence of Rif (RNAP priming) likely increases the probability of producing a longer transcript if Rif transiently dissociates within the lifetime of the RNAP/promoter complex, which would block rebinding of Rif. Kang inhibition of initiating substrate binding, mediated by the K-acid, could minimize this priming mechanism. Thus, the novel inhibition mechanism may serve to further increase the potency of the Kangs, especially in the context of Rif^R^ mutations that decrease the lifetime of the antibiotic-bound state.

Efforts to improve rifamycin through semisynthesis have been most productive when focusing on modifications of the naphthohydroquinone^52–54^. Modification of this substructure has yielded the clinically used drugs rifampicin, rifapentine, rifabutin and rifaximin. Interestingly, in the case of the Kangs, evolution has led to the creation of biologically interesting congeners modified at three different positions, all of which have either been largely inaccessible or unproductive in semisynthesis studies.

While we do not know for certain that the evolution of the Kangs provides a selective advantage to the producing organisms in an ecological niche populated by Rif^R^ bacteria, their activity against this phenotype suggests this is likely to be the case. Competition between environmental bacteria may have provided strong evolutionary pressure to evolve antibiotic variants that are capable of circumventing common resistance mechanisms, including those that are prevalent in clinical settings. Large-scale metagenome sequencing methods, like those used here, allow for the systematic identification of the most complex gene clusters in known antibiotic families, which may represent highly evolved natural solutions to commonly encountered antibiotic resistance mechanisms. If this proves true across other families of gene clusters that encode antibiotics, a systematic examination of the global microbiome for new congeners of antibiotics in clinical use would likely uncover additional natural products capable of circumventing common clinically important antibiotic resistance mechanisms.

## Methods

### Screening soil for AHBA synthase gene sequences

eDNA was extracted from each soil sample using a modified DNA extraction protocol^55,56^. Briefly, approximately 25 g of each soil was placed in a 50 mL falcon tube. 30 mL of lysis buffer (100 mM Tris-HCl, 100 mM ethylenediaminetetraacetic acid (EDTA), 1.5 M NaCl, 1% (w/v) cetyltrimethylammonium bromide, 2% (w/v) sodium dodecyl sulfate, pH 8.0) were added to each tube. After a 2-h incubation at 70 °C with gentle mixing by inversion in 15 min intervals, the tubes were spun down at 5000×*g* for 10 min at 4 °C. The supernatant was decanted into a clean tube and 0.6 volumes of isopropanol were added to precipitate DNA. Precipitated DNA was pelleted by centrifugation at 5000×*g* for 30 min at 4 °C. The pellet was washed with 70% ethanol and allowed to air-dry for several hours at room temperature. The dried DNA pellet was resuspended in 500 μL TE (10 mM Tris-HCl, 1 mM EDTA, pH 8.0). The resulting crude eDNA samples were screened with degenerate primers targeting the AHBA synthase gene, *rifK*: (forward) 5′-CCSGCCTTCACCTTCATCTCCTC-3′ and (reverse) 5′-AYCCGGAACATSGCCATGTAGTG-3′^15^. These degenerate primers were appended with a collection of distinct 8 bp barcodes and 1–4 bp spacer sequences^57^ that were used to distinguish amplicons generated from each soil. All primers were also appended with adapters for Illumina sequencing: 5′-CTACACGACGCTTTCCGATCT-3′ (forward primer adapter); 5′-CAGACGTGTGCTCTTCCGATCT-3′ (reverse primer adapter). A typical eDNA PCR reaction contained 1 μL Thermopol master mix (10X stock, New England BioLabs Inc.), 0.1 μL rTaq polymerase (5 units ul^−1^ stock; Bulldog Bio), 0.5 μL of each primer (10 μM stock concentration), 2 μL of eDNA and 5.9 μL of water. A touchdown PCR protocol was used for all screening: 5 min at 95 °C, followed by 6 cycles of, 30 sec at 95 °C, 30 sec at 65 °C (−1 °C/cycle) and 40 s at 72 °C, followed by 29 cycles of 30 s at 95 °C, 30 s at 58 °C and 40 s at 72 °C. PCR reactions were pooled and size selected by electrophoresis using an E-Gel (Invitrogen) prior to sequencing.

### Sequencing of AHBA synthase gene amplicons

Sequencing of pooled amplicons was performed by Illumina MiSeq using 300 bp paired-end reads. The forward reads were trimmed to 240 bp, the reverse reads were trimmed to 175 bp. The mate-paired reads were concatenated and subsequently clustered at 90% within each soil sample using USEARCH v7^58^. Single reads were removed and the centroid amplicon sequences were reclustered at 97% identity across soils. Reference AHBA synthase sequences from published gene clusters were trimmed to the same 240 bp at the 5′ end and 175 bp from the 3′ end as the sequencing reads and combined with the reclustered centroid amplicon sequences. The combined sequences were aligned with MUSCLE v3.8.31^59^ and a phylogenetic tree was constructed using FastTree v2.1.10^60^. The phylogenetic tree of AHBA amplicons from crude soils and reference sequences was examined for sub-clades containing sequences more closely related to rifamycin AHBA synthase sequences than to AHBA synthase genes from other ansamycin gene clusters.

### Library construction and screening for AHBA synthase genes

For construction of each metagenomic library, 500 g of soil was sifted to remove large particulate matter and heated to 70 °C for 2 h in lysis buffer [100 mM Tris-HCl, 100 mM EDTA, 1.5 M NaCl, 1% (w/v) cetyltrimethylammonium bromide, 2% (w/v) SDS, pH 8.0]^56^. The crude lysate was then centrifuged to remove additional soil particulates. eDNA was precipitated from the supernatant using 0.7 volumes of isopropanol, collected by centrifugation and washed with 70% ethanol before being resuspended in TE. High-molecular weight eDNA was gel purified, blunt ended, and ligated into pWEB::TNC (Epicenter). The ligation products were then packaged into lambda phage and transfected into *Eco* EC100 (Lucigen). Each newly constructed metagenomic library contained 10-60 million individual cosmid clones with ~30–45 kb eDNA inserts. Each library was constructed as 768 subpools (2 × 384 wells) containing 25–60 thousand unique cosmid clones per pool. Subpools were stored both as glycerol stocks to facilitate the recovery of individual cosmids of interest and as purified cosmid DNA to facilitate PCR-based screening. To identify subpools from which AHBA synthase containing clones could be recovered, cosmid DNA from each pool served as template in PCR reactions with the same degenerate primers that were used to screen eDNA. The resulting PCR amplicons were gel purified and Sanger sequenced to identify subpools with ABHA synthase gene containing cosmids. Cosmid clones containing AHBA synthase genes were recovered from ABHA synthase amplicon positive sublibrary pools by dilution of the pools and tracking of the target clones by PCR^61^. Cosmids were sequenced using ion PGM technology and reads were assembled into contigs using Newbler^62^. All contigs were analyzed using open reading frame (ORF) predictions from MetaGeneMark^63^ and BLAST^64^. Putative functions for new tailoring enzymes were assigned based on the predicted function of close relatives identified by Blast searches.

### Recovery of gene clusters and analysis of tailoring genes

To recover overlapping cosmids containing PKS and tailoring regions associated with the recovered AHBA synthase genes, DNA from library subpools was screened with degenerate primers targeting two additional conserved sequences in rifamycin biosynthesis: ketosynthase (KS) domains and post-PKS tailoring genes *rif15A/15B*. In known rifamycin congener gene clusters the PKS region resides directly upstream (5′) of the AHBA biosynthesis operon. The *rif15A/15B* genes, which are predicted to encode subunits of a transketolase are generally found at the very downstream (3′) edge of the tailoring region of a rifamycin congener gene cluster. The primers used for targeting the KS domains were: 5′-ATCGAGGCSCAGGCSYTG-3′ (forward) and 5′-GAYSASGTGSGCGTTSGT-3′ (reverse). These primers were appended with adapters for Ion Torrent Personal Genome Machine (PGM) System sequencing: 5′-CCATCTCATCCCTGCGTGTCTCCGACTCAG-3′ (forward primer adapter) and 5′-CCTCTCTATGGGCAGTCGGTGAT-3′ (reverse primer adapter). The primers used for targeting the *rif15A/B* genes were: 5′-CCGGTTCTAYCTSTCCAAG-3′ (forward) and 5′-AASRACCACGASGAGATGT-3′ (reverse). These primers were appended with the same Illumina adapters that were used with AHBA synthase primers. As with the AHBA synthase primers, each set of KS and *rif15A/15B* degenerate primers was also appended with well-specific 8 bp barcodes^57^. The same PCR conditions used for AHBA synthase screening were used for KS and *rif15A/15B* screening. Amplicons were sequenced using Ion Torrent PGM (KS amplicons) or Illumina MiSeq (*rif15A/15B* amplicons) technologies. The 300 bp paired-end reads were processed as described for AHBA synthase amplicons. Tracking the co-localization of rifamycin-like KS, AHBA synthase and *rif15A/15B* sequences across library subpools allowed us to identify clones that overlapped with the AHBA synthase containing clones we initially sequenced and thereby recover sets of overlapping cosmids that comprise complete biosynthetic gene clusters. Overlapping sequences were then assembled into larger contigs to create full gene clusters. For phylogenetic analyses of predicted tailoring genes, genes were extracted from all sequenced eDNA tailoring regions as well as tailoring regions from rifamycin gene clusters found in GenBank. Tailoring genes were grouped according to predicted functional class (glycosyltransferase genes, cytochrome P450 genes, etc.), and aligned with MUSCLE v3.8.31^59^. A phylogenetic tree was constructed for each functional class using FastTree v2.1.10^60^.

### *Ava* fermentation

A spore stock of *Ava* (NRRL B-24208) was created from cultures grown on MS plates (20 g L^−1^ mannitol, 20 g L^−1^ soya flour, and 20 g L^−1^ agar)^65^. Spores were stored frozen at −20 °C in 20% glycerol. For metabolite production, 5 μL of a glycerol spore stock was used to inoculate 50 mL TSB media (Oxoid) in 125 mL baffled Erlenmeyer flasks, which were shaken at 30 °C and 230 rpm. The following day, 200 μL of the overnight TSB starter culture was used to inoculate 50 mL of R5A media (100 g L^−1^ sucrose, 0.25 g L^−1^ K_2_SO_4_, 10.12 g L^−1^ MgCl_2_·6H_2_0, 10 g L^−1^ glucose, 0.1 g L^−1^ casamino acids, 20.5 g L^−1^ MOPS, 5 g L^−1^ yeast extract, and 2 g L^−1^ NaOH)^66^ in 125 mL baffled Erlenmeyer flasks. As the metabolite production profile and yield were found to respond favorably to increased aeration, a 1′′ × 1′′ stainless steel metal mesh was added to each flask. Flasks were grown for 6 days at 30 °C and 200 rpm.

### Isolation of Kangs A, V1, and V2

After 6 days of shaking, flasks were combined and extracted using a 2:1 ratio of neutral ethyl acetate to fermentation broth. The resulting crude extract was fractionated by flash chromatography (RediSep Rf, High Performance Gold 50 g HP C18 resin) using a linear gradient of 30–100% acetonitrile:water with 0.1% acetic acid over 30 min. The column elution was monitored by UV and fractions containing a strong absorbance at both 254 nm and 420 nm were pooled. Pooled fractions were diluted with four volumes of H_2_O and loaded onto a solid phase extraction column (Grace Biosciences). After binding and washing with H_2_O, the column was eluted with methanol. The Kangs were then purified by HPLC using a 10 mm × 150 mm C_18_ column (Waters) and an isocratic method of 46% acetonitrile with 0.1% formic acid at a flow rate of 3.14 mL min^−1^. The compounds eluted with retention times of 38 min (Kang A), 43 min (Kang V2) and 50 min (Kang V1). Typical yields of purified Kang A, V1 and V2 were 0.3 mg, 0.4 mg and 0.1 mg, respectively, per liter of culture.

### Structure elucidation of Kangs A, V1, and V2

NMR studies for Kang A and V1 were performed at room temperature on a Bruker Avance 600 MHz NMR equipped with a TCI triple resonance cryoprobe. Spectra for Kang A and V1 were collected in methylene chloride and deuterated methanol, respectively. Kang V2 exhibited broad peaks in all common NMR solvents when data were collected at room temperature. The peaks were considerably sharper at lower temperatures. Our final dataset for Kang V2 was collected in deuterated methanol at −20 °C on a Bruker AvanceHD 500 MHz NMR equipped with a TCI cryoprobe with enhanced H^1^/F^19^ and C^13^ detection and low temperature (−40 °C) capabilities (Weill Cornell Medicine NMR Core). HRMS analysis was performed at Memorial Sloan-Kettering Cancer Center using an LCT Premier XE system (Waters).

Kang A. ^1^H NMR (600 MHz, CD_2_Cl_2_): *δ* 12.60 (s, 1 H, OH-8), 8.34 (s, 1 H, NH), 7.80 (s, 1 H, H-3), 6.37 (d, *J* *=* 12.8 Hz, 1 H, H-29), 6.16 (d, *J* *=* 5.8 Hz, 1 H, H-17), 5.93 (dd, *J* *=* 15.9 Hz, 5.8 Hz, 1 H, H-18), 5.81 (dd, *J* *=* 15.9 Hz, 9.3 Hz, 1 H, H-19), 5.13 (dd *J* *=* 12.8 Hz, 9.3 Hz, 1 H, H-28), 5.13 (s, 1 H, H-K12), 5.06 (dd, *J* = 12.7 Hz, 6.3 Hz, 1 H, H-K2), 4.87 (s, 1 H, H-K12), 4.65 (dd, *J* *=* 9.0 Hz, 1.1 Hz, 1 H, H-K9), 4.36 (dd, *J* *=* 9.5 Hz, 1.0 Hz, 1 H, H-25), 4.10 (m, 1 H, H-K11), 3.85 (dd, *J* *=* 9.3 Hz, 2.7 Hz, 1 H, H-27), 3.68 (m, 1 H, H-21), 3.64 (m, 1 H, H-K13), 3.36 (m, 1 H, H-K14), 2.85 (dd, *J* *=* 10.0 Hz, 1.8 Hz, 1 H, H-23), 2.66 (d, *J* *=* 16.9 Hz, 1 H, H-K7), 2.53 (d, *J* *=* 16.9 Hz, 1 H, H-K7), 2.34 (s, 3 H, H-14), 2.23 (ddd, *J* *=* 15.5 Hz, 2.9 Hz, 1.1 Hz, 1 H, H-K10), 2.15 (m, 1 H, H-20), 2.13 (m, 1 H, H-26), 2.06 (s, 3 H, H-30), 2.02 (s, 3 H, H-36), 1.84 (m, 1 H, H-22), 1.83 (m, 1 H, H-K10), 1.67 (s, 3 H, H-13), 1.60 (m, 1 H, H-24), 1.27 (d, *J* *=* 6.2 Hz, 3 H, H-K15), 1.23 (s, 3 H, H-K8), 1.17 (s, 3 H, H-K5), 1.07 (d, *J* *=* 6.3 Hz, 3 H, H-K1), 0.94 (d, *J* *=* 7.0 Hz, 3 H, H-32), 0.69 (d, *J* *=* 6.7 Hz, 3 H, H-33), 0.38 (d, *J* *=* 7.2 Hz, 3 H, H-34); ^13^C NMR (150 MHz, CD_2_Cl_2_): *δ* 194.1 (C-11), 185.8 (C-1), 184.9 (C-4), 176.4 (C-K3), 174.1 (C-35), 172.3 (C-K6), 171.8 (C-6), 171.6 (C-15), 167.4 (C-8), 146.4 (C-29), 140.9 (C-2), 137.0 (C-16), 134.1 (C-19), 132.0 (C-10), 129.8 (C-17), 128.2 (C-18), 117.0 (C-3), 116.9 (C-7), 112.9 (C-28), 111.7 (C-5), 111.2 (C-9), 109.9 (C-12), 97.2 (C-K9), 95.4 (C-K12), 81.5 (C-27), 79.0 (C-23), 75.9 (C-K13), 74.9 (C-K11), 74.1 (C-25), 70.5 (C-K14), 68.9 (C-21), 68.0 (C-K2), 53.0 (C-20), 43.5 (C-K7), 40.8 (C-K4), 37.0 (C-26), 36.9 (C-24), 33.8 (C-22), 33.5 (C-K10), 26.2 (C-K5), 24.9 (C-K8), 23.7 (C-13), 21.8 (C-36), 21.1 (C-30), 19.8 (C-K1), 18.8 (C-K15), 13.4 (C-34), 12.9 (C-32), 9.4 (C-33), 7.8 (C-14); HRMS (*m*/*z*): (M + H^+^) calcd. for C_50_H_64_NO_19_, 982.4073, found, 982.4025.

Kang V1. ^1^H NMR (600 MHz, CD_3_OD): *δ* 7.69 (s, 1 H, H-3), 6.41 (dd, *J* *=* 15.4 Hz, 11.3 Hz, 1 H, H-18), 6.33 (d, *J* *=* 11.3 Hz, 1 H, H-17), 6.15 (d, *J* *=* 12.3 Hz, 1 H, H-29), 5.98 (dd, *J* *=* 15.4 Hz, 8.4 Hz, 1 H, H-19), 5.49 (s, 1 H, H-11), 5.16 (dd, *J* *=* 12.3 Hz, 4.0 Hz, 1 H, H-28), 5.11 (dd, *J* *=* 10.5 Hz, 1.6 Hz, 1 H, H-25), 5.06 (m, 1 H, H-K2), 5.06 (s, 1 H, H-K12), 4.79 (s, 1 H, H-K12), 4.52 (dd, *J* *=* 8.0 Hz, 2.3 Hz, 1 H, H-K9), 4.06 (dd, *J* *=* 7.6 Hz, 3.9 Hz, 1 H, H-21), 4.02 (m, 1 H, H-K11), 3.90 (m, 1 H, H-27), 3.55 (dd, *J* *=* 9.0 Hz, 5.5 Hz, 1 H, H-K13), 3.31 (signal overlapped with solvent peak, H-K14), 3.07 (dd, *J* *=* 11.1 Hz, 1.8 Hz, 1 H, H-23), 2.75 (d, *J* *=* 16.5 Hz, 1 H, H-K7), 2.57 (d, *J* *=* 16.5 Hz, 1 H, H-K7), 2.33 (m, 1 H, H-20), 2.20 (s, 3 H, H-14), 2.10 (ddd, *J* *=* 13.5 Hz, 8.0 Hz, 4.8 Hz, 1 H, H-K10), 2.07 (s, 3 H, H-30), 2.03 (s, 3 H, H-36), 1.86 (s, 3 H, H-13), 1.86 (m, 1 H, H-22), 1.74 (m, 1 H, H-K10), 1.64 (m, 1 H, H-24), 1.40 (m, 1 H, H-26), 1.27 (s, 3 H, H-K5), 1.27 (s, 3 H, H-K8), 1.20 (d, *J* *=* 6.3 Hz, 3 H, H-K15), 1.19 (d, *J* *=* 6.9 Hz, 3 H, H-K1), 1.01 (d, *J* *=* 7.1 Hz, 3 H, H-32), 0.60 (d, *J* *=* 6.8 Hz, 3 H, H-33), 0.22 (d, *J* *=* 7.0 Hz, 3 H, H-34); ^13^C NMR (150 MHz, CD_3_OD): *δ* 188.8 (C-4), 183.8 (C-1), 177.9 (C-K3), 174.6 (C-K6), 173.4 (C-35), 172.0 (C-15), 165.8 (C-8), 164.9 (C-6), 143.3 (C-29), 142.9 (C-2), 134.4 (C-19), 133.6 (C-16), 133.0 (C-17), 130.8 (C-18), 127.1 (C-10), 126.3 (C-9), 118.4 (C-28), 118.2 (C-3), 114.3 (C-7), 113.9 (C-12), 109.4 (C-5), 101.0 (C-K9), 95.7 (C-K12), 78.3 (C-23), 78.0 (C-27), 77.1 (C-11), 77.0 (C-K13), 75.2 (C-K11), 74.9 (C-25), 71.2 (C-K14), 71.1 (C-K2), 70.2 (C-21), 51.6 (C-20), 44.9 (C-K7), 41.8 (C-K4), 41.2 (C-26), 38.4 (C-24), 35.3 (C-22), 33.6 (C-K10), 26.6 (C-K5), 25.3 (C-K8), 25.0 (C-13), 20.9 (C-36), 20.2 (C-30), 18.9 (C-K15), 18.8 (C-K1), 13.5 (C-32), 10.3 (C-34), 10.1 (C-33), 8.4 (C-14); HRMS (*m*/*z*): (M + Na^+^) calcd. for C_50_H_65_NO_19_Na, 1006.4048, found, 1006.4006.

Kang V2. ^1^H NMR (500 MHz, CD_3_OD): *δ* 7.99 (s, 1 H, H-3), 6.52 (dd, *J* *=* 15.6 Hz, 11.2 Hz, 1 H, H-18), 6.30 (d *J* *=* 11.2 Hz, 1 H, H-17), 6.22 (d *J* *=* 12.5 Hz, 1 H, H-29), 6.19 (d, *J* *=* 6.6 Hz, 1 H, H-K16), 5.94 (dd, *J* *=* 15.6 Hz, 8.1 Hz, 1 H, H-19), 5.48 (d, *J* *=* 6.6 Hz, 1 H, H-K16), 5.36 (dd, *J* *=* 12.5 Hz, 5.5 Hz, 1 H, H-28), 5.12 (dd, *J* *=* 10.3 Hz, 1.7 Hz, 1 H, H-25), 5.06 (s, 1 H, H-K12), 5.02 (dd, *J* *=* 5.9 Hz, 4.1 Hz, 1 H, H-K2), 4.76 (s, 1 H, H-K12), 4.48 (dd, *J* *=* 7.8 Hz, 1.4 Hz, 1 H, H-K9), 4.06 (m, 1 H, H-21), 3.99 (m, 1 H, H-K11), 3.92 (dd, *J* *=* 5.5 Hz, 2.0 Hz, 1 H, H-27), 3.54 (dd, *J* *=* 9.1 Hz, 5.3 Hz, 1 H, H-K13), 3.30 (signal overlapped with solvent peak, H-K14), 3.06 (dd, *J* *=* 10.2 Hz, 1.8 Hz, 1 H, H-23), 2.69 (d, *J* *=* 16.9 Hz, 1 H, H-K7), 2.56 (d, *J* *=* 16.9 Hz, 1 H, H-K7), 2.33 (m, 1 H, H-20), 2.13 (m, 1 H, H-K10), 2.08 (s, 3 H, H-30), 2.06 (s, 3 H, H-36), 2.01 (s, 3 H, H-14), 1.86 (s, 3 H, H-13), 1.81 (m, 1 H, H-22), 1.80 (m, 1 H, H-K10), 1.43 (m, 1 H, H24), 1.31 (s, 3 H, H-K8), 1.29 (s, 3 H, H-K5), 1.20 (m, 1 H, H-26), 1.18 (d, *J* *=* 7.1 Hz, 3 H, H-K1), 1.18 (d, *J* *=* 6.2 Hz, 3 H, H-K15), 0.99 (d, *J* *=* 6.7 Hz, 3 H, H-32), 0.51 (br s, 3 H, H-33), -0.06 (d *J* *=* 5.6 Hz, 3 H, H-34); ^13^C NMR (125 MHz, CD_3_OD): *δ* 191.7 (C-8), 177.9 (C-K3), 174.8 (C-K6), 172.7 (C-35), 171.7 (C-15), 167.9 (C-6), 165.7 (C-11), 150.5 (C-4), 150.4 (C-1), 141.8 (C-29), 134.7 (C-16), 132.5 (C-19), 132.0 (C-17), 131.3 (C-18), 126.4 (C-2), 123.3 (C-28), 118.2 (C-3), 115.4 (C-10), 114.1 (C-9), 112.9 (C-12), 108.3 (C-7), 106.4 (C-5), 101.0 (C-K9), 98.4 (C-K16), 95.7 (C-K12), 78.2 (C-27), 77.9 (C-23), 76.7 (C-K13), 75.2 (C-K11), 74.5 (C-25), 71.3 (C-K14), 71.0 (C-K2), 70.1 (C-21), 51.1 (chemical shift only observed by HMBC, C-20), 45.0 (C-K7), 42.0 (C-26), 41.7 (C-K4), 38.9 (C-24), 35.1 (C-22), 33.4 (C-K10), 26.8 (C-K8), 25.4 (C-K5), 22.0 (C-13), 20.9 (C-36), 20.4 (C-30), 18.9 (C-K15), 18.7 (C-K1), 13.0 (C-32), 10.0 (C-33), 9.9 (C-34), 7.4 (C-14); HRMS (*m*/*z*): (M + H^+^) calcd. for C_51_H_66_NO_19_, 996.4229, found, 996.4197.

### Antibiotic assays against *Sau*

Minimum inhibitory concentration (MIC) assays were performed by incubating cells against a serial 1:3 dilution of compounds starting at 50 μg mL^−1^. Briefly, a single colony of wild-type *Sau* ATCC 12600 or *Sau* ATCC 12600 carrying either a D471Y, H481Y, or S486L mutation^30^ was used to inoculate 7 mL of Luria-Bertani (LB) broth and the culture was grown overnight to saturation. The following day, 10 μL of overnight culture were diluted into 50 mL of LB broth and 80 μL aliquots were distributed to each well of a 96-well plate. 250 μg of dried test compound was resuspended in 50 μL of methanol and diluted to 250 μg mL^−1^ with LB. Starting with 250 μg mL^−1^ of antibiotic in the first well, a 1:3 serial dilution of the compounds was performed in LB across a separate plate. No compound was added to the final well in each row. 20 μL of diluted test compound were transferred, in triplicate, to the wells of the plate containing the assay strain. This yielded the final volume of 100 μL in assay wells, with the initial concentration of compound being 50 μg mL^−1^. Plates were sealed with air permeable membrane (Breathe-Easy) and incubated at 30 °C with shaking at 200 rpm for 24 h. The OD_600_ of each plate was read at 24 h using an Epoch Microplate Spectrophotometer (BioTek Instruments) and MIC values were reported as the lowest concentration of the compound that inhibited the growth of the test strain.

### Antibiotic assays against *Mtb*

*Mtb* H37Rv was passaged in Middlebrook 7H9 media (BD Biosciences) supplemented with oleic acid-albumin-dextrose-catalase (BD Biosciences) and 0.02% tyloxapol (hereafter called 7H9 complete). All compounds were reconstituted in dimethyl sulfoxide (DMSO) and serial dilutions were created in 96-well microplates. *Mtb* was grown to an OD_580_ of 0.4–0.7 in 7H9 complete at 37 °C in cell culture flasks (Corning). Mid-log phase *Mtb* was diluted to an OD_580_ of 0.01 with 7H9 complete and 198 μL were distributed in 96-well microplates. 2 μL of the compound dilutions were added to the culture wells in triplicate rows, keeping the DMSO concentration at 1%. DMSO and Rif  controls were included in every experiment. Plates were incubated at 37 °C with room air oxygen and 5% CO_2_. IC_90_ values were determined using an M5 SpectraMax Microplate reader (Molecular Devices) at OD_580_ between day 10 and 14 after thorough mixing of the wells.

### *Msm rpoB(S447L)* mutant construction

For construction of the *rpoB(S447L)* allele strain for RNAP purification, MGM6029 (which encodes a chromosomal C terminal fusion to the native *rpoC* gene to ppx-10his)^31^ was transformed with pAJF519, encoding acetamide inducible gp61^67^ and *sacB*. The resulting strain (MGM6205) was then recombineered using oligonucleotides:

oAF1308 (gggtctgacccacaagcgtcgtcttCTggcgctgggccccggcggtctgtcccgtgagcg) and oAF1309 (cgctcacgggacagaccgccggggcccagcgccAGaagacgacgcttgtgggtcagaccc), which encode the *rpoB* S447L mutation^67^. Briefly after induction of the recombinase with 0.2% acetamide for 3 h, cells were washed three times with 10% glycerol and transformed with a mix of oAF1308 and oAF1309. After 4 h of recovery, cells were cultured on 7H10 agar plates containing rifampicin (100 μg mL^−1^). Rif^R^ colonies were screened for the presence of the *rpoB*(S447L) mutation. Once the *rpoB(S447L)* allele was confirmed, pAJF519 was cured by selecting on 10% sucrose. Resulting colonies were then verified as kanamycin-sensitive and rifampicin-resistant to create strain MGM6291 (genotype: mc^2^155 *rpoB(S447L) rpoC:rpoC-ppx-10his hyg)*.

### Transcription assay

Recombinantly produced wild-type and S447L mutant DNA-dependent RNAP were purified from *M. smegmatis* MGM6029 strain expressing a chromosomal copy of *rpoC* with a C-terminal ppx-His_10_-tag, and either wild-type *rpoB* gene or *rpoB* mutant allele (S447L). *M*. *smegmatis* cells were grown to late exponential phase and collected at the Bioexpression and Fermentation Facility at the University of Georgia. Cells were lysed in a French press (Avestin) in 50 mM Tris-HCl, pH 8, 1 mM EDTA, 5% (v/v) glycerol, 5 mM DTT, 1 mM protease inhibitor cocktail, and 1 mM phenylmethylsulfonyl fluoride, and RNAP was precipitated from the cleared lysate by polyethyleneimine (PEI) precipitation (0.35%). The PEI pellet was washed three times with 10 mM Tris-HCl, pH 8, 0.5 M NaCl, 0.1 mM EDTA, 5 mM DTT, and 5% (v/v) glycerol, then eluted three times with the same buffer but with 1 M NaCl. Protein was precipitated overnight with 35% (w/v) ammonium sulfate and resuspended in 20 mM Tris-HCl, pH 8, 5% (v/v) glycerol, 1 M NaCl, and 1 mM β-mercaptoethanol. Protein was loaded on a Ni^2+^-affinity column (HiTrap IMAC HP, GE Healthcare Life Sciences) and eluted in 20 mM Tris-HCl, pH 8, 5% (v/v) glycerol, 0.5 M NaCl, and 0.25 M imidazole. Protein was diluted in 10 mM Tris-HCl, pH 8, 5% (v/v) glycerol, 0.1 mM EDTA, and 5 mM DTT to a final salt concentration of 0.1 M NaCl, loaded on a Biorex (BioRad, Hercules, CA) ion exchange column, and eluted with a salt gradient (0.1–0.8 M). To generate the holoenzyme, the RNAP core was incubated with 5.0 M excess of σ^A^/RbpA^31^ for 15 min at 4 °C and the resulting complex was purified by size exclusion chromatography (Superdex-200, GE Healthcare Life Sciences) in 20 mM Tris-HCl, pH 8, 5% (v/v) glycerol, and 0.5 M NaCl. The purified complex was dialyzed into 20 mM Tris-HCl, pH 8, 100 mM K-glutamate, 10 mM MgCl_2_, and 1 mM DTT and stored at −80 °C.

The transcription assay was performed in 20 μL volumes. 50 nM of the wild-type or mutant RNAP holoenzyme in transcription buffer [10 mM Tris HCl, pH 7.9, 50 mM KCl, 10 mM, MgCl_2_, 1 mM DTT, 5 μg mL^−1^ bovine serum albumin (BSA) and 0.1 mM EDTA] was mixed with Kang A, V1, or V2, or with Rif, at different concentrations of antibiotic. To allow binding of the antibiotics to the RNAP, the mixtures were incubated at 37 °C for 5 min. Following incubation, 10 nM of AP3 promoter^68^ was added to each tube and the samples were incubated for an additional 15 min at 37 °C to allow formation of the RNAP open complex. Transcription was initiated by the addition of a nucleotide mixture consisting of 200 μM ATP, 200 μM CTP, 200 μM GTP, 50 μM unlabeled UTP and 1.25 µCi (0.3 μM) γ-P^32^-UTP. Each reaction was allowed to proceed for 15 min at 37 °C before the addition of 20 μL of stop buffer (0.5× TBE, pH 8.3, 8 M urea, 30 mM EDTA, 0.05% bromophenol blue, and 0.05% xylene cyanol). Reactions were then heated to 95 °C for 10 min and loaded onto a polyacrylamide gel [23% Acrylamide/Bis acrylamide (19:1), 6 M urea, and 1X TBE, pH 8.3]. Gels were run for 3 h at 500 V, then exposed on a phosphoroimaging plate (GE Healthcare) for 12 hrs before being imaged using a Typhoon 9400 Variable Imager (Amersham Biosciences). Uncropped gel images are shown in Supplementary Figure 29.

### Crystallization of *Msm* RbpA/TIC

Crystals of the *Msm* RbpA/TIC were prepared with purified *Msm* RbpA/σ^A^-holo and the upstream fork DNA. The DNA was assembled from synthetic oligos (5′-GCTTGACAAAAGTGTTAAATTGTGCTATACT-3′ and 5′-AGCACAATTTAACACTTTTGTCAAGC-3′; Integrated DNA Technologies, Coralville, IA) by annealing in 10 mM Tris-HCl, pH 8, 1 mM EDTA, and 0.2 M NaCl. Protein was mixed in a 1:1 molar ratio with annealed upstream fork DNA for 15 min at room temperature to generate the *Msm* RbpA/TIC. Crystals were grown by hanging drop vapor diffusion by mixing 1 µL of *Msm* RbpA/TIC solution (11 mg ml^−1^ protein) with 1 µL of crystallization solution [0.1 M Bis–Tris, pH 6.0, 0.2 M LiSO_4_, 16% (w/v) polyethylene glycol 3350, 2.5% (v/v) ethylene glycol] and incubating over a well containing crystallization solution at 22 °C. Stabilization solutions [0.1 M Bis–Tris, pH 6.0, 0.2 M LiSO_4_, 20% (w/v) polyethylene glycol 3350, 2.5% (v/v) ethylene glycol, 1% (v/v) DMSO] with either 0.1 mM Rif or 0.1 mM Kng A were prepared by adding Rif or Kang A from 10 mM stock solutions in 100% DMSO. Fully grown crystals were transferred into stabilization solution and incubated for 4 h to allow antibiotic binding. The crystals were then cryo-protected by step-wise transfer (three steps) into 0.1 M Bis–Tris, pH 6.0, 0.2 M LiSO_4_, 22% (w/v) polyethylene glycol 3350, 20% (v/v) ethylene glycol, 1% (v/v) DMSO and either 0.1 mM Rif or Kang A, and flash frozen by plunging into liquid nitrogen. Crystals of the S447L β RNAP variant of the *Msm* RbpA/TIC were grown and frozen as described for the wild-type RNAP.

### Data collection, structure determination, and refinement

X-ray diffraction data were collected at the Argonne National Laboratory Advanced Photon Source (APS) NE-CAT beamline 24-ID-C (Rif/TIC) or the National Synchrotron Light Source II (NSLSII) AMX beamline 17-ID-1 (Kang A/TIC). Structural biology software was accessed through the SBGrid consortium^69^. Data were integrated and scaled using HKL2000^70^.

Starting with 5TW1^31^, the models were first improved by rigid body refinement of 20 individual mobile domains using PHENIX^71^. The resulting models were improved by iterative cycles of manual building with COOT^72^ and refinement with PHENIX. Difference Fourier maps revealed excellent electron density for Rif or Kang A. Rif^73^ or Kang A^23^ crystal structures were easily modeled into the respective difference densities, but the Kang A structure required inversion of the coordinates through the origin (i.e., the deposited coordinates are the mirror image of the molecule). Further iterative cycles of building and refinement yielded the final models (Supplementary Table 5).

### DNase I footprinting

AC50 promoter DNA with a 5′ ^32^P-labeled template strand was prepared by PCR amplification using a 5′-end-labeled PCR primer (5′-GGCGCTACGGCGTTTCACTTCTGAGTTCGGCATG-3′). The primer was initially labeled with ^32^P using substrate [γ-32P] ATP and T4 polynucleotide kinase, followed by purification using a NucAway nucleotide removal kit. The resulting PCR product was loaded on a nondenaturing 5% acrylamide gel and the DNA was eluted from the gel by the crush-soak method. DNaseI (New England Biolabs) was diluted to 200 U μl^−1^ and kept on ice. Reactions (20 μl) were carried out in a 37 °C water bath and in 1X footprinting buffer (10 mM Tris-HCL, pH 8, 10 mM K-glutamate, 5 mM MgCl_2_, 0.1 mM DTT, 15 μg mL^−1^ BSA). *Eco* core RNAP (400 nM) and *σ*^70^ (2 μM) were incubated for 5 min to form holoenzyme followed by the addition of the ^32^P-labeled promoter DNA (200 fmol). *Msm* RbpA/σ^A^-holoenzyme (400 nM) was mixed with either 100 µM Kang A or 100 µM Rif for 5 min at 37 °C followed by the addition of the ^32^P-labeled promoter DNA (200 fmol). The control reaction without antibiotics was also done. Formation of RPo was allowed to proceed for 15 min. DNase I (200 U) was then added to the mixture and the reactions were incubated for an additional 2 min. The reactions were quenched by the addition of 100 μl of 0.5 M phenol, and 80 μl of a mixture of sodium acetate (375 mM) and EDTA (12.5 mM final), and 2 μl glycoblue. The DNA was recovered in the aqueous layer, ethanol precipitated and washed. The air-dried pellet was resuspended in 2X loading buffer, heated at 95 °C for 1 min before being immediately loaded on an 8% polyacrylamide (19:1 acrylamide:bis-acrylamide) 8 M urea gel. The gels were visualized by phosphorimagery and digitized with a Typhoon phosphorimager.

### Fe^2+^-mediated hydroxyl-radical cleavage

Fe^2+^-mediated hydroxyl-radical cleavage of the template strand DNA was performed with 5′-end-labeled template strand AP3 promoter^74^ prepared in the same way as described for the DNase I footprinting. Promoter DNAs with 5′-end-labeled t-strand were amplified and purified as described above. *Msm* RbpA/σ^A^-holoenzyme (500 nM) was dialyzed against 8 mM Hepes, pH 7.5, 1 mM DTT for 2 h at room temperature. Reactions (20 μl) were performed at 37 °C. The samples were mixed with either 10 μM Kang A or 10 μM Rif, followed by 5 min incubation at 37 °C. The control reaction without antibiotic was also done. Formation of RPo was started by the addition of the ^32^P-labeled promoter DNA (200 fmol) followed by 15 min incubation at 37 °C. The complex was then treated for 5 min at 37 °C with 20 μM Fe(NH_4_)_2_(SO_4_)_2_. The reactions were stopped by the addition of 80 μl of 20 mM thiourea and 20 μl of 0.3 M NaCl/1 mM EDTA. The DNA was then precipitated with ethanol, dissolved in 2× loading buffer, and analyzed on an 8% sequencing gel.

### De novo transcription initiation assay

Promoter DNA (−87 to +71 of *Mtb rrn*AP3)^68^ with an engineered mutation, +3 T > A, was synthesized (GenScript) and placed into the pUC57 plasmid to generate pUC57-AP3 GU. Fragment −87 to +71 of pUC57-AP3 GU was PCR amplified. The promoter DNA fragment was subsequently separated on an agarose gel and gel purified (Qiagen). This promoter fragment served as the template for de novo initiation with GTP, GDP, or GMP as the 5′-initiating (*i* site) nucleotides. *Msm* RbpA/σ^A^-holoenzyme was diluted into 1× transcription buffer (10 mM Tris-HCl, pH 8.0, 100 mM K-glutamate, 10 mM MgCl_2_, 1 mM DTT, 0.1 mM EDTA, 5 μg mL^−1^ BSA). Reactions (20 µl) were carried out in a 37 °C water bath with proteins using the following protocol: *Msm* RbpA/σ^A^-holoenzyme (50 nM) and increasing concentrations of Kang A or Rif were combined and incubated at 37 °C for 5 min. Next, promoter DNA (10 nM) was added and RPo was allowed to form for 15 min at 37 °C. Abortive transcription was initiated by the addition of a mixture containing one of the 5′-initiating substrates (1 mM GTP, 2 mM GDP, or 4 mM GMP), plus 50 µM of unlabeled UTP and 1.25 μCi αP^32^-UTP. After 10 min, transcription was quenched by the addition of 2× stop buffer (8 M Urea, 0.5X TBE, 0.05% bromophenol blue, 0.05% xylene cyanol). Reactions were heated at 95 °C for 2 min and loaded on a 25% polyacrylamide gel (19:1 acrylamide:bis-acrylamide). Abortive products were visualized by phosphorimagery and digitized using a Typhoon phosphorimager. Data were quantified using ImageJ^75^. Uncropped gel images are shown in Supplementary Figure 30.

## Electronic supplementary material

Supplementary Information

## Data Availability

The X-ray crystallographic coordinates and structure factor files have been deposited in the Protein Data Bank with accession codes 6CCV (Rif/TIC), 6CCE (Kang A/TIC), 6DCF (Kang A/S447L mutant). Metagenomic DNA sequences described in this manuscript have been deposited in GenBank with the accession numbers MH480516 to MH480581.
