# Supplementary material for: Rifamycin congeners kanglemycins are active against rifampicin-resistant bacteria via a distinct mechanism
Source: Nat Commun. 2018 Oct 8;9:4147. doi: 10.1038/s41467-018-06587-2 (PMC6175910; doi:10.1038/s41467-018-06587-2)
Supplement: Supplementary file 1 — Supplementary Information [file 41467_2018_6587_MOESM1_ESM.pdf]

Supplementary Information for:

Rifamycin congeners kanglemycins are active against rifampicin-resistant bacteria via a distinct mechanism

Peek, Lilic *et al*.

Table of Contents:

1. Supplementary Table 1. Oligonucleotides used in this study
2. Supplementary Figure 1. Summary of tailoring gene content of rifamycin family gene clusters recovered from soil metagenomes
3. Supplementary Figure 2. Phylogenetic analysis of eDNA-derived cytochrome P450 and oxidoreductase genes
4. Supplementary Figure 3. Phylogenetic analysis of eDNA-derived methyltransferase, glycosyltransferase, and sugar biosynthesis genes
5. Supplementary Figure 4. HPLC analysis of the C-18 flash column fraction containing Kangs A, V1, and V2
6. Supplementary Table 2. ^{13}C and ^{1}H and chemical shifts of Kangs A, V1, and V2
7. Supplementary Figure 5. NMR and HRMS data used to establish the structures of Kang A
8. Supplementary Figure 6. ^{1}H NMR spectrum of Kang A in CD_2Cl_2, collected at 25 °C
9. Supplementary Figure 7. ^{13}C NMR spectrum of Kang A in CD_2Cl_2, collected at 25 °C
10. Supplementary Figure 8. HMQC NMR spectrum of Kang A in CD_2Cl_2, collected at 25 °C
11. Supplementary Figure 9. COSY NMR spectrum of Kang A in CD_2Cl_2, collected at 25 °C
12. Supplementary Figure 10. HMBC spectrum of Kang A in CD_2Cl_2, collected at 25 °C
13. Supplementary Figure 11. NMR and HRMS data used to establish the structures of Kang V1
14. Supplementary Figure 12. ^{1}H NMR spectrum of Kang V1 in CD_3OD, collected at 25 °C
15. Supplementary Figure 13. ^{13}C NMR spectrum of Kang V1 in CD_3OD, collected at 25 °C
16. Supplementary Figure 14. HSQC NMR spectrum of Kang V1 in CD_3OD, collected at 25 °C
17. Supplementary Figure 15. COSY NMR spectrum of Kang V1 in CD_3OD, collected at 25 °C
18. Supplementary Figure 16. HMBC spectrum of Kang V1 in CD_3OD, collected at 25 °C
19. Supplementary Figure 17. NMR and HRMS data used to establish the structures of Kang V2
20. Supplementary Figure 18. ^{1}H NMR spectrum of Kang V2 in CD_3OD, collected at -20 °C
21. Supplementary Figure 19. ^{13}C NMR spectrum of Kang V2 in CD_3OD, collected at -20 °C
22. Supplementary Figure 20. HSQC NMR spectrum of Kang V2 in CD_3OD, collected at -20 °C
23. Supplementary Figure 21. COSY NMR spectrum of Kang V2 in CD_3OD, collected at -20 °C
24. Supplementary Figure 22. HMBC spectrum of Kang V2 in CD_3OD, collected at -20 °C
25. Supplementary Figure 23. Proposed biosynthesis of 3-amino-5-hydroxybenzoic acid (AHBA) and the K-sugar and K-acid moieties in *Amycolatopsis vancoresmycina*
26. Supplementary Figure 24. Proposed biosynthesis and tailoring of the Kang polyketide core in *A. vancoresmycina*
27. Supplementary Table 3. Gene annotations for the *kng* biosynthetic gene cluster from *A. vancoresmycina* and corresponding genes from the *A. mediterranei* rifamycin cluster
28. Supplementary Table 4. Antibacterial activity of Kangs A, V1, and V2
29. Supplementary Figure 25. Mutations found in RNAP (*rpoB*) genes sequenced from Rif^R or $Kang^R$ *S. aureus*
30. Supplementary Table 5. Table of crystallographic statistics
31. Supplementary Figure 26. Electron density and cation-π bridges
32. Supplementary Figure 27. Electron density of S447L RNAP with Kang A
33. Supplementary Figure 28. RNAP footprint of promoter DNA in the presence of antibiotics
34. Supplementary Table 6. Gene annotations for the metagenomic gene cluster RifCon12 and corresponding genes from the *A. vancoresmycina kng* cluster
35. Supplementary Table 7. Gene annotations for the metagenomic gene cluster RifCon6 and corresponding genes from the *A. vancoresmycina kng* cluster
36. Supplementary Table 8. Gene annotations for the metagenomic gene cluster RifCon10 and corresponding genes from the *A. vancoresmycina kng* cluster
37. Supplementary Figure 29. Uncropped scans of transcription assay gels shown in manuscript Figure 3E.
38. Supplementary Figure 30. Uncropped scans of transcription assay gels shown in manuscript Figure 6D.
39. References

Supplementary Table 1. Oligonucleotides used in this study.

rifK (AHBA synthase) forward<br>**CTACACGACGCTCTTCCGATCT***TCCGTCTA*ACCSGCCTTCACCTTCATCTCCTC<br>(bold, MiSeq adaptor; italic, variable barcode; underlined, variable spacer)
rifK (AHBA synthase) reverse<br>**CAGACGTGTGCTCTTCCGATCT***AACGTGAT*AAYCCGGAACATSGCCATGTAGTG<br>(bold, MiSeq adaptor; italic, variable barcode; underlined, variable spacer)
KS domain forward<br>**CCATCTCATCCCTGCGTGTCTCCGACTCAG***GTCACACA*GATATCGAGGCSCAGGCSYTG<br>(bold, PGM adaptor; italic, variable barcode; underlined, variable spacer)
KS domain reverse<br>**CCTCTCTATGGGCAGTCGGTGAT**GAYSASGTGSGCGTTSGT<br>(bold, PGM adaptor)
rif15A/B forward<br>**CTACACGACGCTCTTCCGATCT***AAGACGGA*TCCCGGTTCTAYCTSTCCAAG<br>(bold, MiSeq adaptor; italic, variable barcode; underlined, variable spacer)
rif15A/B reverse<br>**CAGACGTGTGCTCTTCCGATCT***AAACATCG*TCAASRACCACGASGAGATGT<br>(bold, MiSeq adaptor; italic, variable barcode; underlined, variable spacer)
oAF1308 recombineering oligonucleotide<br>GGGTCTGACCCACAAGCGTCGTCTTCTGGCGCTGGGCCCCGGCGGTCTGTCCCGTGAGCG
oAF1309 recombineering oligonucleotide<br>CGCTCACGGGACAGACCGCCGGGGCCCAGCGCCAGAAGACGACGCTTGTGGGTCAGACCC
Usfork_T10-top<br>GCTTGACAAAAGTGTTAAATTGTGCTATACT
Usfork_T10-bot<br>AGCACAATTTAACACTTTTGTCAAGC

A

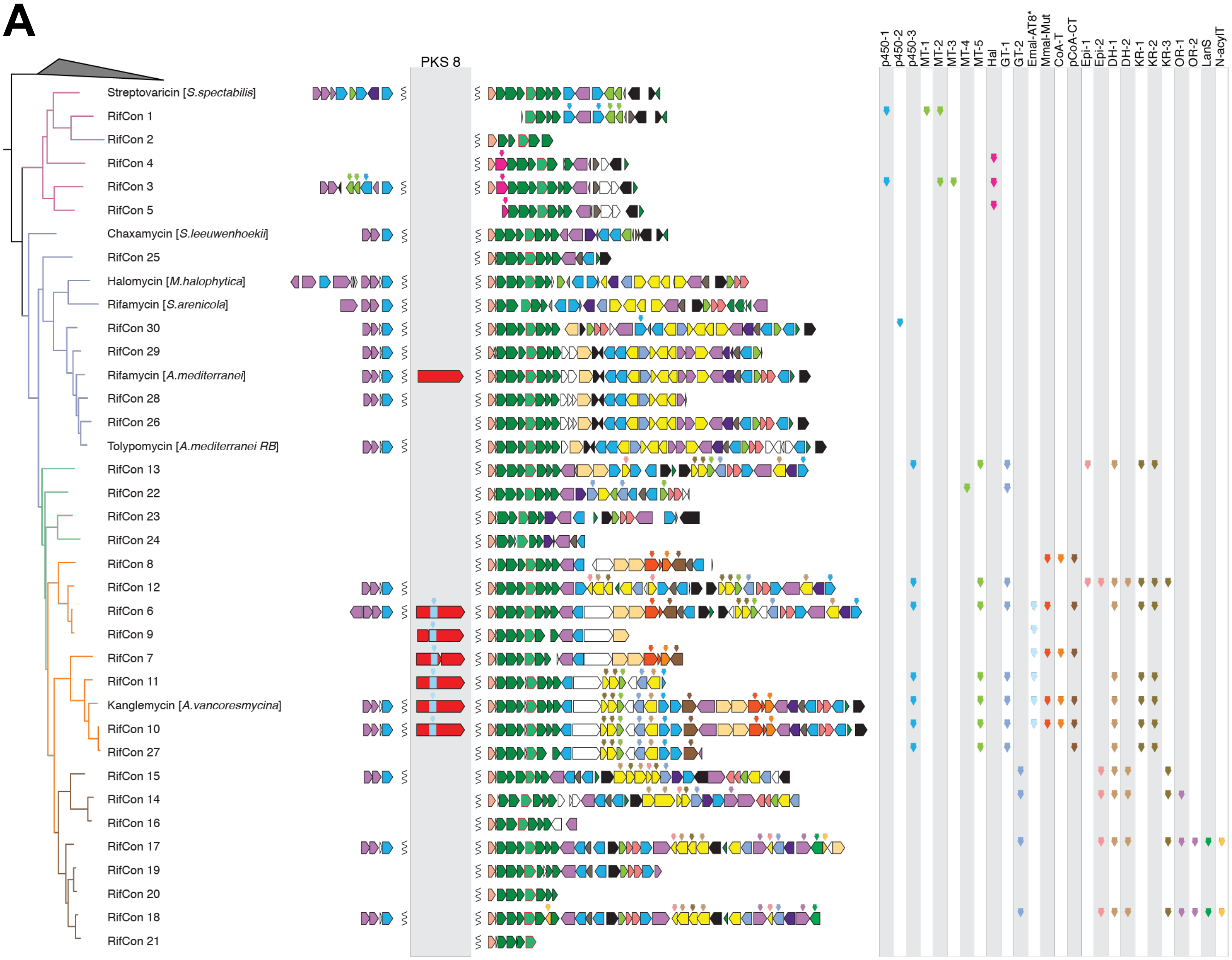

B

A. mediterranei Rif	AEALDQALEALTGQDIRVRRVAVD**YASH**TRHVEDIQEPLAEALAGIEAHAPTLPFFS
RifCon_6	TSALQRFLDECVAAGVRASVVAST**VASH**GVQVEPLRDELLGLLKDVRPGPGTVPFYS
RifCon_10	DESLRRFLAECEAADVRARIVPST**VGSH**TAQVEPLRDELLDLLKDVRPEAGTVPFYS
RifCon_12	AEALDEALELLSAEGVRVRRVAVD**YASH**TRHVEDIRDTLAEALAGITAQEPVIPFYS
A. vancoresmycina Kng	DESLQRFLAECAAADVRARIVPST**VGSH**TAQVEPLRDELLDLLKDVRPEPGTVPFYS

Supplementary Figure 1. Summary of tailoring gene content of rifamycin family gene clusters recovered from soil metagenomes. A. Tailoring genes that are absent or phylogenetically distinct (based on the phylogenies shown in Supplementary Figures 2-9) from the prototypical rifamycin gene cluster from *Amycolatopsis mediterranei* are indicated in the table on the right. Previously characterized rifamycin family gene clusters are shown for reference. PKS modules, with the exception of the eighth module, which shows significant functional differences, are omitted for simplicity. Abbreviations: P450, cytochrome P450; MT, methyltransferase; Hal, halogenase; GT, glycosyltransferase; Emal-AT8*, ethylmalonyl-CoA specific acyltransferase domain; Mmal-Mut, methylmalonyl-CoA mutase; CoA-T, CoA transferase; pCoA-CT, priopionyl-CoA carboxyltransferase; Epi, sugar epimerase; DH, sugar dehydratase; KR, sugar ketoreductase; OR, oxidoreductase; LanS, lanthionine synthetase; N-acylT, *N*-acyltransferase. **B.** Partial amino acid sequence alignment of the AT8 domains encoded by the *A. mediterranei* rifamycin gene cluster (Rif), the metagenomic RifCon 6, RifCon 10, and RifCon 12 gene clusters, and the *A. vancoresmycina* kanglemycin (Kng) gene cluster. Substrate specifying motifs for methylmalonyl-CoA and ethylmalonyl-CoA are shown in red and blue, respectively.[1, 2]

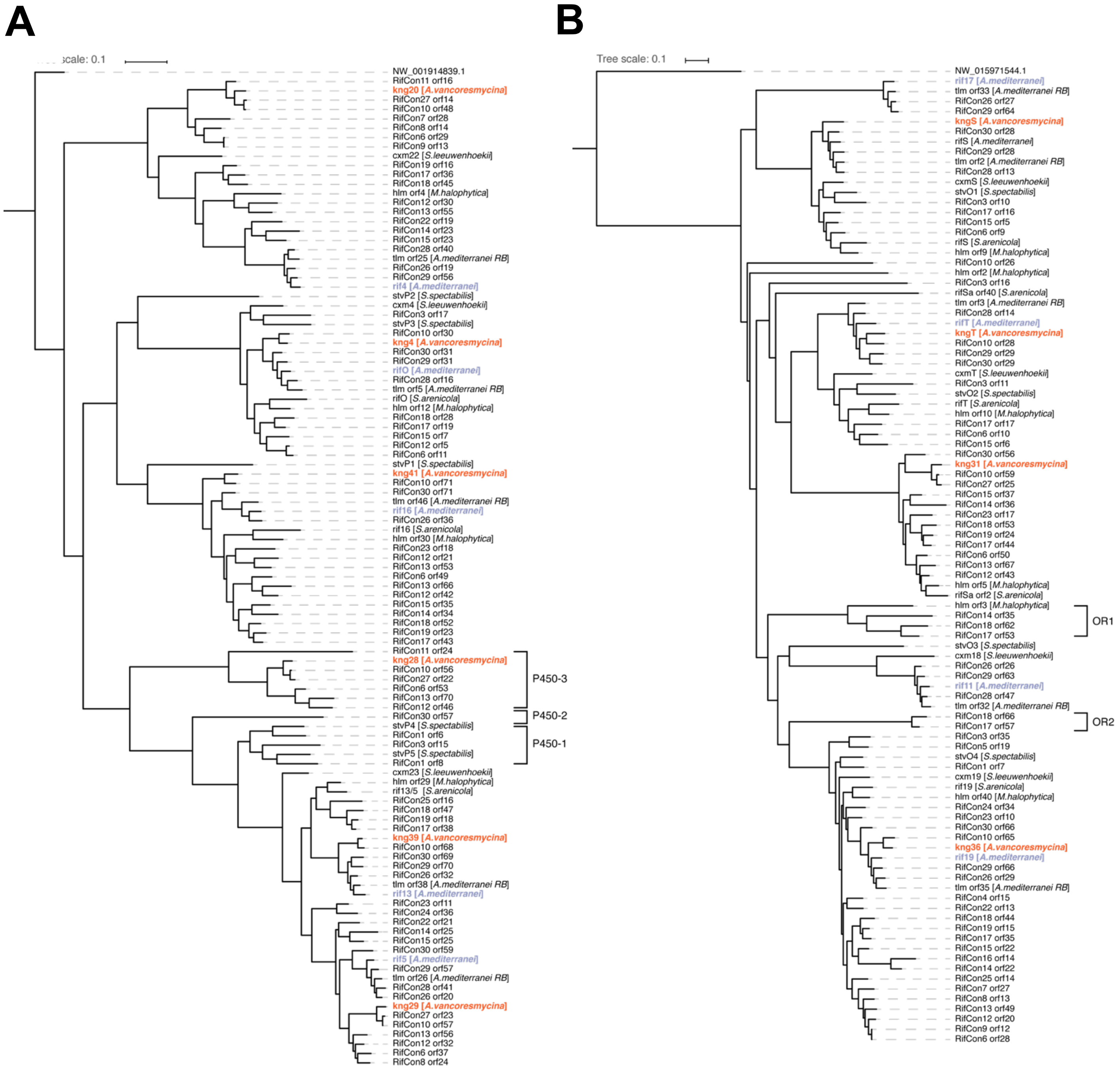

Supplementary Figure 2. Phylogenetic analysis of eDNA-derived cytochrome P450 and oxidoreductase genes. A. Cytochrome P450 genes. **B.** Oxidoreductase genes. Genes from previously characterized rifamycin family gene clusters are shown for reference. Clades that lack an obvious homolog from the *A. mediterranei* rifamycin gene cluster are labeled. These labels correspond with the labels in the table shown in Supplementary Figure 1.

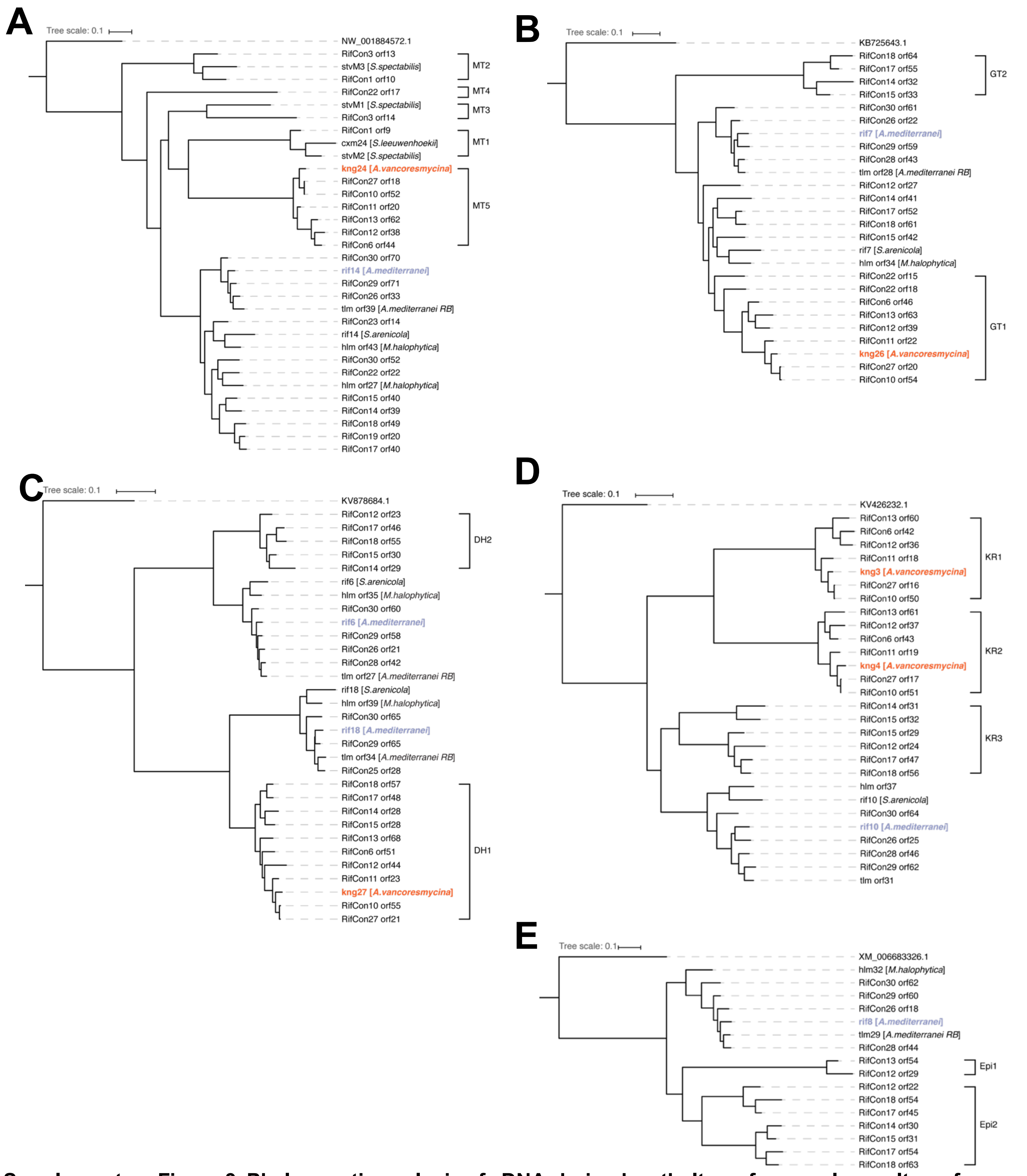

Supplementary Figure 3. Phylogenetic analysis of eDNA-derived methyltransferase, glycosyltransferase, and sugar biosynthesis genes. A. Methyltransferase genes. **B.** Glycosyltransferase genes. **C.** Sugar dehydratase genes. **D.** Sugar ketoreductase genes. **E.** Sugar epimerase genes. Genes from previously characterized rifamycin family gene clusters are shown for reference. Clades that lack an obvious homolog from the *A. mediterranei* rifamycin gene cluster are labeled. These labels correspond with the labels in the table in Supplementary Figure 1.

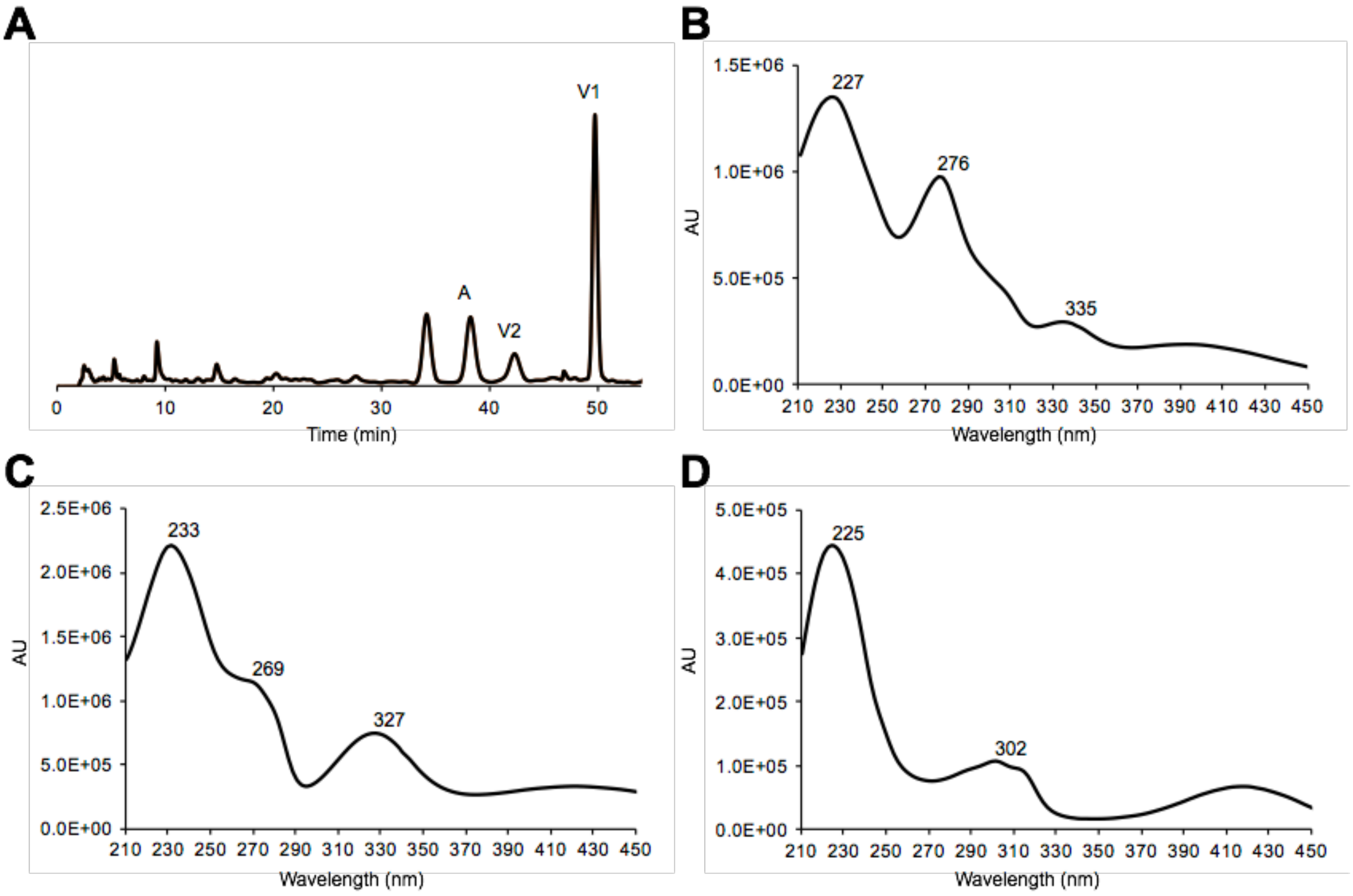

Supplementary Figure 4. HPLC analysis of the C-18 flash column fraction containing Kangs A, V1, and V2. A. HPLC chromatogram of partially purified extracts from *A. vancoresmycina* cultures monitored at 420 nm. Crude ethyl acetate extracts from *A. vancoresmycina* cultures were first fractionated by flash chromatography. Fractions with strong UV absorbance at 254 nm and 420 nm were then pooled and subjected to HPLC. Peaks corresponding to Kangs A, V1, and V2 are labeled. **B.** Kang A UV spectrum. **C.** Kang V1 UV spectrum. **D.** Kang V2 UV spectrum.

Supplementary Table 2. ^{13}C and ^{1}H and chemical shifts of Kangs A, V1, and V2.

	Kang A[a]			Kang V1[b]			Kang V2[c]		
Position	Atom type	δ_C	δ_H (mult., *J* in Hz)	Atom type	δ_C	δ_H (mult., *J* in Hz)	Atom type	δ_C	δ_H (mult., *J* in Hz)
1	C	185.8		C	183.8		C	150.4	
2	C	140.9		C	142.9		C	126.4	
3	CH	117.0	7.80 (s)	CH	118.2	7.69 (s)	CH	118.2	7.99 (s)
4	C	184.9		C	188.8		C	150.5	
5	C	111.7		C	109.4		C	106.4	
6	C	171.8		C	164.9		C	167.9	
7	C	116.9		C	114.3		C	108.3	
8	C	167.4		C	165.8		C	191.7	
9	C	111.2		C	126.3		C	114.1	
10	C	132.0		C	127.1		C	115.4	
11	C	194.1		CH	77.1	5.49 (s)	C	165.7	
12	C	109.9		C	113.9		C	112.9	
13	CH_3	23.7	1.67 (s)	CH_3	25.0	1.86 (s)[e]	CH_3	22.0	1.86 (s)
14	CH_3	7.8	2.34 (s)	CH_3	8.4	2.20 (s)	CH_3	7.4	2.01 (s)
15	C	171.6		C	172.0		C	171.7	
16	C	137.0		C	133.6		C	134.7	
17	CH	129.8	6.16 (d, 5.8)	CH	133.0	6.33 (d, 11.3)	CH	132.0	6.30 (d, 11.2)
18	CH	128.2	5.93 (dd, 15.9, 5.8)	CH	130.8	6.41 (dd, 15.4, 11.3)	CH	131.3	6.52 (dd, 15.6, 11.2)
19	CH	134.1	5.81 (dd, 15.9, 9.3)	CH	134.4	5.98 (dd, 15.4, 8.4)	CH	132.5	5.94 (dd, 15.6, 8.1)
20	CH	53.0	2.15 (m)	CH	51.6	2.33 (m)	CH	51.1[j]	2.33 (m)
21	CH	68.9	3.68 (m)	CH	70.2	4.06 (dd, 7.6, 3.9)	CH	70.1	4.06 (m)
22	CH	33.8	1.84 (m)	CH	35.3	1.86 (m)[e]	CH	35.1	1.81 (m)
23	CH	79.0	2.85 (dd, 10.0, 1.8)	CH	78.3	3.07 (dd, 11.1, 1.8)	CH	77.9	3.06 (dd, 10.2, 1.8)
24	CH	36.9	1.60 (m)	CH	38.4	1.64 (m)	CH	38.9	1.43 (m)
25	CH	74.1	4.36 (dd, 9.5, 1.0)	CH	74.9	5.11 (dd, 10.5, 1.6)	CH	74.5	5.12 (dd, 10.3, 1.7)
26	CH	37.0	2.13 (m)	CH	41.2	1.40 (m)	CH	42.0	1.20 (m)
27	CH	81.5	3.85 (dd, 9.3, 2.7)	CH	78.0	3.90 (m)	CH	78.2	3.92 (dd, 5.5, 2.0)
28	CH	112.9	5.13 (dd, 12.8, 9.3)[d]	CH	118.4	5.16 (dd, 12.3, 4.0)	CH	123.3	5.36 (dd, 12.5, 5.5)
29	CH	146.4	6.37 (d, 12.8)	CH	143.3	6.15 (d, 12.3)	CH	141.8	6.22 (d, 12.5)
30	CH_3	21.1	2.06 (s)	CH_3	20.2	2.07 (s)	CH_3	20.4	2.08 (s)
32	CH_3	12.9	0.94 (d, 7.0)	CH_3	13.5	1.01 (d, 7.1)	CH_3	13.0	0.99 (d, 6.7)
33	CH_3	9.4	0.69 (d, 6.7)	CH_3	10.1	0.60 (d, 6.8)	CH_3	10.0	0.51 (br s)
34	CH_3	13.4	0.38 (d, 7.2)	CH_3	10.3	0.22 (d, 7.0)	CH_3	9.9	-0.06 (d, 5.6)
35	C	174.1		C	173.5		C	172.7	
36	CH_3	21.8	2.02 (s)	CH_3	20.9	2.03 (s)	CH_3	20.9	2.06 (s)
K1	CH_3	19.8	1.07 (d, 6.3)	CH_3	18.8	1.19 (d, 6.9)	CH_3	18.7	1.18 (d, 7.1)[h]
K2	CH	68.0	5.06 (dd,12.7, 6.3)	CH	71.1	5.06 (m)[f]	CH	71.0	5.02 (dd, 5.9, 4.1)
K3	C	176.4		C	177.9		C	177.9	
K4	C	40.8		C	41.8		C	41.7	
K5	CH_3	26.2	1.17 (s)	CH_3	26.6	1.27 (s)[g]	CH_3	25.4	1.29 (s)
K6	C	172.3		C	174.6		C	174.8	
K7	CH_2	43.5	2.66 (d, 16.9)	CH_2	44.9	2.75 (d, 16.5)	CH_2	45.0	2.69 (d, 16.9)
			2.53 (d, 16.9)			2.57 (d, 16.5)			2.56 (d, 16.9)
K8	CH_3	24.9	1.23 (s)	CH_3	25.3	1.27 (s)[g]	CH_3	26.8	1.31 (s)
K9	CH	97.2	4.65 (dd, 9.0, 1.1)	CH	101.0	4.52 (dd, 8.0, 2.3)	CH	101.0	4.48 (dd, 7.8, 1.4)
K10	CH_2	33.5	2.23 (ddd, 15.5, 2.9, 1.1)	CH_2	33.6	2.10 (ddd, 13.5, 8.0, 4.8)	CH_2	33.4	2.13 (m)
			1.83 (m)			1.74 (m)			1.80 (m)
K11	CH	74.9	4.10 (m)	CH	75.2	4.02 (m)	CH	75.2	3.99 (m)
K12	CH_2	95.4	5.13 (s)[d]	CH_2	95.7	5.06 (s)[f]	CH_2	95.7	5.06 (s)
			4.87 (s)			4.79 (s)			4.76 (s)
K13	CH	75.9	3.64 (m)	CH	77.0	3.55 (dd, 9.0, 5.5)	CH	76.7	3.54 (dd, 9.1, 5.3)
K14	CH	70.5	3.36 (m)	CH	71.2	3.31[i]	CH	71.3	3.30[i]
K15	CH_3	18.8	1.27 (d, 6.2)	CH_3	18.9	1.20 (d, 6.3)	CH_3	18.9	1.18 (d, 6.2)[h]
K16							CH_2	98.4	6.19 (d, 6.6)
									5.48 (d, 6.6)
NH	NH		8.34 (s)						
OH-8	OH		12.60 (s)						

[a] ^{1}H and ^{13}C NMR data were obtained at 600 and 150 MHz, respectively, using CD_2Cl_2 as a solvent and a collection temperature of 25 °C. Reference chemical shifts for CD_2Cl_2 were δ_H 5.32 and δ_C 54.0. The concentration of Kang A was 4.5 mM.

[b] ^{1}H and ^{13}C NMR data were obtained at 600 and 150 MHz, respectively, using CD_3OD as a solvent and a collection temperature of 25 °C. Reference chemical shifts for CD_3OD were δ_H 3.31 and δ_C 49.0. The concentration of Kang V1 was 4.5 mM.

[c] ^{1}H and ^{13}C NMR data were obtained at 500 and 125 MHz, respectively, using CD_3OD as a solvent and a collection temperature of -20 °C. Reference chemical shifts for CD_3OD were δ_H 3.31 and δ_C 49.0. The concentration of Kang V2 was 2.9 mM.

[d,e,f,g,h] Overlapped signals

[i] Signals overlapped with solvent peak.

[j] ^{13}C chemical shift only observed by HMBC

HRMS analysis
Molecular formula
$C_{50}H_{64}NO_{19}$ (M+H$^+$)
Calculated *m/z*
982.4073
Found *m/z*
982.4025

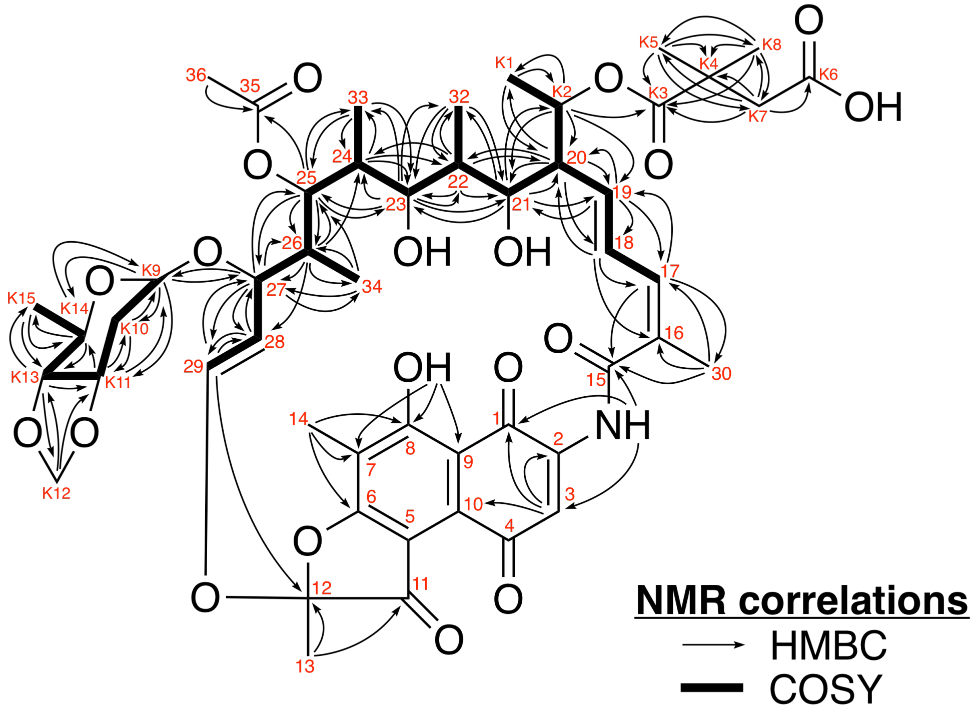

Supplementary Figure 5. NMR and HRMS data used to establish the structure of Kang A. The structure of Kang A was elucidated using a combination of HRESIMS, UV and NMR data. HRESIMS data indicated a chemical formula of $C_{50}H_{63}NO_{19}$. The UV spectrum showed absorption maxima at 227 nm, 276 nm, and 335 nm, typical of a naphthoquinone core seen in other rifamycin family molecules (Supplementary Figure 4).[3] ^{13}C NMR data revealed signals at δ 185.8 and 184.9, representing the quinone carbonyls at C-1 and C-4 (Supplementary Table 2). A quinone substructure was further supported by HMBC correlations from a highly deshielded singlet proton H-3 [δ 7.80 (1H, s)] to C-1, C-2 [δ 140.9], and C-10 [δ 132.0]. The second ring of the chromophore was established based on extensive HMBC correlations from the protons of the C-14 methyl group [δ 2.34 (3H, s)] and the hydroxyl on C-8 [δ 12.60 (1H, s)] to C-6 [δ 171.8], C-7 [δ 116.9] C-8 [δ 167.4] and C-9 [δ 111.2]. The presence of a 5-membered ring attached to the naphthoquinone system was established using HMBC correlations from the H-13 methyl protons [δ 1.67 (3H, s)] to the C-11 ketone [δ 194.1] and to C-12 [δ 109.9]. Comparison of Kang A chemical shift data with NMR data from previously characterized rifamycin congeners[3-5] helped define the final naphthoquinone substructure as shown.

The COSY spectra for Kang A revealed two spin systems. The larger of the two spin systems contained the majority of a rifamycin-like PK backbone. This substructure, including the presence of methyl substituents at C-32, C-33, and C-34 was supported by extensive HMBC correlations. The rare ethyl (C-K1, C-K2) branch in the PK backbone, which was suggested by the predicted substrate specificity of the PKS AT8* domain, was apparent from COSY correlations involving H-K1 [δ 1.07 (3H, d)], H-K2 [δ 5.06 (1H, dd)], and H-20 [δ 2.15 (1H, m)] and was supported by extensive HMBC correlations. Placement of the acetoxy group at C-25 was supported by HMBC correlations from H-36 [δ 2.02 (3H, s)] and H-25 [δ 4.36 (1H, dd)] to the C-35 carbonyl [δ 174.1]. The coupling constant between H-28 and H-29 (12.8 Hz) closely matched that observed in previously characterized rifamycin congeners, suggesting that C28-C29 adopts a *trans* configuration as seen in all known rifamycin family members.[3-8] HMBC correlations from the C-17 end of the COSY spin system to the C-16 [δ 137.0] olefinic carbon, the C-30 methyl carbon [δ 21.1], and the C-15 carbonyl [δ 171.6] allowed us to extend this spin system by 3 carbons. The chemical shift of the C-15 carbonyl is consistent with it being involved in an amide

bond as seen in other rifamycin congeners. HMBC correlations from the amide proton [δ 8.34 (1H, s)] to C-15 and to C-1 and C-3 [δ 117.0] in the naphthoquinone allowed us to connect one end of the PK backbone to the naphthoquinone substructure. An HMBC correlation from H-29 [δ 6.37 (1H, d)] to C-12 allowed us to connect the other end of the PK backbone to the naphthoquinone substructure through an oxygen.

The structure of the K-acid was partially defined by HMBC correlations from the two protons on C-K7 [δ 2.66 (1H, d), δ 2.53 (1H, d)] to the carboxylic acid at C-K6 [δ 172.3]. Additional HMBC correlations from the protons on C-K7, as well as from protons from two methyl singlets [H-K5, δ 1.17 (3H, s), H-K8, δ 1.23 (3H, s)], were used to position the C-K4 quaternary carbon [δ 40.8], and the second carbonyl carbon [C-K3, δ 176.4] in the K-acid. Attachment of the K-acid to the ethyl modification in the PK backbone was established by an HMBC correlation from H-K2 to C-K3.

The K-sugar moiety was established based on a second COSY spin system consisting of C-K9 through C-K15. HMBC correlations between H-K14 [δ 3.36 (1H, m)] and C-K9 [δ 97.2] as well as H-K9 [δ 4.65 (1H, dd)] and C-K14 [δ 70.5] allowed us to connect C-K9 and C-K14 through an oxygen. The methylenedioxy bridge of the K-sugar was established by HMBC correlations from a pair of deshielded protons found on C-K12 [δ 5.13 (1H, s), δ 4.87 (1H, s)] to C-K11 [δ 74.9] and C-K13 [δ 75.9]. Additional HMBC correlations from H-27 [δ 3.85 (1H, dd)] to C-K9 and from H-K9 to C-27 [δ 81.5] were used to define the point of attachment of the K-sugar to the PK backbone.

The HRESIMS and NMR arguments presented here are consistent with the structure of the natural product Kang A, originally isolated from *Amycolatopsis mediterranei* var. *kanglensis*.[9] In our detailed analysis we identified several differences between our ^{13}C assignments and those presented in the original publication, which only reported ^{13}C data along with the X-ray crystal structure of Kang A. We believe the more extensive 2D NMR analysis we have carried out provides a more accurate assignment of the ^{13}C chemical shifts and the first assignment of the ^{1}H chemical shifts. The key difference in our Kang A structure and that reported previously is the configuration of the C-28:C-29 olefin. In the planar structure drawn in the original Kang A paper, the C-28:C-29 olefin appears in the *Z* configuration. Based on other rifamycin congener data sets,[3-6] the 12.8 Hz coupling constant we observed between H-28 and H-29 indicates an *E* configuration. Our analysis of the original crystallographic data deposited for Kang A (Cambridge Crystallographic Data Centre identifier GAGFEZ) indicates that the C28-C29 bond does in fact appear to adopt the *E* configuration rather than the *Z* configuration drawn in the original publication.

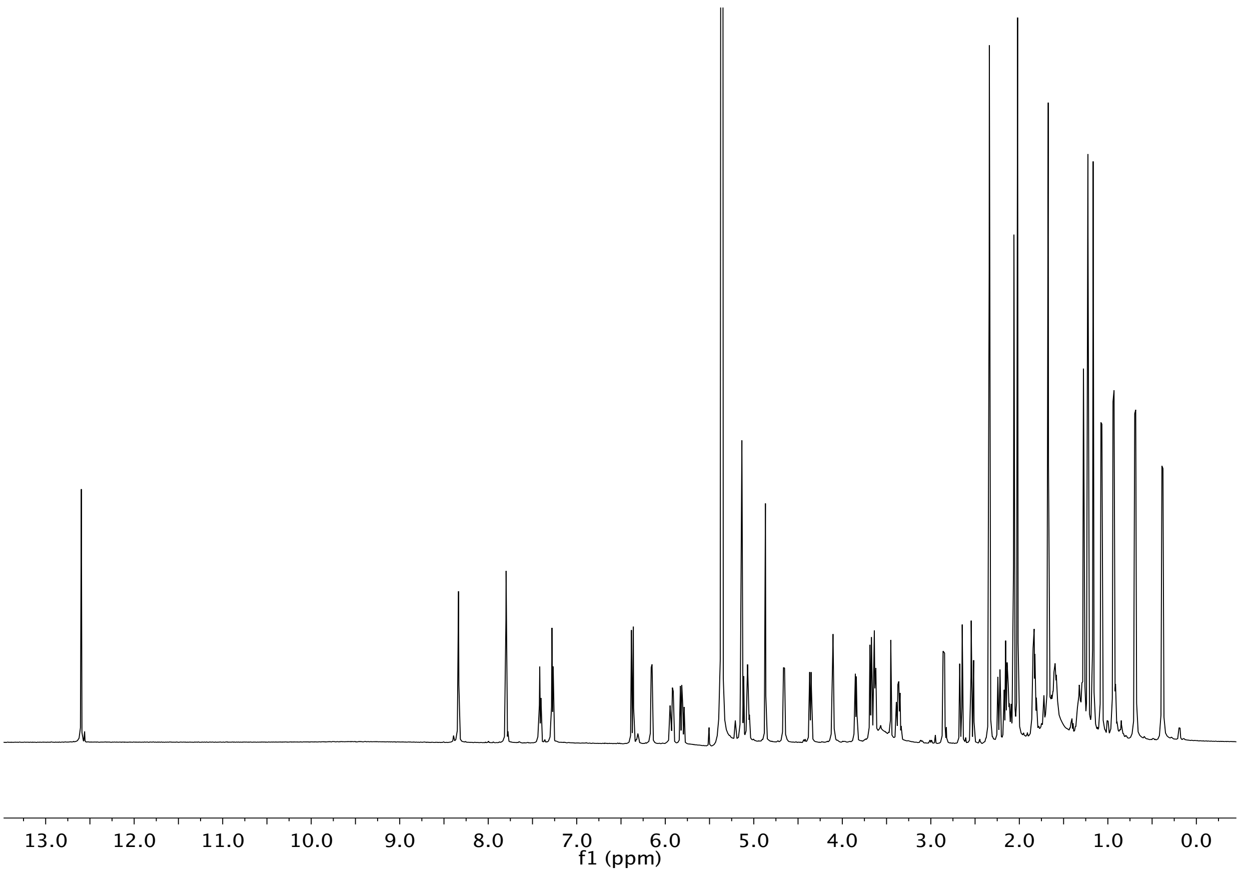

Supplementary Figure 6. ^{1}H NMR spectrum of Kang A in CD_2Cl_2, collected at 25 °C.

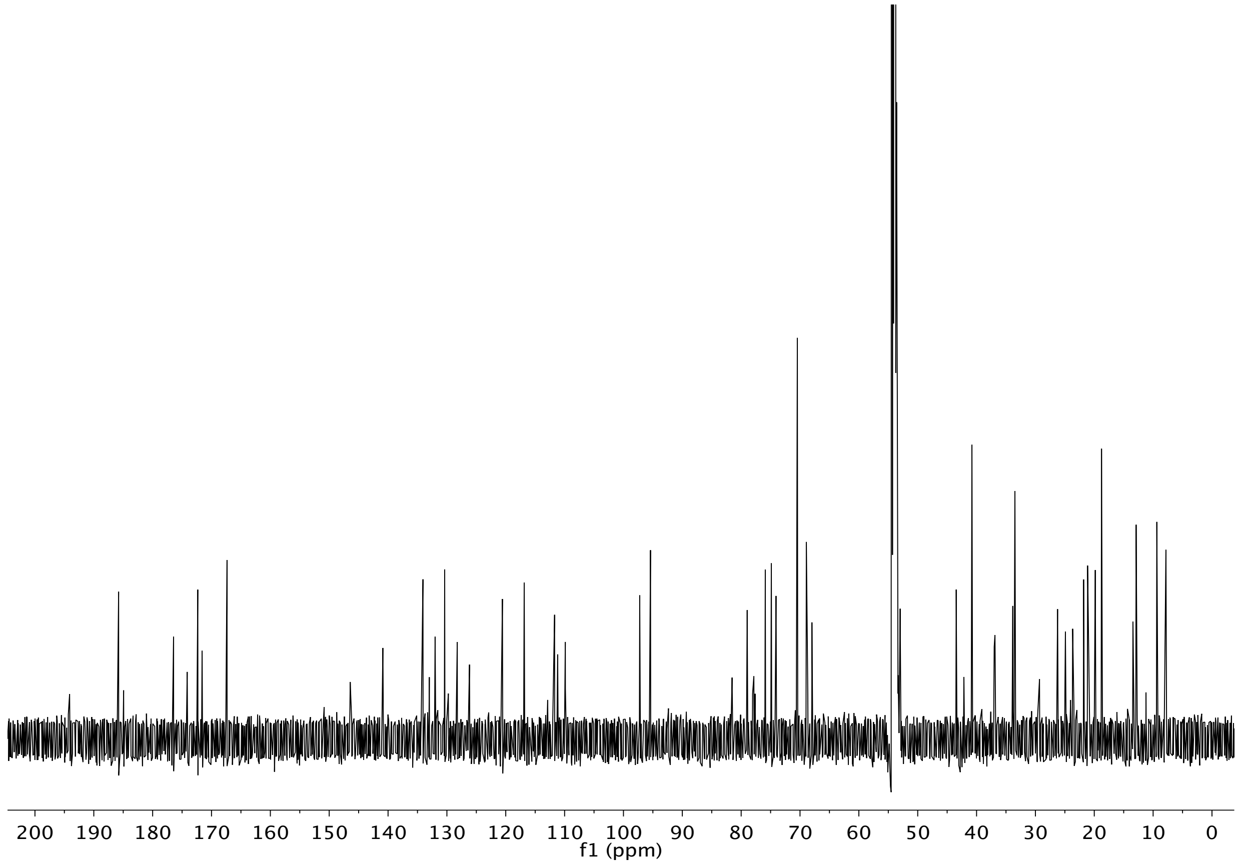

Supplementary Figure 7. ^{13}C NMR spectrum of Kang A in CD_2Cl_2, collected at 25 °C.

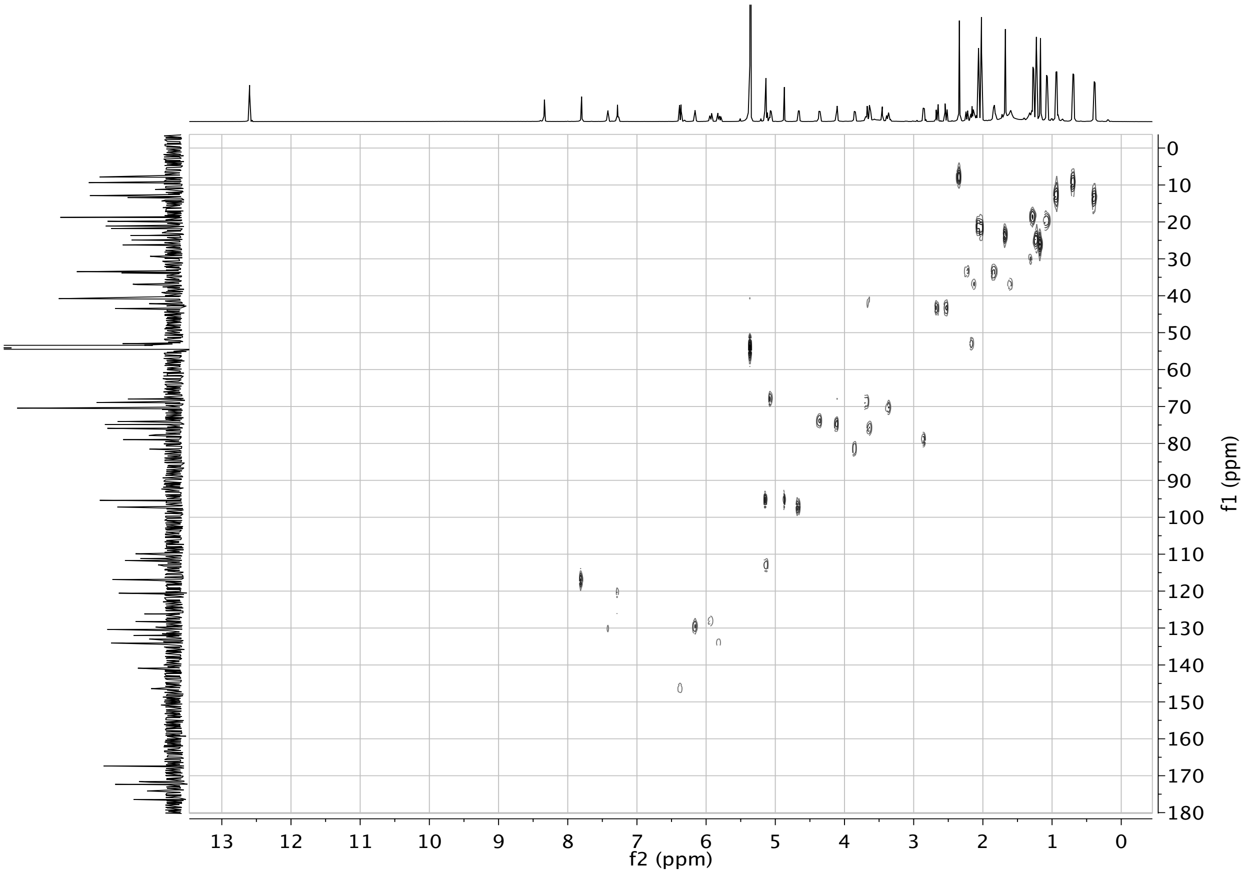

Supplementary Figure 8. HMQC NMR spectrum of Kang A in CD_2Cl_2, collected at 25 °C.

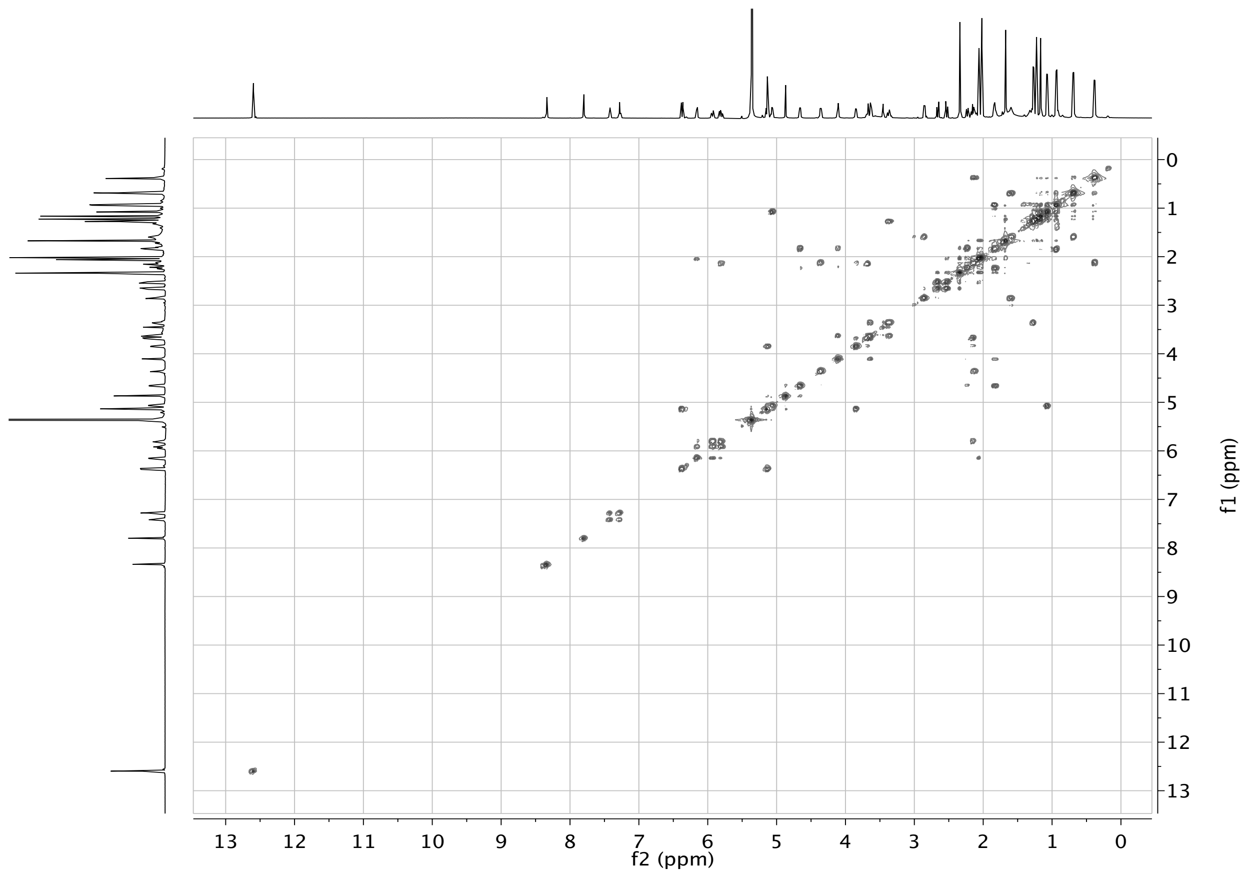

Supplementary Figure 9. COSY NMR spectrum of Kang A in CD_2Cl_2, collected at 25 °C.

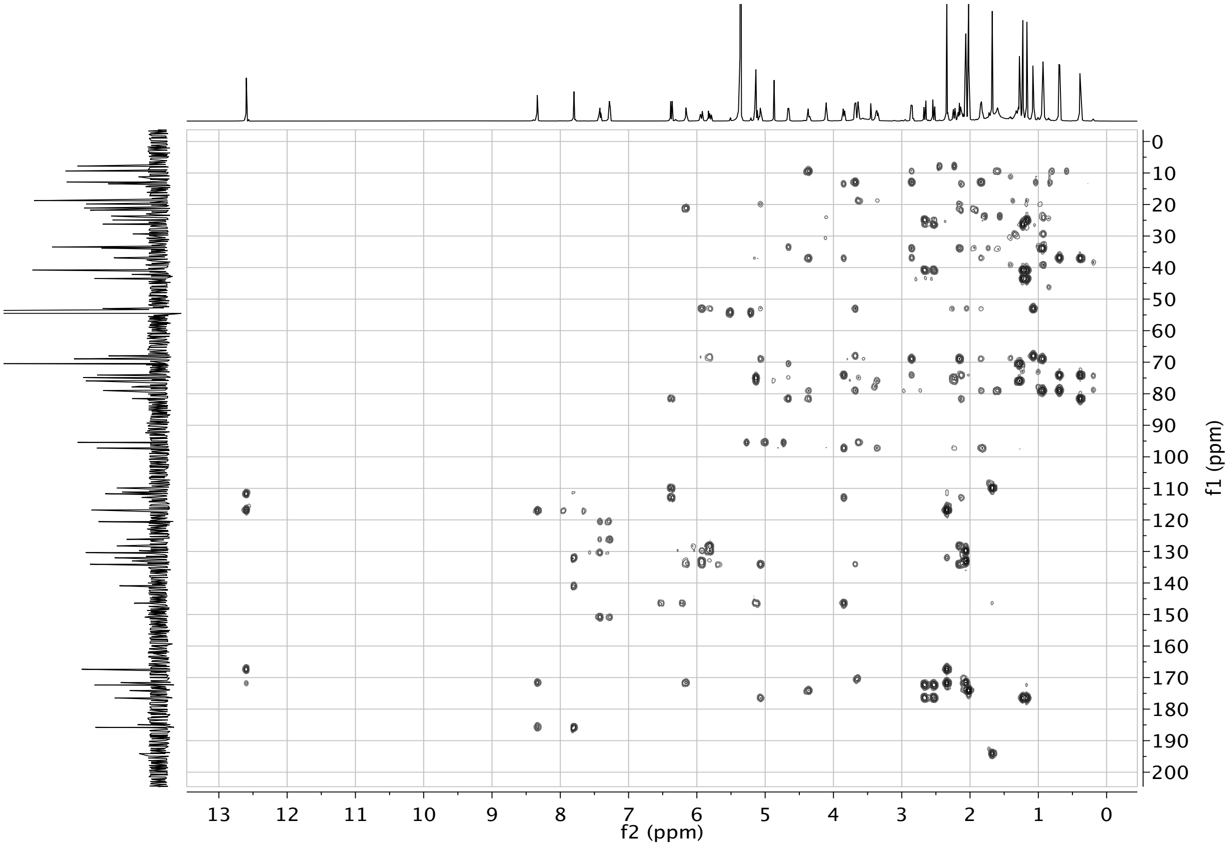

Supplementary Figure 10. HMBC spectrum of Kang A in CD_2Cl_2, collected at 25 °C.

HRMS analysis
Molecular formula
$C_{50}H_{65}NO_{19}Na$ (M+Na^{+})
Calculated *m/z*
1006.4048
Found *m/z*
1006.4006

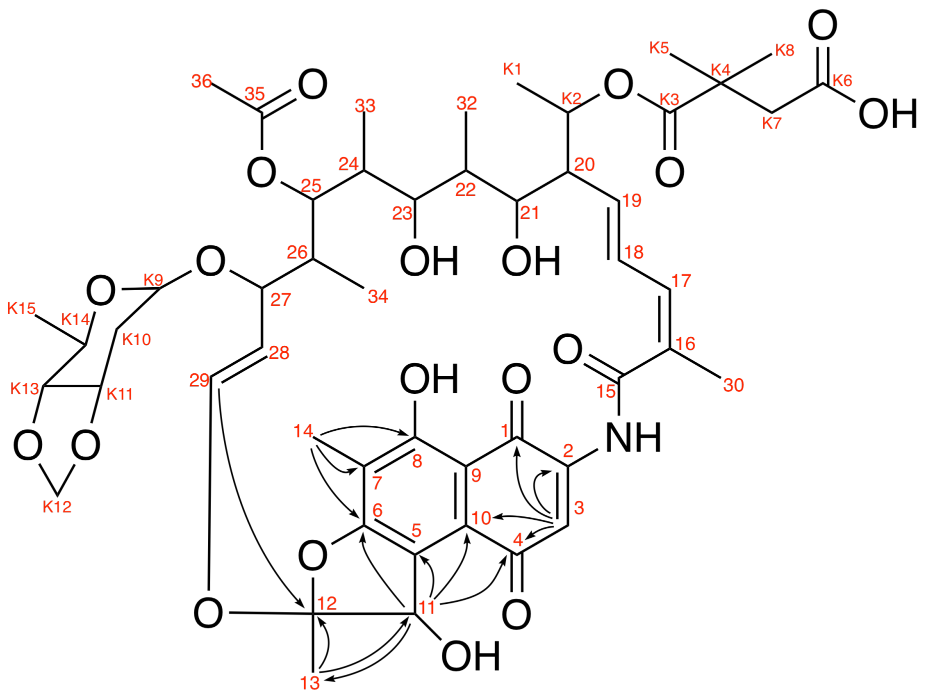

Supplementary Figure 11. NMR and HRMS data used to establish the structure of the Kang V1. The predicted molecular formula for Kang V1 [HRESIMS calcd *m/z* for $C_{50}H_{65}NO_{19}Na$ (M+Na^{+}) 1006.4048, found *m/z* 1006.4006], suggested it was a reduced analog of Kang A. A comparison of ^{1}H and ^{13}C NMR data from Kang A and Kang V1 suggested that this reduction was associated with the conversion of the C-11 ketone in Kang A [δ 194.1] to an alcohol [δ 77.1] in Kang V1. HMBC correlations from the Kang V1 C-11 proton [δ 5.49 (1H, s)] to C-4 [δ 188.8], C-5 [δ 109.4], C-6 [δ 164.9], and C-10 [δ 127.1] of the naphthoquinone and to C-13 [δ 25.0], as well as an HMBC correlation from the C-13 methyl protons [δ 1.86 (3H, s)] to C-11, confirmed that the highly deshielded C-11 ketone observed in the Kang A structure had been replaced by an alcohol in Kang V1. The remaining portions of the Kang V1 structure could be assigned using the same NMR arguments used to assign the structure of Kang A.

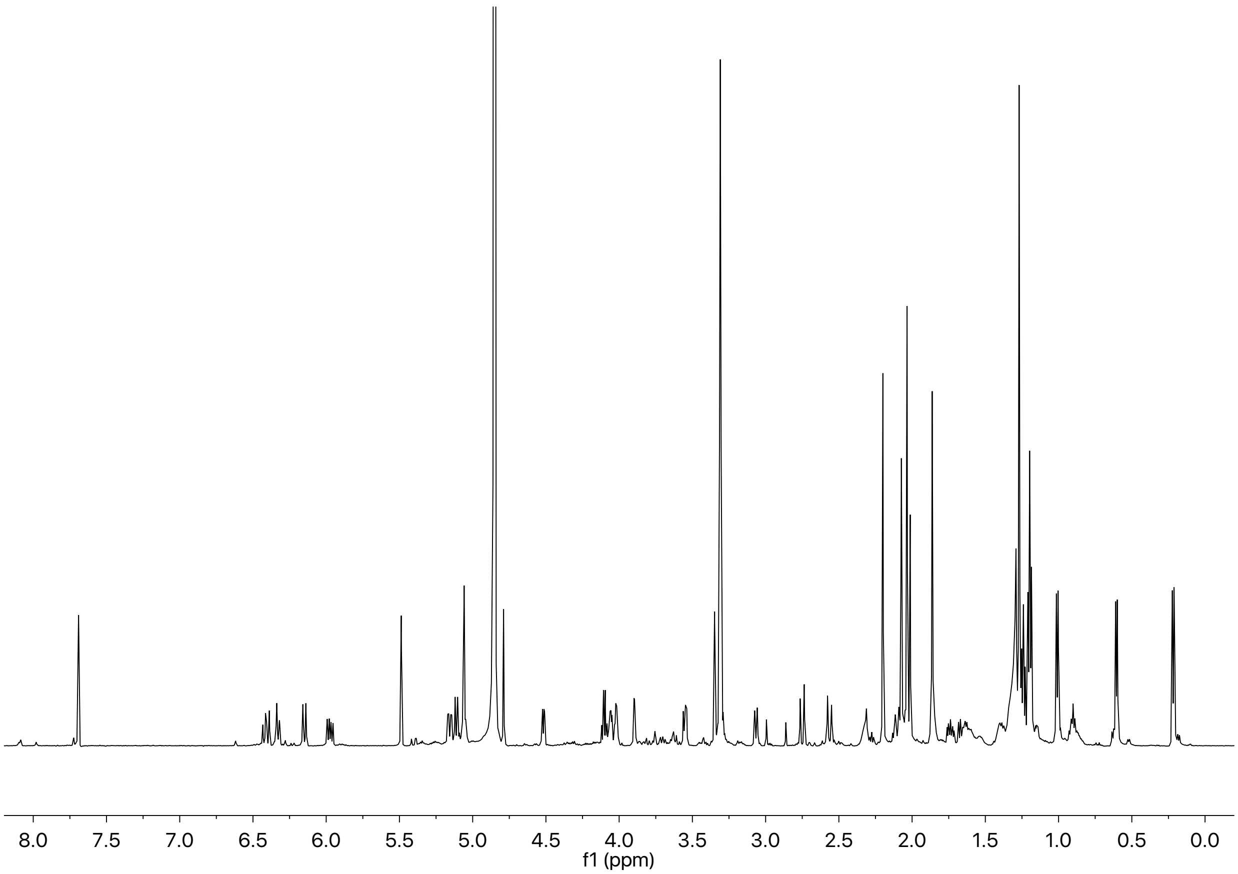

Supplementary Figure 12. ^{1}H NMR spectrum of Kang V1 in CD_3OD, collected at 25 °C.

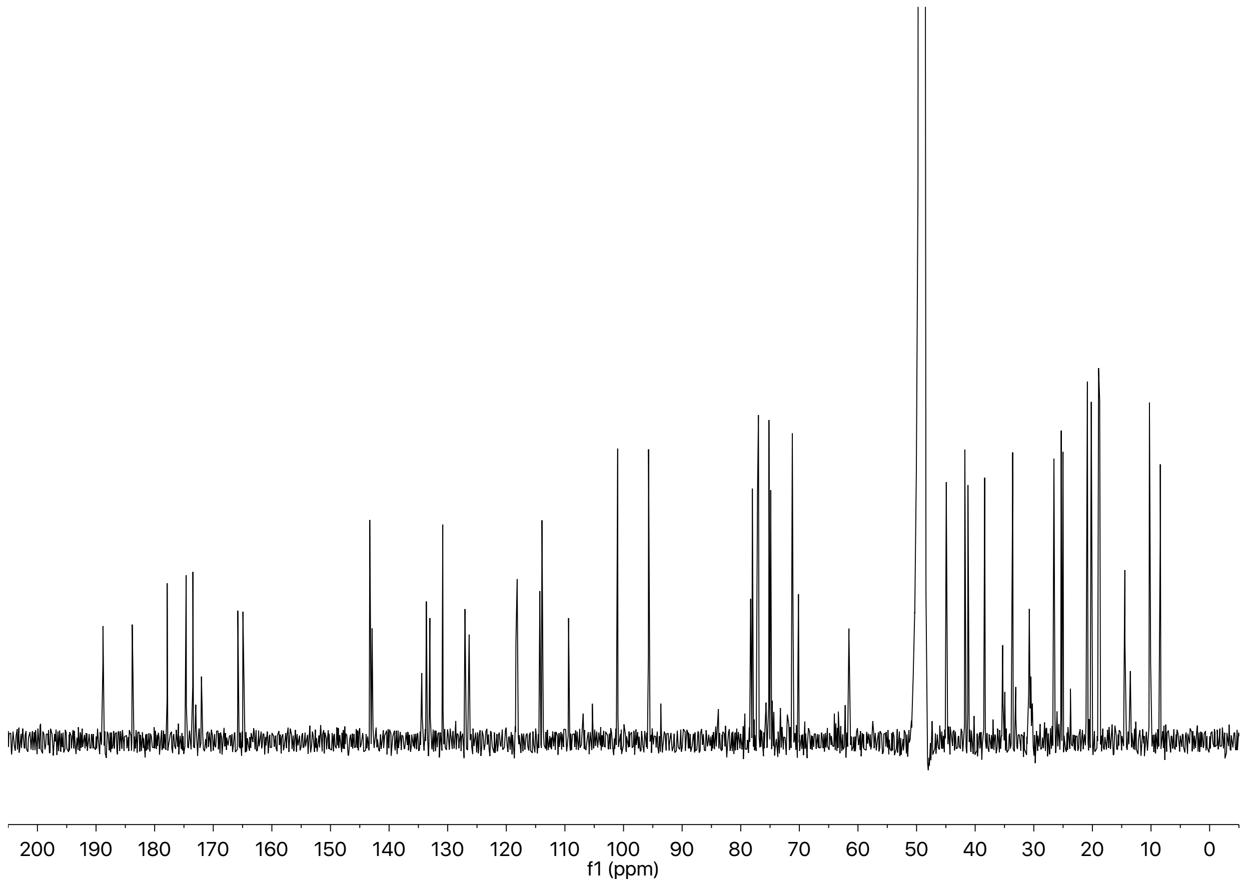

Supplementary Figure 13. **^{13}C NMR spectrum of Kang V1 in CD_3OD, collected at 25 °C.**

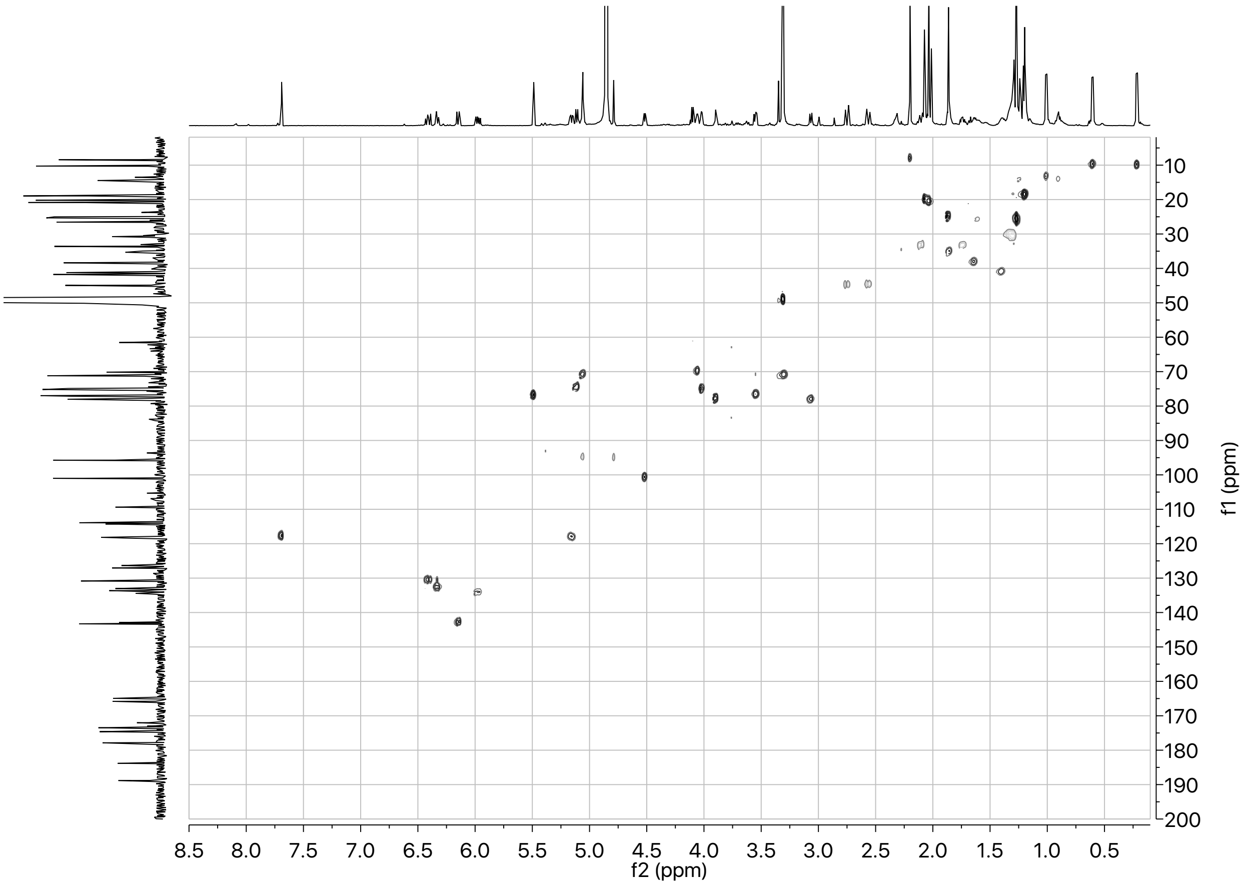

Supplementary Figure 14. HSQC NMR spectrum of Kang V1 in CD_3OD, collected at 25 °C.

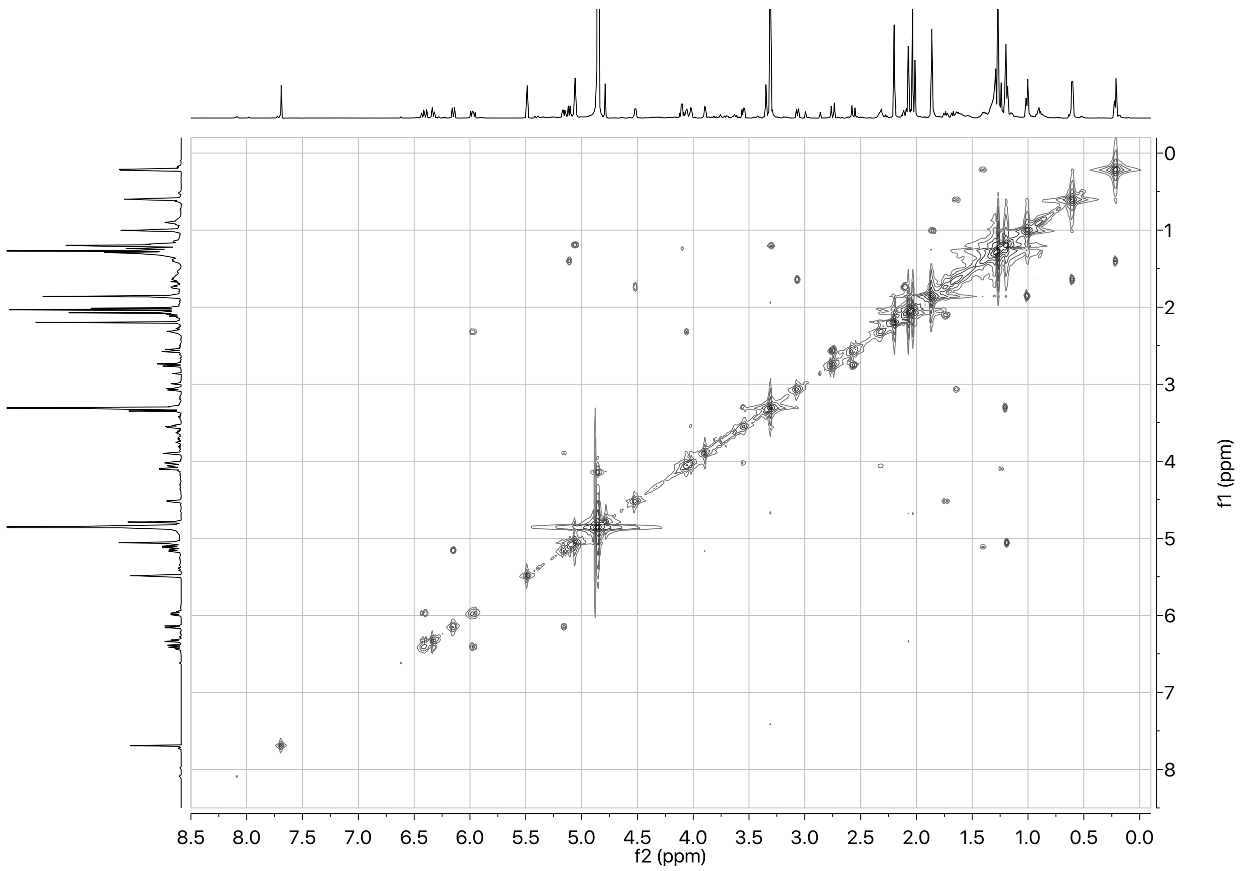

Supplementary Figure 15. COSY NMR spectrum of Kang V1 in CD_3OD, collected at 25 °C.

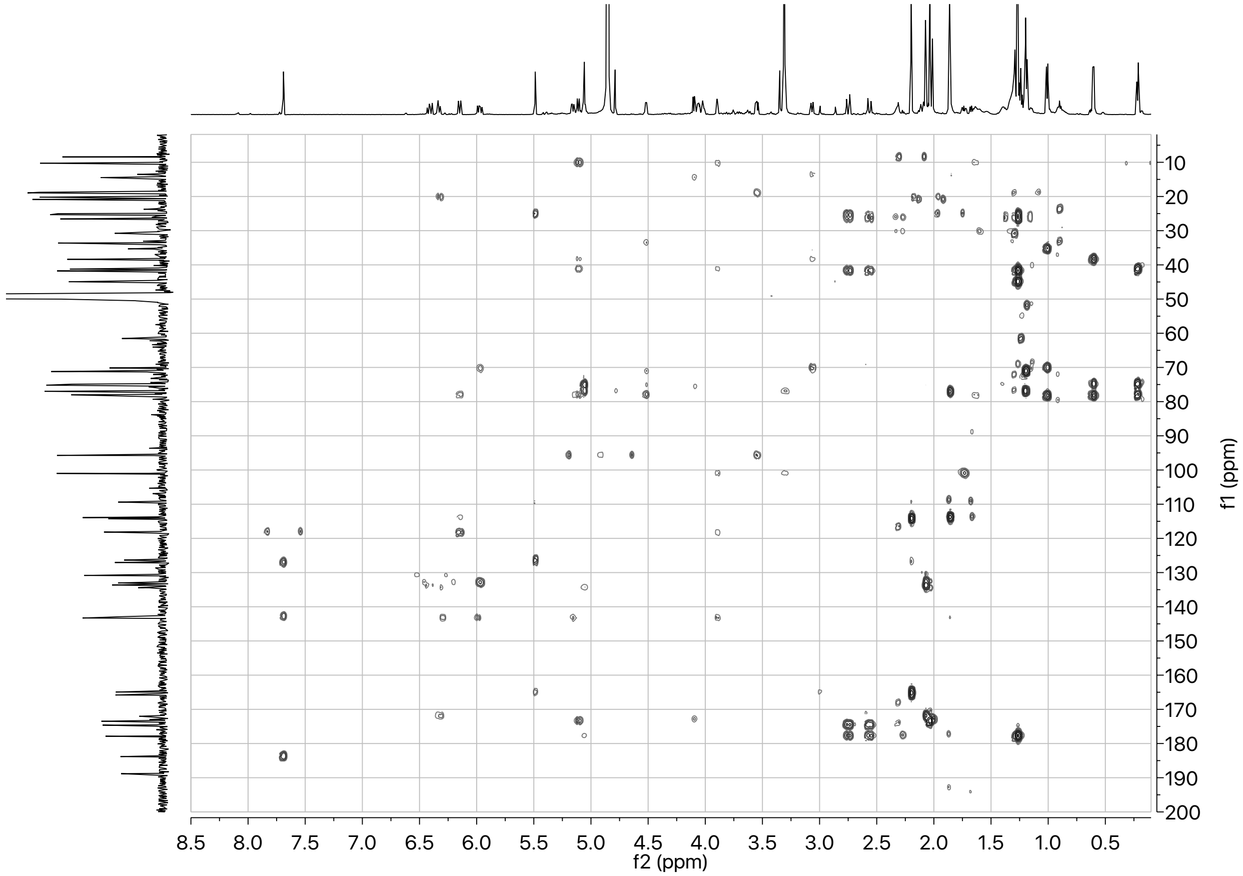

Supplementary Figure 16. HMBC spectrum of Kang V1 in CD_3OD, collected at 25 °C.

HRMS analysis

Molecular formula

$C_{51}H_{66}NO_{19}$ (M+H^{+})

Calculated *m/z*

996.4229

Found *m/z*

996.4197

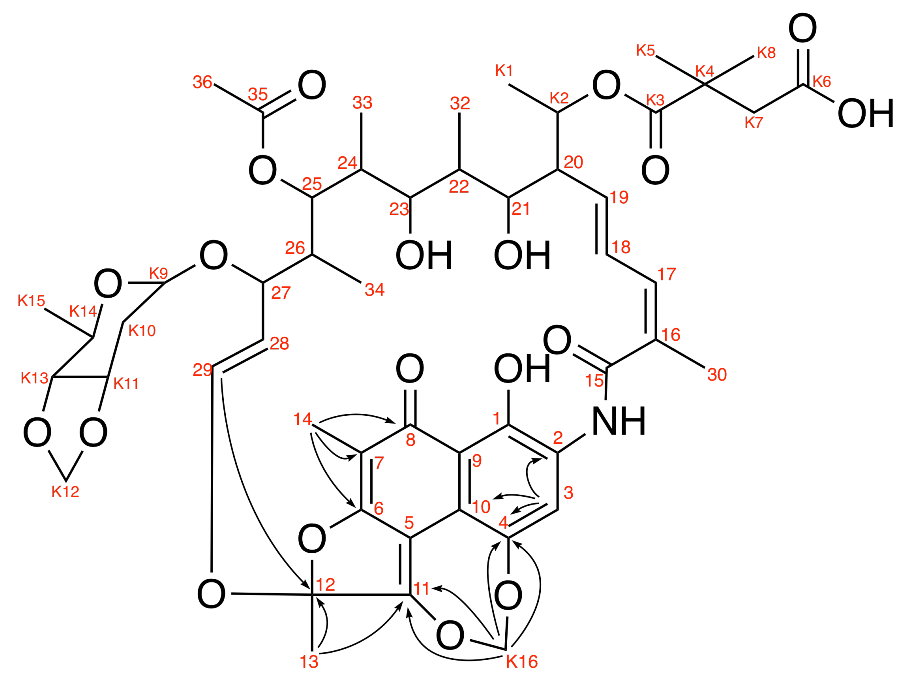

Supplementary Figure 17. NMR and HRMS data used to establish the structure of the Kang V2. HRMS data suggested that Kang V2 differed from Kang A by the addition of a CH_2 moiety [HRESIMS calcd *m/z* for $C_{51}H_{66}NO_{19}$ (M+H^{+}) 996.4229, found *m/z* 996.4197]. The UV spectra of Kang V2 supported the presence of a naphthohydroquinone moiety (λ_{max} 302 nm) instead of the naphthoquinone (λ_{max} 276) seen in Kang A and V1 (Supplementary Figure 4). The naphthohydroquinone substructure was also supported by HMBC correlations from H-3 [δ 7.99 (1H, s)] to carbons in the naphthohydroquinone substructure including a carbon at δ 150.5 ppm (C-4). In Kang A and V1, this carbon is significantly more deshielded [δ 184.9 and 188.8, respectively]. The presence of the carbonyl at C-8 in Kang V2 was supported by an HMBC correlation from the C-14 methyl protons [δ 2.01 (3H, s)] to the highly deshielded C-8 [δ 191.7]. The formation of a fourth ring off of the naphthohydroquinone substructure through the addition of a highly deshielded methylene [^{13}C δ 98.4, ^{1}H δ 6.19 (1H, d), δ 5.48 (1H, d)] was defined by HMBC correlations from the new methylene protons to both C-4 [δ 150.5] and C-11 [δ 165.7]. With the exception of the chemical shifts associated with the naphthohydroquinone substructure, the same NMR arguments used to assign the structures of Kang A and V1 could be used to assign the remaining regions of the Kang V2 structure.

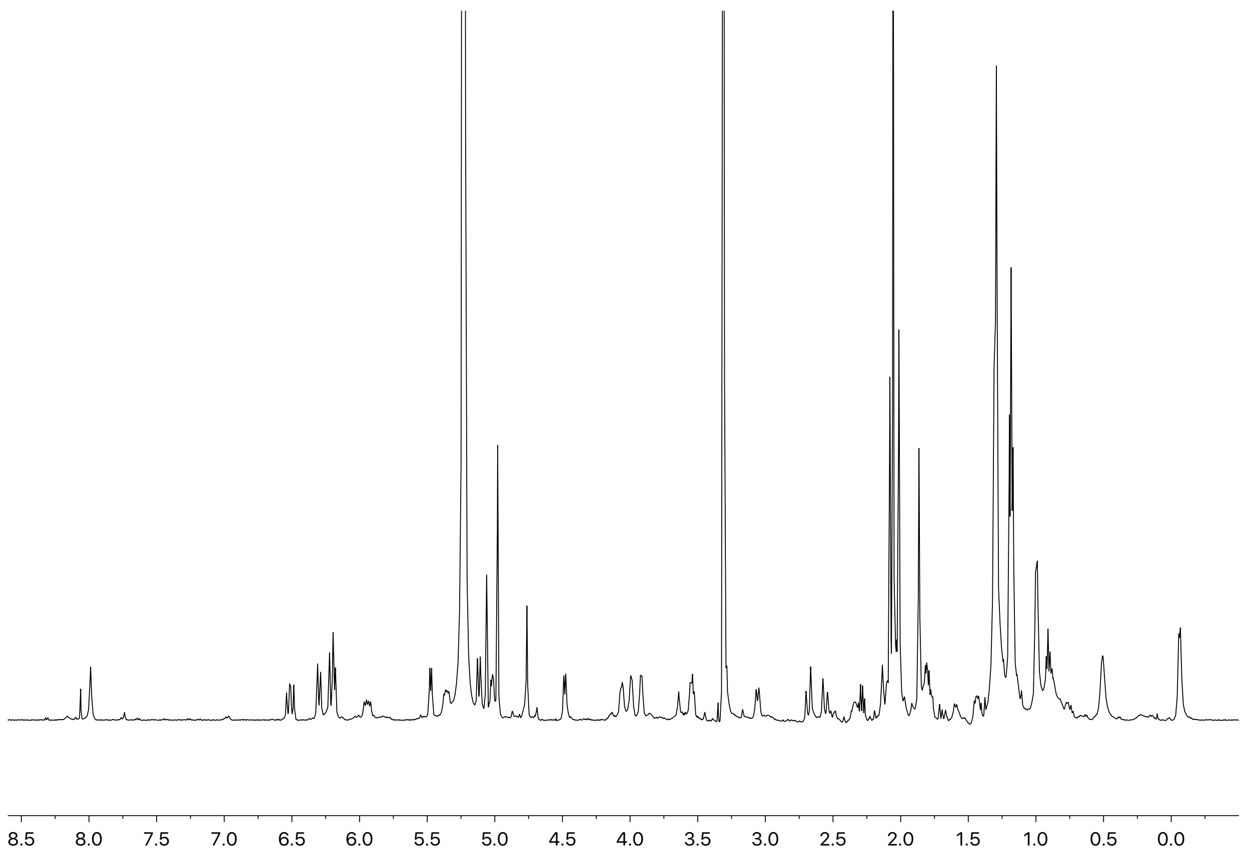

Supplementary Figure 18. ^{1}H NMR spectrum of Kang V2 in CD_3OD, collected at -20 °C.

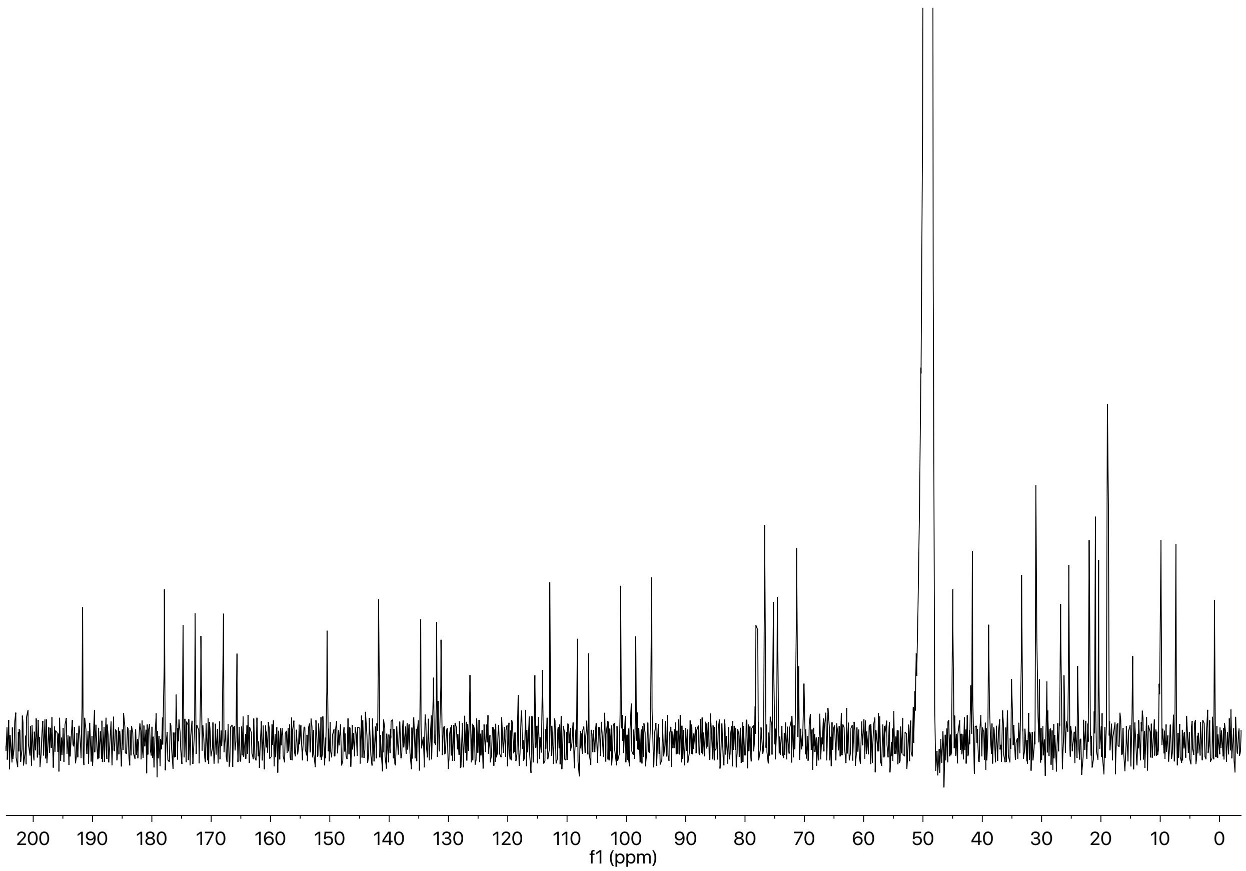

Supplementary Figure 19. ^{13}C NMR spectrum of Kang V2 in CD_3OD, collected at -20 °C.

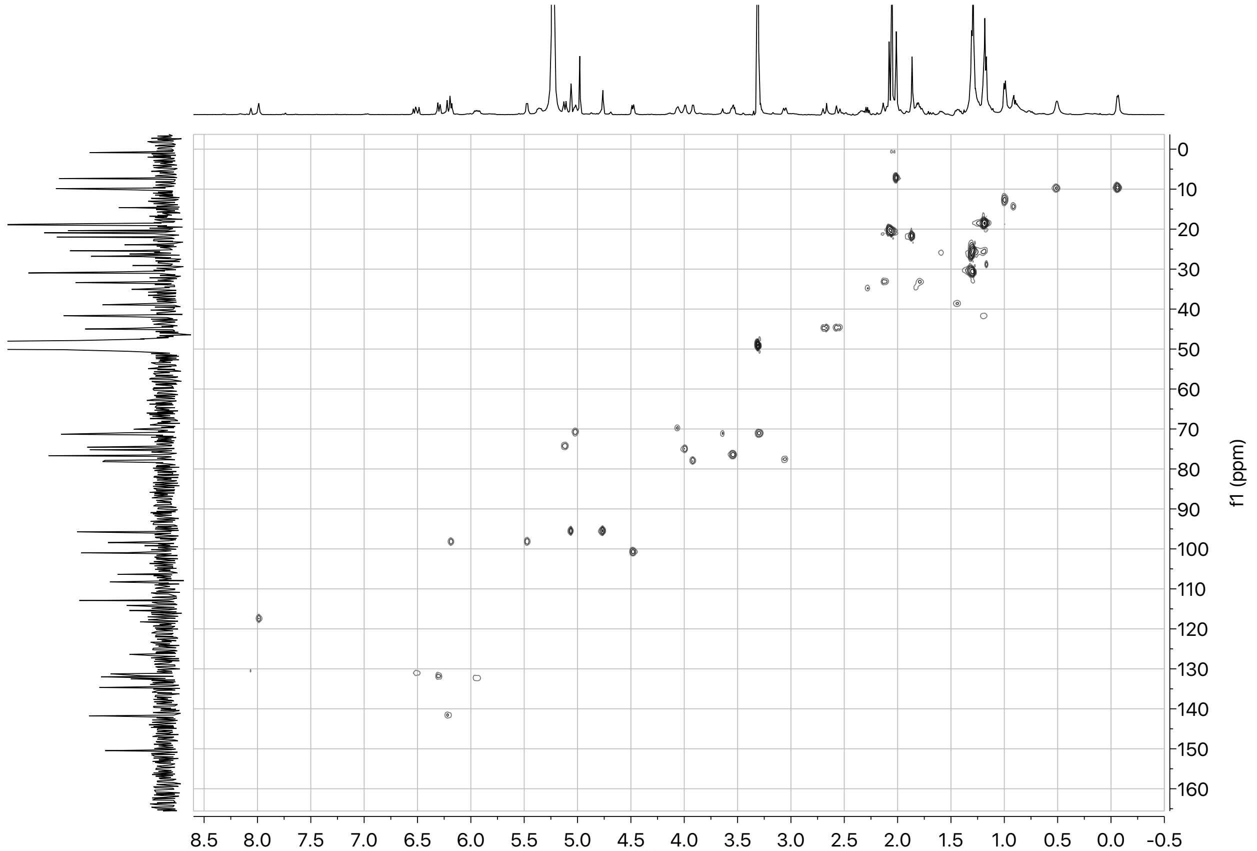

Supplementary Figure 20. HSQC NMR spectrum of Kang V2 in CD_3OD, collected at -20 °C.

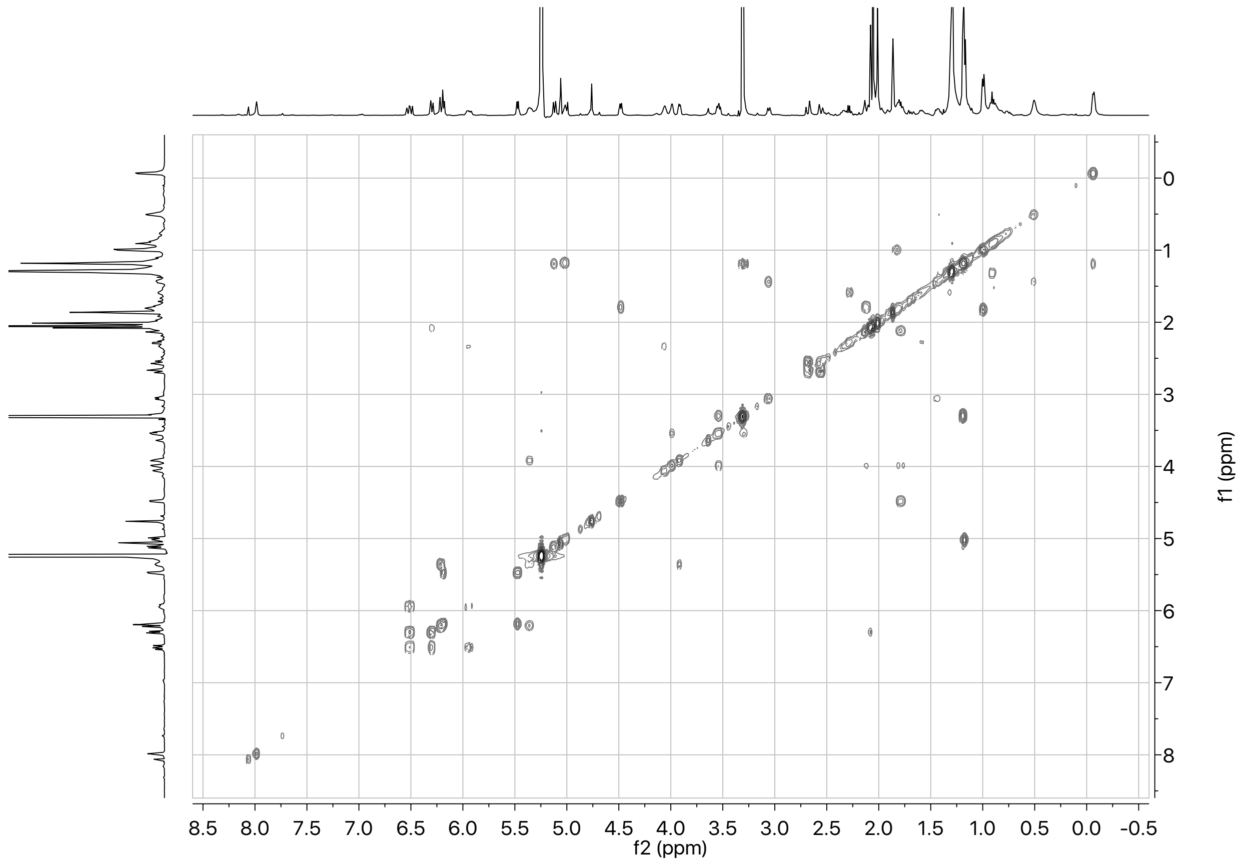

Supplementary Figure 21. COSY NMR spectrum of Kang V2 in CD_3OD, collected at -20 °C.

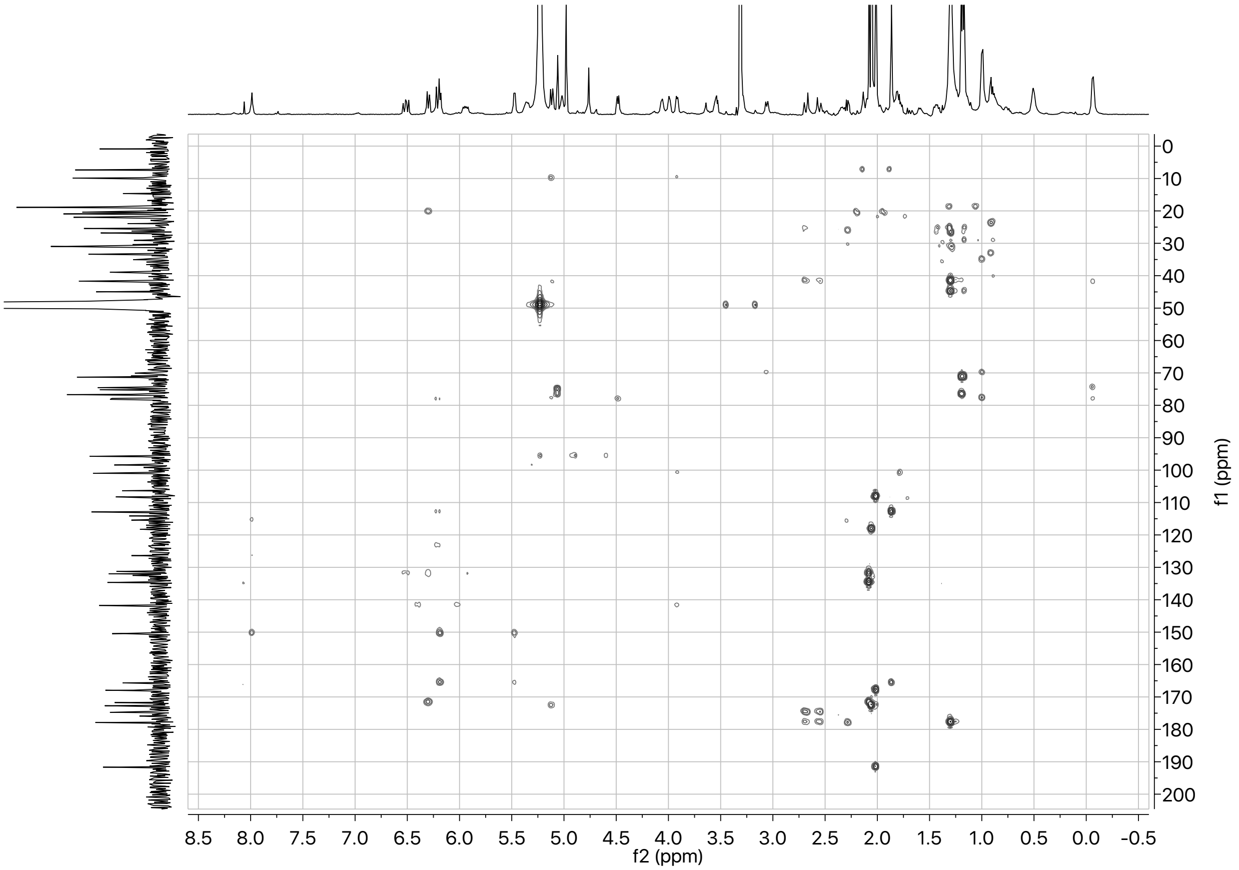

Supplementary Figure 22. HMBC spectrum of Kang V2 in CD_3OD, collected at -20 °C.

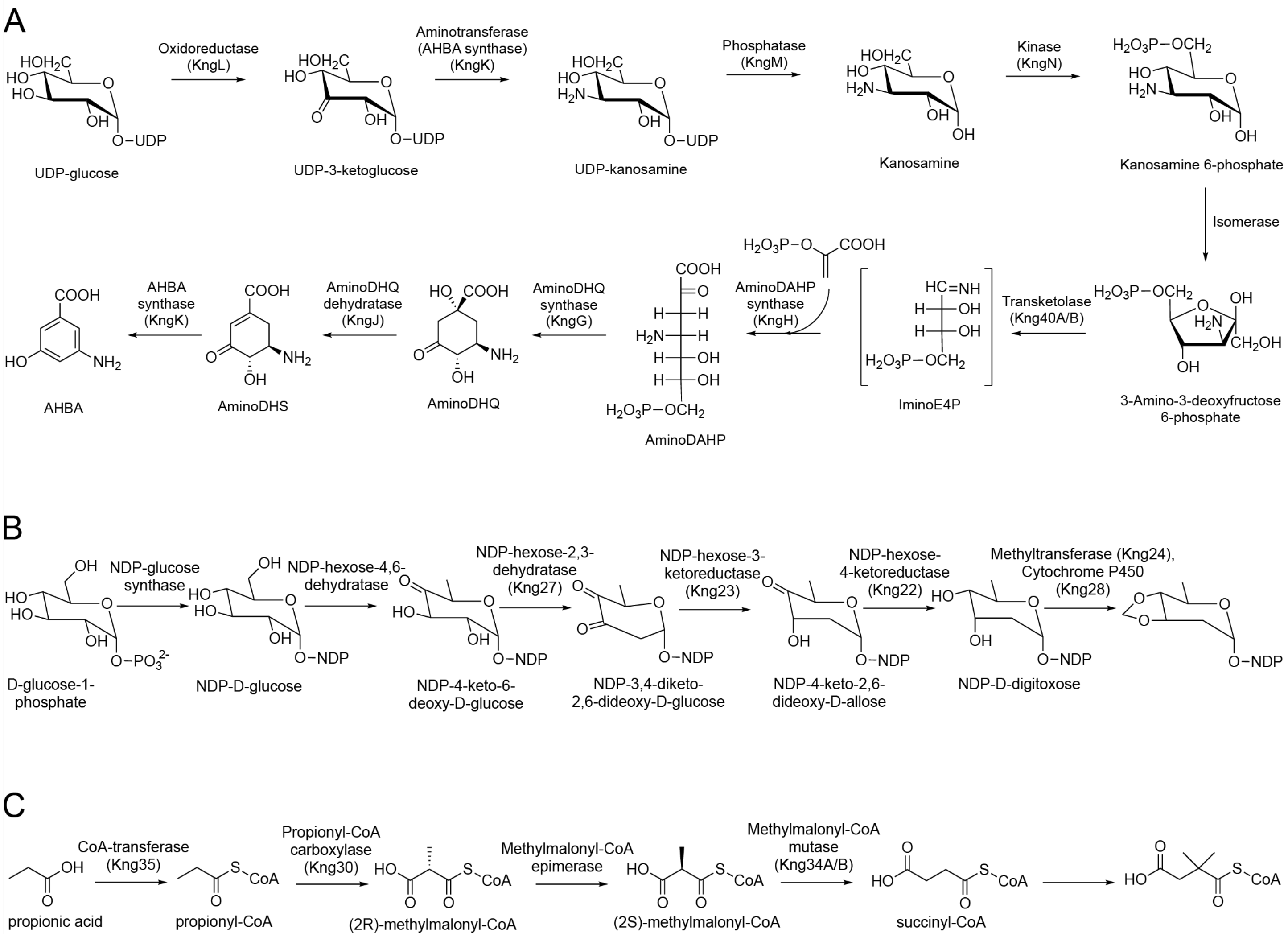

Supplementary Figure 23. Proposed biosynthesis of 3-amino-5-hydroxybenzoic acid (AHBA) and the K-sugar and K-acid moieties in *Amycolatopsis vancoresmycina*. A. Proposed biosynthesis of AHBA. **B.** Proposed biosynthesis of NDP-methylene digitoxose using the NDP-hexose-4-ketoreductase (*kng22*), NDP-hexose-3-ketoreductase (*kng23*), *O*-methyltransferase (*kng24*), NDP-hexose-2,3-dehydratase (*kng27*) and cytochrome P450 (*kng28*) genes from the *kng* cluster. The K-sugar is predicted to be appended to the polyketide core by the glycosyltransferase encoded by *kng26.* **C.** Proposed biosynthesis of 2,2-dimethylsuccinyl-CoA. While the predicted propionyl-CoA carboxylase (*kng30*), MmaI mutase (*kng34A/B*) and CoA transferase (*kng35*) genes found in the *kng* gene cluster are likely involved in generating a succinic acid moiety, the biosynthetic origin of a gem-dimethyl succinic acid has not been described previously and additional experiments will be required to better understand the origin of this functionality in the Kangs. Abbreviations: UPD, uridine diphosphate; iminoE4P, 1-deoxy-1-imino-D-erythrose 4-phosphate; aminoDAHP, 3,4-dideoxy-4-amino-D-arabino-heptulosonate 7-phosphate; aminoDHQ, 5-deoxy-5-amino-3-dehydroquinate; aminoDHS, 5-amino-5-deoxy-3-dehydroshikimate.

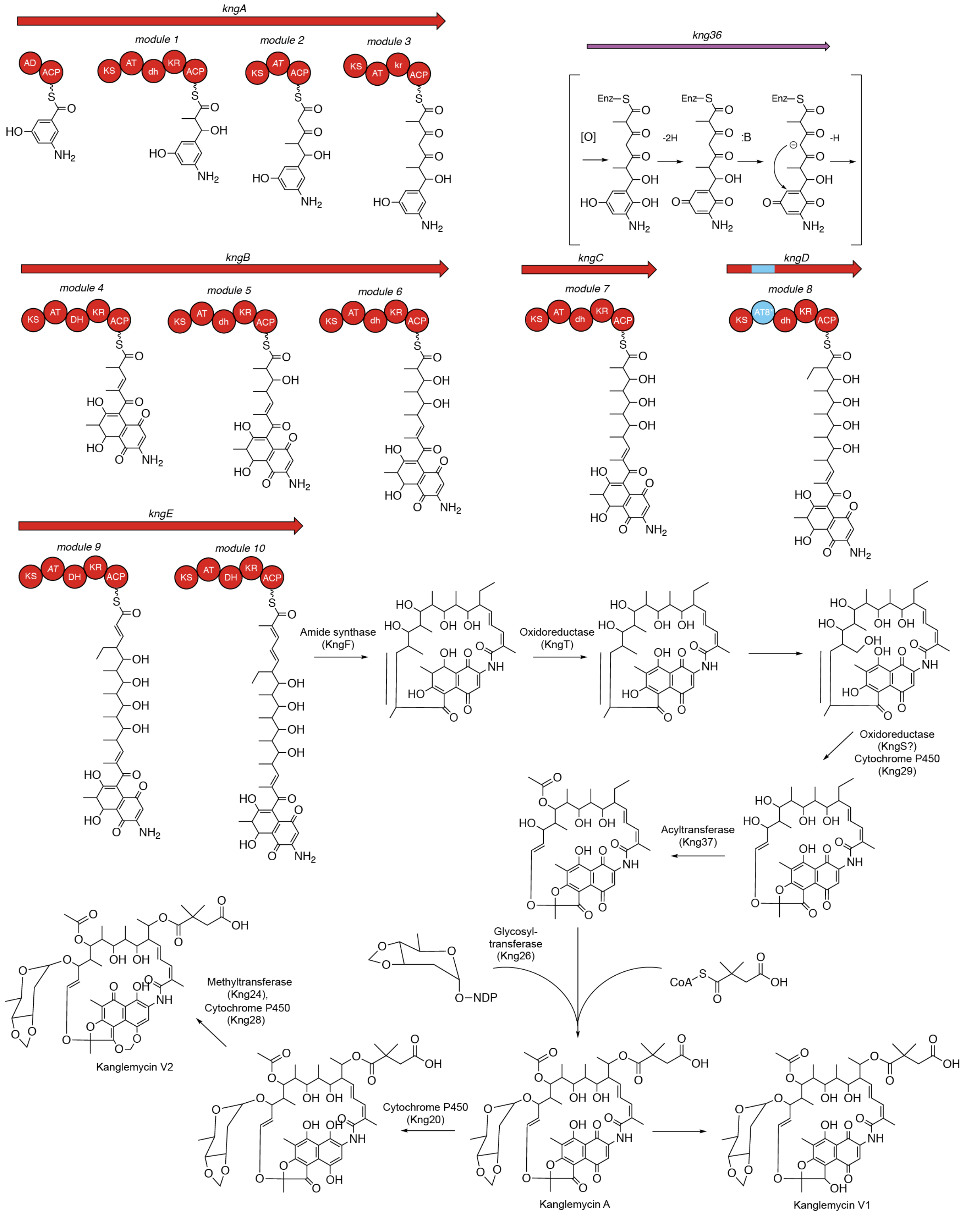

Supplementary Figure 24. Proposed biosynthesis and tailoring of the Kang polyketide core in *A. vancoresmycina*. Polyketide synthase domains that are predicted to be non-functional based on the final structures of the Kangs are shown in lower case letters. The origin of the methylenedioxy bridge in Kang V2 is not obvious from a bioinformatic analysis; however, one potential route is the repeated use of the methyltransferase (encoded by *kng24*) and cytochrome P450 (*kng28*) that we predict are responsible for introducing this functionality into the digitoxose sugar. Abbreviations: AD, adenylation domain; ACP, acyl carrier protein, KS, ketosynthase; *AT*, malonyl-CoA specific acyltransferase; AT8*, ethylmalonyl-CoA specific acyltransferase (all other AT domains are predicted to utilize methylmalonyl-CoA), KR, ketoreductase; DH, dehydratase.

Supplementary Table 3. Gene annotations for the *kng* biosynthetic gene cluster from *A. vancoresmycina* and corresponding genes from the *A. mediterranei* rifamycin cluster.

Gene Name	Length [aa]	Putative Function [Homolog Sp.]	%id / %sm	Accession	*A. mediterranei* homolog (%id / %sm)
**kngS**	330	gfo/Idh/MocA family oxidoreductase [A. mediterranei]	87/92	WP_01322543.1	rifS (87/92)
**kngT**	255	gfo/Idh/MocA family oxidoreductase [A kentuckyensis]	82/88	WP_086852324.1	rifT (80/85)
**kng3**	83	hypothetical protein [A. mediterranei S699]	84/90	AAC01708.1	rif35 (84/90)
**kng4**	396	cytochrome P450 [A kentuckyensis]	92/95	WP_08682320.1	rif0 (91/94)
**kngA**	4758	polyketide synthase [A. mediterranei]	90/93	WP_01322547.1	rifA (90/93)
**kngB**	5064	polyketide synthase [A kentuckyensis]	91/94	WP_08681069.1	rifB (90/93)
**kngC**	1810	polyketide synthase [Micromonospora nigra]	77/85	WP_09100512.1	rifC (77/83)
**kngD**	1824	polyketide synthase [Streptomyces subrutilus]	66/76	WP_06992302.1	rifD (64/72)
**kngE**	3448	polyketide synthase [A kentuckyensis]	78/85	WP_08681059.1	rifE (78/84)
**kngF**	258	N-acetyltransferase/amide synthase [A kentuckyensis]	85/91	WP_086841057.1	rifF (85/90)
**kng11**	62	hypothetical protein [A. mediterranei]	84/94	WP_013222553.1	rif1 (84/94)
**kngG**	351	aminoDHQ synthase [A kentuckyensis]	89/93	WP_086841053.1	rifG (87/90)
**kngH**	418	aminoDAHP synthase [Actinomadura formosensis]	81/89	WP_067800070.1	rifH (71/77)
**kngI**	270	shikimate dehydrogenase [A. mediterranei]	84/87	WP_013222556.1	rifI (84/87)
**kng15**	68	hypothetical protein, conserved [Trypanosoma equiperdum]	47/65	SCU70316.1	—
**kngK**	386	AHBA synthase [Streptomyces leeuwenhoekii]	84/91	WP_029387788.1	rifK (83/89)
**kngL**	357	gfo/Idh/MocA family oxidoreductase [Micromonospora rifamycinica]	79/86	WP_067307269.1	rifL (77/86)
**kngM**	232	phosphoglycolate phosphatase [Micromonospora halophytica]	88/93	WP_091293655.1	rifM (88/91)
**kngN**	294	kanosamine kinase [A kentuckyensis]	74/83	WP_086841040.1	rifN (70/81)
**kng20**	398	cytochrome P450 [Streptomyces leeuwenhoekii]	60/72	WP_029387780.1	rif4 (47/64)
**kng21**	1055	hypothetical protein (peptidase S41) [Streptomyces griseoplanus]	48/61	WP_055589579.1	—
**kng22**	253	NDP-hexose-4-ketoreductase [Streptomyces sp. NRRL S-350]	75/84	WP_030241082.1	—
**kng23**	353	NDP-hexose-3-ketoreductase [Streptomyces sp. NRRL S-350]	78/86	WP_030241085.1	—
**kng24**	255	NDP-hexose 3-O-methyltransferase [Streptomyces puniciscabiei]	87/94	WP_069778168.1	—
**kng25**	326	stationary phase survival protein SurE [A. mediterranei]	75/85	WP_013225304.1	—
**kng26**	383	dNTP-hexose glycosyl transferase [Actinomadura rifamycini]	68/81	WP_084550726.1	rif7 (60/73)
**kng27**	474	NDP-hexose 2,3-dehydratase [Plantactinospora sp. KBS50]	60/74	WP_095567734.1	rif18 (55/68)
**kng28**	378	cytochrome P450 [Streptomyces]	55/71	WP_052857194.1	—
**kng29**	421	cytochrome P450 [A. mediterranei]	81/89	WP_013222567.1	rif5 (81/89)
**kng30**	531	propionyl-CoA carboxylase [Lechevalieria aerocolonigenes]	71/82	WP_030473366.1	—
**kng31**	665	copper oxidase [Micromonospora halophytica]	71/81	WP_091298550.1	—
**kng32**	589	ABC transporter ATP-binding protein [Glycomyces tenuis]	62/78	WP_081687411.1	—
**kng33**	594	ABC transporter ATP-binding protein [Stackebrandtia nassauensis]	62/79	WP_013021481.1	—

**kng34A**	525	methylmalonyl-CoA mutase [Streptomyces fulvoviolaceus]	87/94	WP_030598014.1	—
**kng34B**	131	cobalamin B12-binding domain-containing protein [Frankia sp. EI5c]	84/96	WP_066061192.1	—
**kng35**	344	crotonobetainyl-CoA:carnitine CoA-transferase CaiB [Streptomyces olivochromogenes]	83/92	WP_067374600.1	—
**kng36**	484	3-(3-hydroxyphenyl)propionate hydroxylase [A kentuckyensis]	85/89	WP_086852066.1	rif19 (85/88)
**kng37**	392	acyltransferase [A. mediterranei]	83/90	WP_013222577.1	rif20 (83/90)
**kngR**	245	thioesterase [A. mediterranei]	90/94	WP_013222578.1	rifR (90/94)
**kng39**	422	cytochrome P450 [A rifamycinica]	90/95	WP_084093545.1	rif13 (90/94)
**kng40A**	231	transketolase [A tolypomycina]	82/88	WP_091304035.1	rif15A (80/86)
**kng40B**	303	transketolase [A rifamycinica]	87/91	WP_084093546.1	rif15B (87/91)
**kng41**	419	cytochrome P450 [A. mediterranei]	79/89	WP_014466580.1	rif16 (79/89)
**kngJ**	149	aminoDHQ synthase [A rifamycinica]	91/94	WP_043781867.1	rifJ (90/93)
**kng43**	425	LuxR family transcriptional regulator [A. mediterranei S699]	85/91	AEK39161.1	rif36 (85/91)

Supplementary Table 4. Antibacterial activity of Kangs A, V1, and V2.

Organism		Kang A MIC (μg mL^{-1})	Kang V1 MIC (μg mL^{-1})	Kang V2 MIC (μg mL^{-1})	Rifampicin MIC (μg mL^{-1})
Acinetobacter baumannii	ATCC17978	>64	>64	>64	ND
Enterococcus faecium	Com15	64	32	64	4.0
Listeria monocytogenes	ATCC15313	2.0	1.0	1.0	0.031
Proteus mirabilis	ATCC29906	>64	>64	>64	ND
Pseudomonas aeruginosa	PAO1	64	64	64	32
Salmonella enterica	IR715	>64	>64	>64	ND
Staphylococcus aureus	ATCC12600	0.62	0.069	0.62	0.023
Staphylococcus aureus	ATCC BAA-42	1.0	0.13	1.0	0.016
Staphylococcus aureus	ATCC BAA-1721	2.0	0.063	0.25	0.0078
Staphylococcus aureus	NCTC 8325 pT181	0.25	0.063	0.25	0.031
Staphylococcus aureus	USA100	1.0	0.13	1.0	0.0078
Staphylococcus aureus	USA200	2.0	0.25	2.0	0.0078
Staphylococcus aureus	USA800	0.5	0.063	1.0	0.016
Staphylococcus epidermidis	RP62A	0.25	0.031	0.25	0.016
		Kang A IC_{90} (μM)	Kang V1 IC_{90} (μM)	Kang V2 IC_{90} (μM)	Rifampicin IC_{90} (μM)
Mycobacterium tuberculosis	H37Rv	13	3.1	1.6	0.20

ND, not determined.

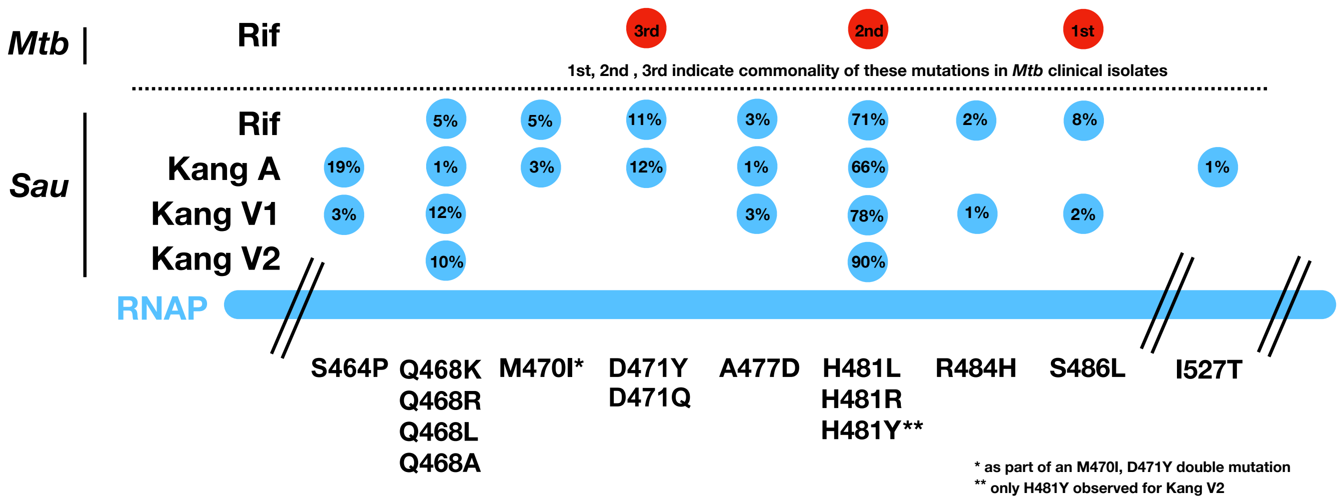

Supplementary Figure 25. Mutations found in RNAP (*rpoB*) genes sequenced from Rif^R or Kang^R *S. aureus*. A schematic of the *rpoB* gene is shown with the three most commonly mutated sites in Rif^R *Mtb* indicated with red circles. Rif^R and Kang^R mutations identified in *S. aureus* (this study) and their frequency are indicated with blue circles. *S. aureus* mutants were generated as follows. A saturated overnight culture of *S. aureus* grown in LB was used to inoculate 24x7 mL fresh LB cultures. The 24 new cultures were allowed to grow to an OD of ~1.1. A defined number of cells (10^9 cfu) from each of these 24 cultures was plated in a different well of a 24-well plate containing LB agar supplemented with either Rif, Kang A, Kang V1, or Kang V2 at 64x its respective MIC. 24 wells were plated for each compound. The plates were then incubated at 37 °C for 36 hours. Colony PCR was performed on 90-100 colonies from each plate using primers designed to amplify the Rif^R fragment of the *rpoB* gene (forward primer: 5'-CAGATGATATTGACCATTTAGGTAACCGTCGT-3'; reverse primer: 5'-GCTTGTGCTACAACATAGCTATCTTCTTCG-3'). PCR amplicons were Sanger sequenced to identify mutations.

Supplementary Table 5. Table of crystallographic statistics.[a]

	Msm TIC/Rif	*Msm* TIC/Kang A	*Msm* TIC (RNAP[β^{S447L}])/Kang A
**Data collection**			
Space group	$P2_1$	$P2_1$	$P2_1$
Combined datasets	1	1	1
Cell dimensions			
a (Å)	132.353	130.564	129.250
b (Å)	162.325	161.583	160.909
c (Å)	139.401	135.141	136.864
β (°)	107.370	110.734	110.747
Wavelength (Å)	0.97920	0.91806	0.97920
Resolution (Å)	55.00 – 3.05 (3.16 – 3.05)[b]	50.00 – 3.05 (3.16 – 3.05)[b]	45.00 – 3.45 (3.57 – 3.45)[b]
Total reflections	790,388 (55,184)	564,439 (52,088)	304,660 (26,885)
Unique reflections	106,159 (8,923)	99,721 (9,775)	68,062 (6,780)
Multiplicity	7.4 (5.4)	5.7 (5.3)	4.5 (4.0)
Completeness (%)	97.95 (83.36)	99.76 (98.57)	97.79 (98.78)
$\langle I \rangle/\sigma I$	13.86 (0.70)	8.42 (0.51)	8.02 (0.67)
Wilson B-factor (Å^2)	88.48	103.52	103.86
R_{merge}	0.1518 (2.285)	0.143 (2.227)	0.162 (1.63)
R_{meas}	0.163 (2.516)	0.157 (2.462)	0.183 (1.853)
R_{pim}	0.05852 (1.026)	0.0637 (1.032)	0.0827 (0.856)
CC1/2[c]	0.997 (0.503)	0.998 (0.31)	0.996 (0.381)
CC*[c]	0.999 (0.818)	0.999 (0.688)	0.999 (0.743)
**Refinement**			
R_{work} / R_{free}	0.224/0.268 (0.339/0.349)	0.232/0.262 (0.402/0.420)	0.252/0.290 (0.341/0.377)
CC_{work}/CC_{free}	0.939/0.894 (0.687/0.639)	0.938/0.930 (0.488/0.412)	0.921/0.912 (0.487/0.436)
No. atoms	26,534	25,357	23,210
Macromolecules	26,362	25,233	23,077
Ligand	59 (Rif)	70 (Kang A)	70 (Kang A)
Ions	2 (2 Zn^{2+})	2 (1 Mg^{2+}, 1 Zn^{2+})	3 (1 Mg^{2+}, 2 Zn^{2+})
Solvent components	37	61	70
Protein residues	3,331	3,174	2,945
B-factors			
Macromolecules	94.94	106.52	113.45
Ligand	75.05 (Rif)	101.18 (Kang A)	125.78 (Kang A)
Ions/solvent components	95.05	124.99	128.01
R.m.s deviations			
Bond lengths (Å)	0.002	0.004	0.002
Bond angles (°)	0.507	0.91	0.510
Clashscore	8.54	8.28	7.88
Ramachandran favored (%)	95.67	97.09	95.79
Ramachandran outliers (%)	0.24	0.06	0.35

[a] PDB validation reports are available at:

http://files.rcsb.org/pub/pdb/validation_reports/CC/6CCV/6CCV_full_validation.pdf
http://files.rcsb.org/pub/pdb/validation_reports/CC/6CCE/6CCE_full_validation.pdf
http://files.rcsb.org/pub/pdb/validation_reports/DC/6DCF/6DCF_full_validation.pdf

[b] Values in parentheses are for highest-resolution shell.

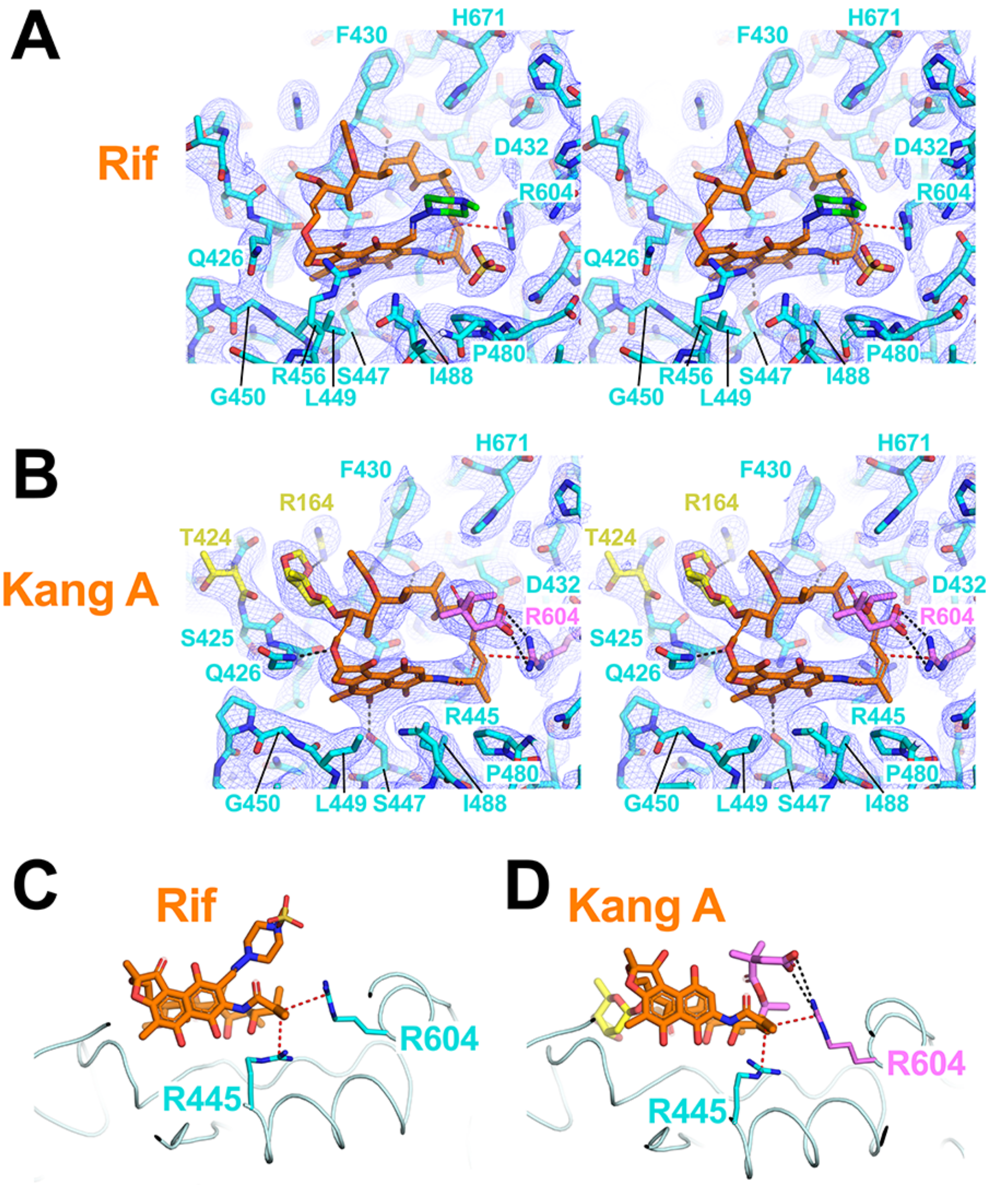

Supplementary Figure 26. Electron density and cation-π bridges. A. Stereo view of the Rif binding pocket in the *Msm* RNAP β subunit with the $2F_o$-F_c electron density map (blue mesh, 1.2 σ). Rif is colored orange with the piperazine group colored green. The RNAP β subunit is cyan. Polar interactions between the RNAP and Rif are denoted by dashed lines (cation- π interactions, red; H-bonds, gray). Selected residues that interact with Rif are labeled. **B.** Same as (A) but showing the Kang A binding pocket. **C.** The Rif binding pocket, viewed roughly orthogonal to the view of Supplementary Figure 26A. Rif is shown as in (A). The RNAP β subunit is shown as an α-carbon backbone worm (light cyan). The two side chains making the cation-π sandwich (denoted by red dashed lines) with Rif are shown (cyan). **D.** Same as (C) but showing Kang A. In addition to participating in the cation-π sandwich, R604 forms a salt bridge with K-acid (violet).

Kang A (S447L)

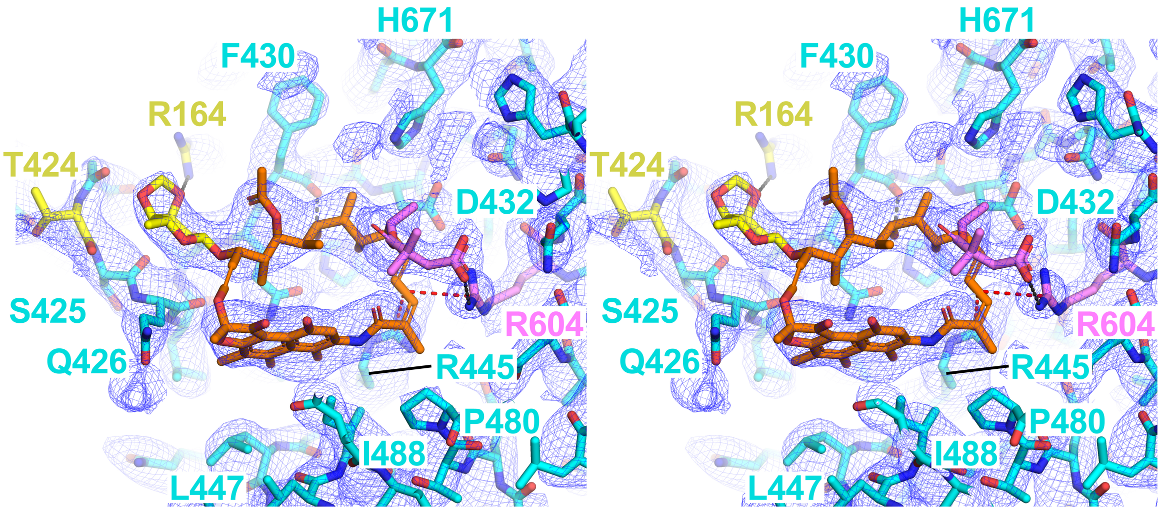

Supplementary Figure 27. Electron density of S447L RNAP with Kang A. Stereo view of the Kang A binding pocket in the *Msm* RNAP S447L β subunit with the $2F_o$-F_c electron density map (blue mesh, 1.2 σ). The Kang A/Rif PK backbone is colored orange with the K-sugar and K-acid colored yellow and violet, respectively. The RNAP β subunit is cyan. Polar interactions between the RNAP and Kang A are denoted by dashed lines (cation-π interactions, red; H-bonds, gray). Selected residues that interact with Kang A are labeled.

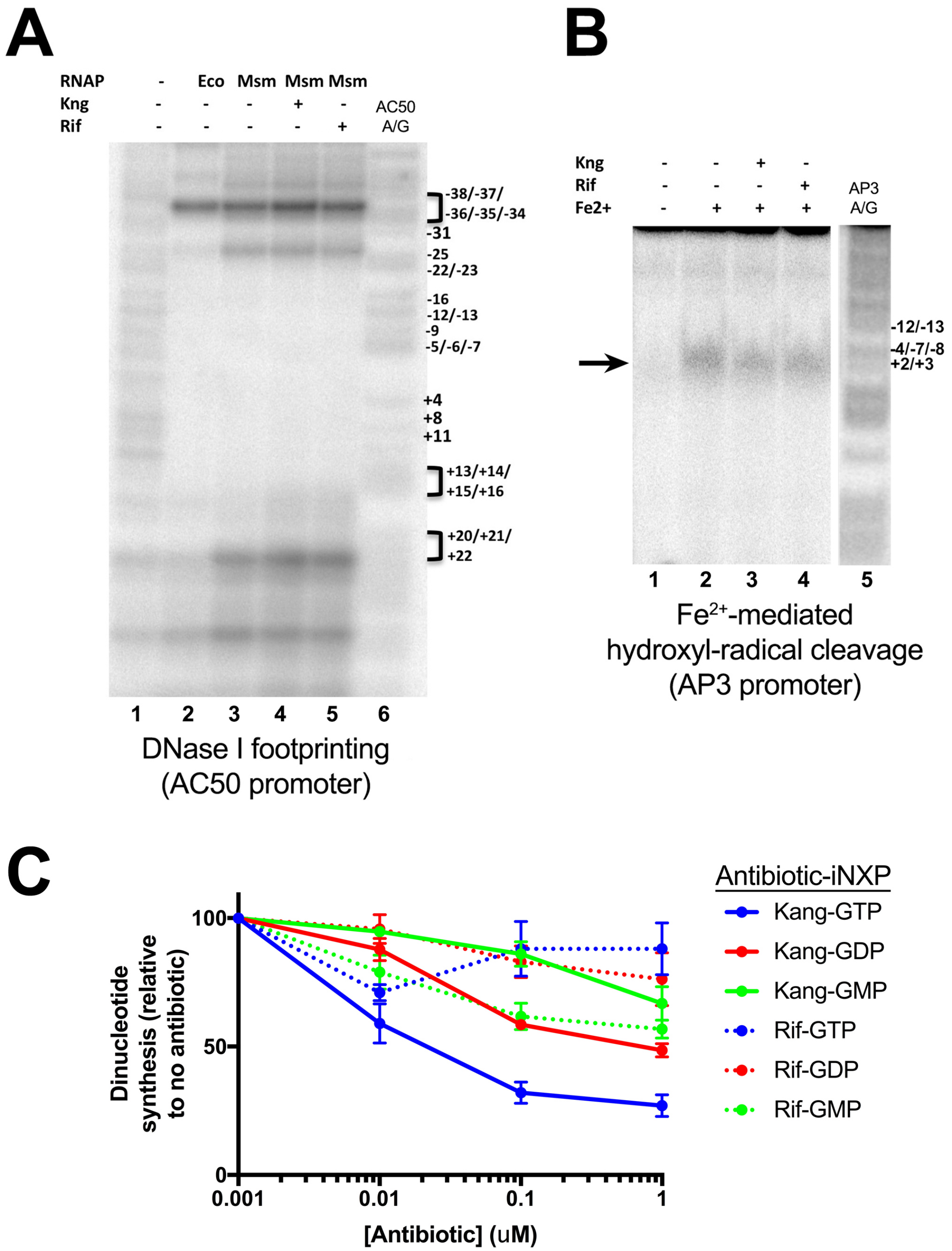

Supplementary Figure 28. RNAP footprint of promoter DNA in the presence of antibiotics. A. DNase I footprints (template strand) of *Eco* and *Msm* RNAPs on the AC50 promoter. Lanes: 1, DNase I cleavage in the absence of any proteins; 2, protection of DNA by *Eco* σ^{70}-holoenzyme; 3, protection by *Msm* σ^{A}-holoenzyme; 4, protection by *Msm* σ^{A}-holoenzyme in the presence of Kang A; 5, protection by *Msm* σ^{A}-holoenzyme in the presence of Rif. Lane 6, A/G sequencing ladder (assignments shown on the right). **B.** Fe^{2+}-mediated cleavage of the AP3 promoter in the presence of antibiotics. All lanes contained *Msm* σ^{A}-holoenzyme. Lanes: 1, no Fe^{2+} added; 2, no antibiotic in the presence of Fe^{2+}; 3, Kang A in the presence of Fe^{2+}; 4, Rif in the presence of Fe^{2+}. Lane 5: A/G sequencing ladder of AP3. Cleavage at +1 is indicated by arrow. **C.** Inhibition of dinucleotide synthesis by increasing concentrations of antibiotic (Kang or Rif) and with GTP (blue), GDP (red), or GMP (green) as the 5'-initiation nucleotide. The error bars denote standard error from four replicates.

Supplementary Table 6. Gene annotations for the metagenomic gene cluster RifCon12 and corresponding genes from the *A. vancoresmycina kng* cluster.

Gene Name	Length [aa]	Putative Function [Homolog Sp.]	%id / %sm	Accession	*A. vanc.* homolog (%id / %sm)
**orf2**	327	gfo/Idh/MocA family oxidoreductase [Micromonospora nigra]	84/91	WP_091080786.1	kngS (82/89)
**orf3**	322	gfo/Idh/MocA family oxidoreductase [Micromonospora nigra]	68/77	WP_091080781.1	kngT (46/55)
**orf4**	72	hypothetical protein [Actinomadura formosensis]	83/87	WP_084262634.1	kng35 (65/80)
**orf5**	397	cytochrome P450 [Actinomadura formosensis]	85/92	WP_067794733.1	kngR0 (83/91)
**orf6**	5225	polyketide synthase [Streptomyces albogriseolus]	72/79	AHD24374.1	kngA (75/82)
**orf7**	5181	polyketide synthase [Micromonospora halophytica]	80/87	WP_091298529.1	kngB (77/83)
**orf8**	1809	polyketide synthase [Streptomyces leeuwenhoekii]	78/85	WP_047121566.1	kngC (75/83)
**orf9**	1785	polyketide synthase [Streptomyces subrutilus]	71/80	WP_069922302.1	kngD (67/75)
**orf10**	3449	polyketide synthase [Micromonospora halophytica]	78/85	WP_091293672.1	kngE (75/83)
**orf11**	258	N-acetyltransferase/amide synthase [Actinomadura formosensis]	84/91	WP_084263055.1	kngF (74/84)
**orf12**	59	hypothetical protein [Micromonospora halophytica]	35/56	SCG46356.1	—
**orf13**	354	aminoDHQ synthase [Actinomadura formosensis]	90/95	WP_067800067.1	kngG (87/93)
**orf14**	392	aminoDAHP synthase [Actinomadura formosensis]	84/91	WP_067800070.1	kngH (81/87)
**orf15**	307	shikimate dehydrogenase [Actinomadura formosensis]	85/90	WP_067800073.1	kngI (81/86)
**orf16**	386	AHBA synthase [Actinomadura formosensis]	89/94	WP_067800076.1	kngK (88/93)
**orf17**	362	gfo/Idh/MocA family oxidoreductase [Micromonospora rifamycinica]	86/91	WP_067307269.1	kngL (80/88)
**orf18**	232	phosphoglycolate phosphatase [Micromonospora halophytica]	87/93	WP_091293655.1	kngM (86/90)
**orf19**	299	kanosamine kinase [Streptomyces katrae]	77/84	WP_030293822.1	kngN (78/86)
**orf20**	513	3-(3-hydroxyphenyl)propionate hydroxylase [Streptomyces katrae]	82/88	WP_030293823.1	kngR19 (74/81)
**orf21**	419	Cytochrome P450 [Micromonospora nigra]	72/84	SCL21807.1	kngR16 (71/83)
**orf22**	201	dTDP-4-keto-6-deoxy-D-glucose epimerase [Streptomyces lydicus]	71/81	WP_069568944.1	—
**orf23**	434	lipopolysaccharide biosynthesis protein RfbH [Actinomadura rifamycini]	85/92	WP_026404034.1	—
**orf24**	324	gfo/Idh/MocA family oxidoreductase [Actinomadura formosensis]	76/86	WP_084263057.1	—
**orf25**	146	aminoDHQ synthase [Actinomadura formosensis]	88/94	WP_067800109.1	kngJ (90/93)
**orf26**	442	aspartate aminotransferase family protein [Micromonospora nigra]	80/90	WP_091080812.1	—
**orf27**	376	UDP:flavonoid glycosyltransferase YjiC, YdhE family [Micromonospora rifamycinica]	68/84	SCG35582.1	kngR7 (57/76)
**orf28**	116	hypothetical protein [Actinokineospora enzanensis]	67/79	WP_018680276.1	—
**orf29**	303	nucleoside-diphosphate sugar epimerase [Actinomadura rifamycini]	84/93	WP_026403439.1	—
**orf30**	405	cytochrome P450 [A. mediterranei]	79/89	WP_013228164.1	kngR4 (49/65)
**orf31**	258	thioesterase [Micromonospora nigra]	78/85	WP_091080824.1	kngR (75/83)
**orf32**	420	cytochrome P450 [A. mediterranei]	88/93	WP_013222567.1	kngR5 (87/93)
**orf34**	408	LuxR family transcriptional regulator [Micromonospora rifamycinica]	73/82	SCG35671.1	kngR36 (68/76)
**orf35**	385	LuxR family transcriptional regulator [Micromonospora halophytica]	75/83	WP_091293625.1	kngR36 (68/77)

**orf36**	253	NDP-hexose 4-ketoreductase [Streptomyces sp. NRRL S-350]	77/86	WP_030241082.1	kng4 (81/87)
**orf37**	354	aldo/keto reductase [A. vancoresmycina]	84/90	WP_004562643.1	kng5 (84/90)
**orf38**	255	NDP-hexose 3-O-methyltransferase [A. vancoresmycina]	91/97	WP_004562642.1	kng6 (91/96)
**orf39**	383	dNTP-hexose glycosyl transferase [A. vancoresmycina]	77/87	WP_004562640.1	kngR7 (78/86)
**orf40**	230	transketolase [Salinispora arenicola]	81/88	WP_029021589.1	kngR15A (71/82)
**orf41**	312	transketolase [Actinomadura formosensis]	78/89	WP_067800099.1	kngR15B (72/84)
**orf42**	421	cytochrome P450 [A. mediterranei]	75/85	WP_014466580.1	kngR16 (72/82)
**orf43**	656	copper oxidase [Micromonospora halophytica]	85/90	WP_091298550.1	kng12 (70/80)
**orf44**	476	NDP-hexose 2,3-dehydratase [A. vancoresmycina]	89/95	WP_004562638.1	kngR18 (89/95)
**orf45**	429	acetyltransferase [Micromonospora nigra]	70/80	SCL21819.1	kngR20 (65/75)
**orf46**	386	cytochrome P450 [A. vancoresmycina]	77/87	WP_004562636.1	kng10 (78/86)

Supplementary Table 7. Gene annotations for the metagenomic gene cluster RifCon6 and corresponding genes from the *A. vancoresmycina kng* cluster.

Gene Name	Length [aa]	Putative Function [Homolog Sp.]	%id / %sm	Accession	A. vanc. homolog (%id / %sm)
**orf8**	459	crotonyl-CoA carboxylase/reductase [Actinokineospora enzanensis]	84/91	WP_033400639.1	—
**orf9**	348	gfo/Idh/MocA family oxidoreductase [Micromonospora nigra]	84/92	WP_091080786.1	kngS (82/89)
**orf10**	322	gfo/Idh/MocA family oxidoreductase [Micromonospora nigra]	69/78	WP_091080781.1	kngT (49/56)
**orf11**	397	cytochrome P450 [Actinomadura formosensis]	85/92	WP_067794733.1	kngR0 (83/91)
**orf12**	5188	polyketide synthase [Streptomyces albogriseolus]	72/79	AHD24374.1	kngA (75/82)
**orf13/14**	4875	polyketide synthase [Micromonospora halophytica]	73/80	WP_091298529.1	kngB (78/85)
**orf15**	1805	polyketide synthase [Micromonospora nigra]	77/86	WP_091090512.1	kngC (78/85)
**orf16**	1836	polyketide synthase [A. vancoresmycina]	78/85	WP_003102381.1	kngD (77/85)
**orf17/18**	3498	polyketide synthase [Micromonospora halophytica]	76/83	EOD65140.1	kngE (74/82)
**orf19**	258	N-acetyltransferase/amide synthase [Actinomadura formosensis]	82/90	WP_084263055.1	kngF (73/82)
**orf20**	61	response regulator [Vibrio coralliilyticus]	41/59	WP_095571655.1	—
**orf21**	350	aminoDHQ synthase [Actinomadura formosensis]	89/95	WP_067800067.1	kngG (86/92)
**orf22**	392	aminoDAHP synthase [Actinomadura formosensis]	84/91	WP_067800070.1	kngH (82/87)
**orf23**	284	shikimate dehydrogenase [Actinomadura formosensis]	84/89	WP_067800073.1	kngI (81/86)
**orf24**	386	AHBA synthase [Actinomadura formosensis]	89/94	WP_067800076.1	kngK (87/92)
**orf25**	356	gfo/Idh/MocA family oxidoreductase [Micromonospora rifamycinica]	86/91	WP_067307269.1	kngL (80/88)
**orf26**	232	phosphoglycolate phosphatase [Micromonospora halophytica]	88/93	WP_091293655.1	kngM (86/91)
**orf27**	299	kanosamine kinase [Streptomyces subrutilus]	77/86	WP_069922309.1	kngN (78/86)
**orf28**	513	3-(3-hydroxyphenyl)propionate hydroxylase [Streptomyces katrae]	82/88	WP_030293823.1	kngR19 (74/82)
**orf29**	396	cytochrome P450 [A. vancoresmycina]	76/85	WP_003062297.1	kngR4 (76/85)
**orf30**	1082	hypothetical protein [A. vancoresmycina]	82/87	WP_051767686.1	kng2 (82/87)
**orf31**	588	ABC transporter ATP-binding protein [A. vancoresmycina]	79/89	WP_051767685.1	kng14 (79/89)
**orf32**	594	ABC transporter ATP-binding protein [A. vancoresmycina]	80/89	WP_003071800.1	kng17 (80/89)
**orf33**	525	methylmalonyl-CoA mutase [A. vancoresmycina]	92/96	WP_033261449.1	kng18A (92/96)
**orf34**	136	cobalamin B12-binding domain-containing protein [A. vancoresmycina]	90/93	WP_003071791.1	kng18B (90/93)
**orf35**	519	acyl-CoA carboxylase subunit beta [A. vancoresmycina]	91/96	WP_004562633.1	kng11 (91/96)
**orf36**	248	thioesterase [Micromonospora rifamycinica]	79/87	WP_067307207.1	kngR (71/82)
**orf37**	420	cytochrome P450 [A. vancoresmycina]	85/92	WP_004562634.1	kngR5 (85/92)
**orf38**	408	regulatory protein, luxR family [Micromonospora rifamycinica]	71/82	SCG35671.1	kngR36 (67/77)
**orf39**	145	aminoDHQ synthase [Actinomadura formosensis]	89/94	WP_067800109.1	kngJ (91/96)
**orf40**	357	LuxR family transcriptional regulator [Micromonospora halophytica]	77/84	WP_091293625.1	kngR36 (69/77)
**orf41**	194	YceI family protein [Yuhushiella deserti]	62/76	WP_092529286.1	—
**orf42**	253	NDP-hexose 4-ketoreductase [Streptomyces sp. NRRL S-350]	78/87	WP_030241082.1	kng3 (78/85)
**orf43**	329	aldo/keto reductase [A. vancoresmycina]	86/92	WP_004562643.1	kng4 (86/92)

**orf44**	255	NDP-hexose 3-O-methyltransferase [A. vancoresmycina]	93/97	WP_004562642.1	kng6 (93/97)
**orf45**	326	stationary phase survival protein SurE [A. mediterranei]	76/82	WP_013225304.1	kng7 (73/83)
**orf46**	383	hypothetical protein [A. vancoresmycina]	81/91	WP_004562640.1	kngR7 (81/91)
**orf47**	215	transketolase [Salinispora arenicola]	83/90	WP_029021589.1	kngR15A (74/84)
**orf48**	316	transketolase [Actinomadura formosensis]	77/88	WP_067800099.1	kngR15B (72/83)
**orf49**	426	cytochrome P450 [A. mediterranei]	76/86	WP_014466580.1	kngR16 (72/83)
**orf50**	658	copper oxidase [Micromonospora halophytica]	81/88	WP_091298550.1	kng12 (73/84)
**orf51**	474	NDP-hexose 2,3-dehydratase [A. vancoresmycina]	89/94	WP_004562638.1	kngR18 (89/94)
**orf52**	419	acyltransferase [Actinomadura formosensis]	70/80	WP_067800088.1	kngR20 (67/77)
**orf53**	388	cytochrome P450 [A. vancoresmycina]	82/91	WP_004562636.1	kng10 (82/91)

Supplementary Table 8. Gene annotations for the metagenomic gene cluster RifCon10 and corresponding genes from the *A. vancoresmycina kng* cluster.

Gene Name	Length [aa]	Putative Function [Homolog Sp.]	%id / %sm	Accession	A. vanc. homolog (%id / %sm)
**orf26/27**	54	gfo/Idh/MocA family oxidoreductase [A. vancoresmycina]	88/91	WP_051767688.1	kngS (88/91)
**orf28**	259	gfo/Idh/MocA family oxidoreductase [A. kentuckyensis]	85/91	WP_086842324.1	kngT (85/90)
**orf29**	71	hypothetical protein [A. tolypomycina]	97/98	WP_091304002.1	kng35 (87/90)
**orf30**	396	cytochrome P450 [A. vancoresmycina]	97/97	WP_003060232.1	kngR0 (97/97)
**orf31/32**	2412	polyketide synthase [A. vancoresmycina]	91/93	WP_003060229.1	kngA (91/93)
**orf33/34**	2148	polyketide synthase [A. vancoresmycina]	90/94	WP_033261454.1	kngB (90/94)
**orf35**	1807	polyketide synthase [A. vancoresmycina]	91/93	WP_081911206.1	kngC (91/93)
**orf36**	1822	polyketide synthase [A. vancoresmycina]	94/95	WP_003102381.1	kngD (94/95)
**orf37**	3449	polyketide synthase [A. vancoresmycina]	93/95	WP_033261452.1	kngE (93/95)
**orf38**	261	N-acetyltransferase/amide synthase [A. vancoresmycina]	93/95	WP_004559807.1	kngF (93/95)
**orf39**	62	hypothetical protein [A. vancoresmycina]	89/93	WP_004559806.1	kngR1 (89/93)
**orf40**	351	aminoDHQ synthase [A. vancoresmycina]	94/96	WP_004559805.1	kngG (94/96)
**orf41**	418	aminoDAHP [A. vancoresmycina]	94/96	WP_004559804.1	kngH (94/96)
**orf42**	262	shikimate dehydrogenase [A. vancoresmycina]	92/94	WP_081911200.1	kngI (92/94)
**orf43**	66	tripartite tricarboxylate transporter substrate binding protein [Curvibacter sp. GWA2_64_110]	39/54	WP_053245989.1	—
**orf44**	386	AHBA synthase [A. vancoresmycina]	98/99	WP_003062304.1	kngK (98/99)
**orf45**	357	gfo/Idh/MocA family oxidoreductase [A. vancoresmycina]	94/96	WP_033261451.1	kngL (94/96)
**orf46**	232	phosphoglycolate phosphatase [A. vancoresmycina]	98/100	WP_003062302.1	kngM (98/100)
**orf47**	298	kanosamine kinase [A. vancoresmycina]	84/90	WP_003062299.1	kngN (84/90)
**orf48**	398	cytochrome P450 [A. vancoresmycina]	97/98	WP_003062297.1	kngR4 (97/98)
**orf49**	1058	hypothetical protein [A. vancoresmycina]	95/96	WP_051767686.1	kng2 (95/96)
**orf50**	253	NDP-hexose 4-ketoreductase [A. vancoresmycina]	95/96	WP_004562644.1	kng3 (95/96)
**orf51**	355	aldo/keto reductase [A. vancoresmycina]	95/96	WP_004562643.1	kng4 (95/96)
**orf52**	255	NDP-hexose 3-O-methyltransferase [A. vancoresmycina]	96/98	WP_004562642.1	kng6 (96/98)
**orf53**	324	stationary phase survival protein SurE [A. vancoresmycina]	93/96	WP_004562641.1	kng7 (93/96)
**orf54**	383	hypothetical protein [A. vancoresmycina]	94/96	WP_004562640.1	kngR7 (94/96)
**orf55**	474	NDP-hexose 2,3-dehydratase [A. vancoresmycina]	98/99	WP_004562638.1	kngR18 (98/99)
**orf56**	378	cytochrome P450 [A. vancoresmycina]	98/100	WP_004562636.1	kng10 (98/100)
**orf57**	421	cytochrome P450 [A. vancoresmycina]	97/98	WP_004562634.1	kngR5 (97/98)
**orf58**	531	acyl-CoA carboxylase subunit beta [A. vancoresmycina]	97/98	WP_004562633.1	kng11 (97/98)
**orf59**	665	copper oxidase [A. vancoresmycina]	92/95	WP_081911204.1	kng12 (92/95)
**orf60**	576	ABC transporter ATP-binding protein [A. vancoresmycina]	94/97	WP_051767685.1	kng14 (94/97)
**orf61**	594	ABC transporter ATP-binding protein [A. vancoresmycina]	96/98	WP_003071800.1	kng17 (96/98)
**orf62**	525	methylmalonyl-CoA mutase [A. vancoresmycina]	98/99	WP_033261449.1	kng18A (98/99)
**orf63**	131	cobalamin B12-binding domain-containing protein [A. vancoresmycina]	98/99	WP_003071791.1	kng18B (98/99)

**orf64**	344	CoA transferase [A. vancoresmycina]	96/97	WP_003071788.1	kng21 (96/97)
**orf65**	485	3-(3-hydroxyphenyl)propionate hydroxylase [A. vancoresmycina]	92/96	WP_003071786.1	kngR19 (92/96)
**orf66**	392	acyltransferase [A. vancoresmycina]	95/97	WP_033261516.1	kngR20 (95/97)
**orf67**	249	thioesterase [A. vancoresmycina]	92/95	WP_003071782.1	kngR (92/95)
**orf68**	422	cytochrome P450 [A. vancoresmycina]	98/100	WP_003071780.1	kngR13 (98/100)
**orf69**	231	transketolase [A. vancoresmycina]	98/99	WP_003071778.1	kngR15A (98/99)
**orf70**	303	transketolase [A. vancoresmycina]	98/98	WP_003071776.1	kngR15B (98/98)
**orf71**	419	cytochrome P450 [A. vancoresmycina]	97/98	WP_003071775.1	kngR16 (97/98)
**orf72**	149	aminoDHQ synthase [A. vancoresmycina]	95/96	WP_003071773.1	kngJ (95/96)
**orf73**	449	LuxR family transcriptional regulator [A. vancoresmycina]	94/97	WP_003071771.1	kngR36 (94/97)

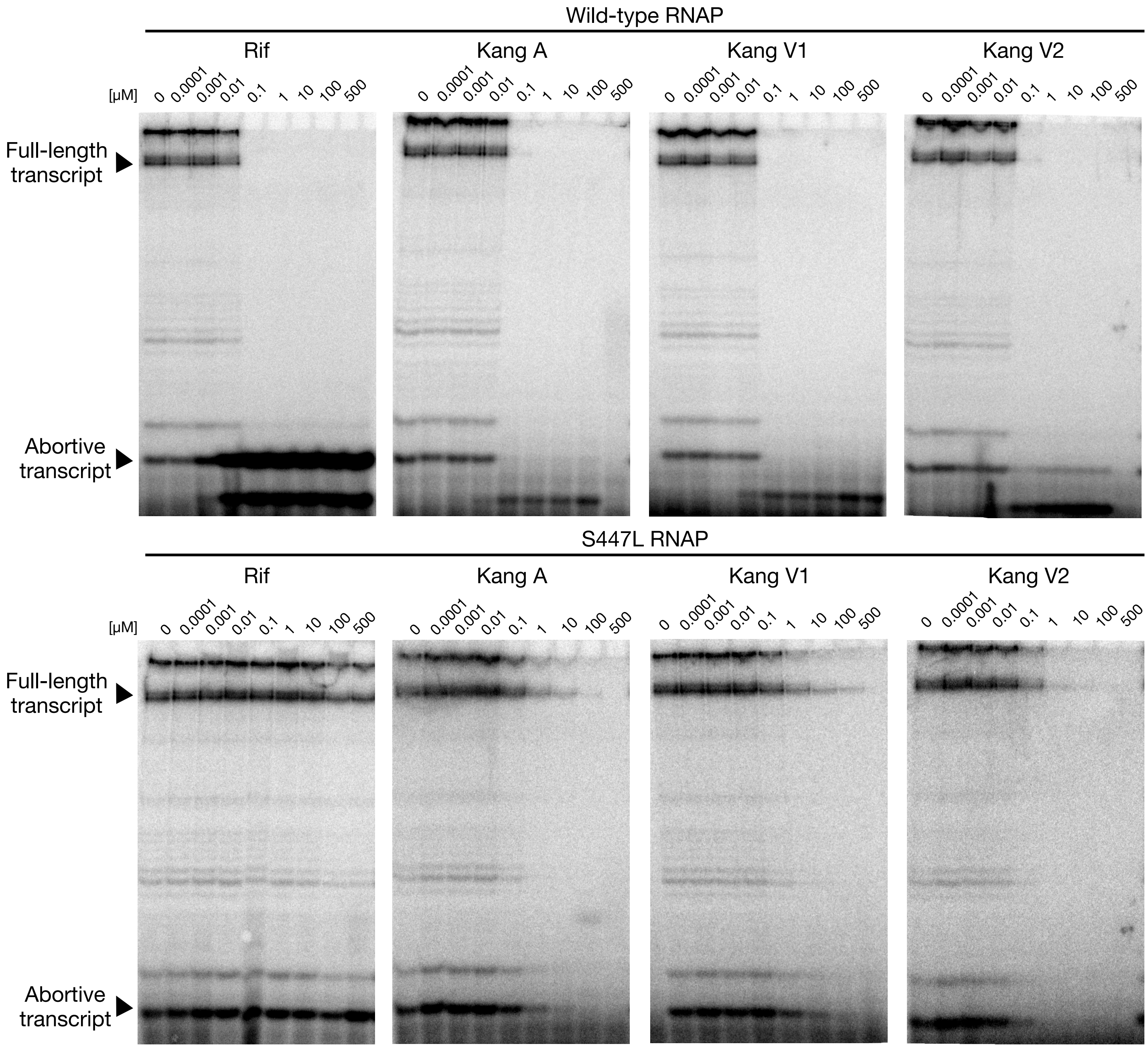

Supplementary Figure 29. Uncropped scans of transcription assay gels shown in manuscript Figure 3E.

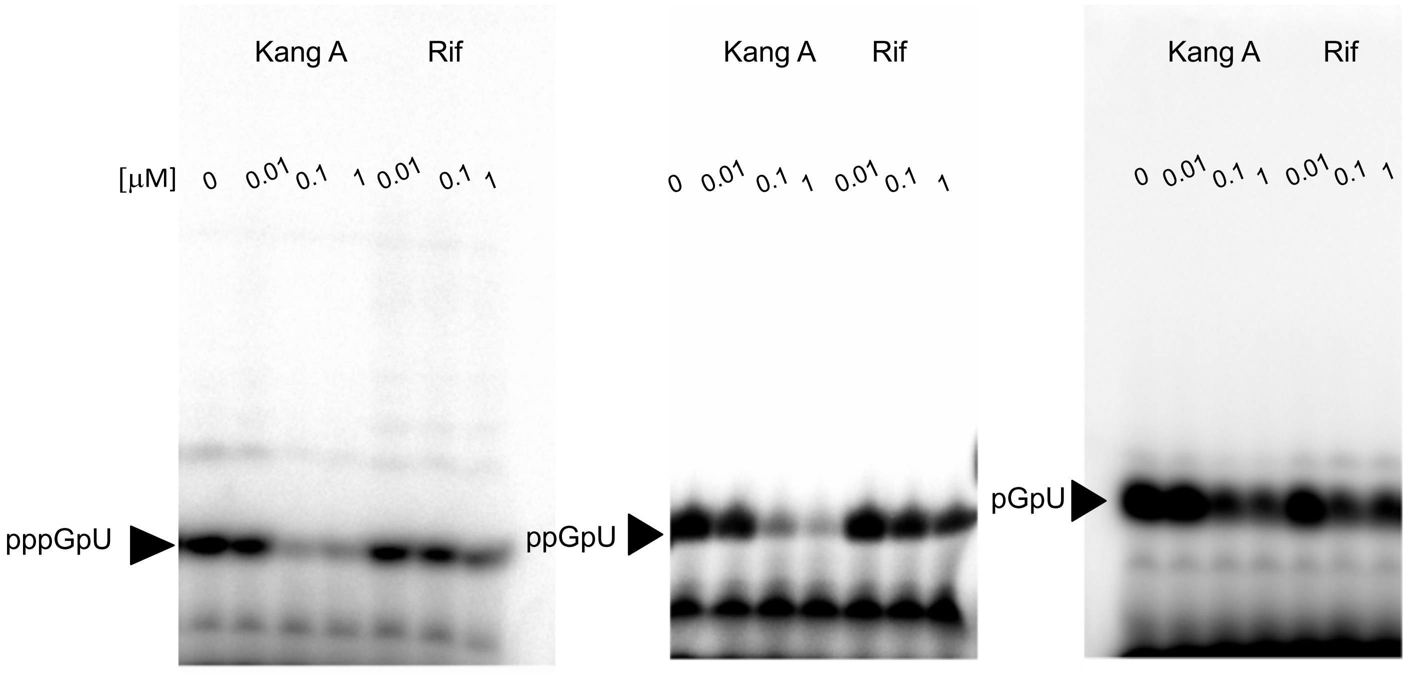

Supplementary Figure 30. Uncropped scans of transcription assay gels shown in manuscript Figure 6D.

References

1. Dunn, B.J. & Khosla, C. Engineering the acyltransferase substrate specificity of assembly line polyketide synthases. *J R Soc Interface* **10**, 20130297 (2013).
2. Minowa, Y., Araki, M. & Kanehisa, M. Comprehensive analysis of distinctive polyketide and nonribosomal peptide structural motifs encoded in microbial genomes. *J Mol Biol* **368**, 1500-1517 (2007).
3. Rateb, M.E. et al. Chaxamycins A-D, bioactive ansamycins from a hyper-arid desert Streptomyces sp. *J Nat Prod* **74**, 1491-1499 (2011).
4. Santos, L. et al. Structural characterization by NMR of rifabutinol, a derivative of rifabutin. *Magn Reson Chem* **38**, 937-945 (2000).
5. Santos, L. et al. NMR studies of some rifamycins. *J Mol Struct* **563**, 61-78 (2001).
6. Arora, S.K. & Kook, A.M. Structure and Conformation of Halomycin-B in Solid-State and Solution. *J Org Chem* **52**, 1530-1535 (1987).
7. Cellai, L. et al. Comparative-Study of the Conformations of Rifamycins in Solution and in the Solid-State by Proton Nuclear Magnetic-Resonance and X-Rays. *J Org Chem* **47**, 2652-2661 (1982).
8. Kishi, T., Asai, M., Muroi, M., Harada, S. & Mizuta, E. Tolypomycin. I. Structure of tolypomycinone. *Tetrahedron Lett*, 91-95 (1969).
9. Wang, N.J. et al. Isolation and structure of a new ansamycin antibiotic kanglemycin A from a Nocardia. *J Antibiot (Tokyo)* **41**, 264-267 (1988).
